# Comparison of Proteomic Responses as Global Approach to Antibiotic Mechanism of Action Elucidation

**DOI:** 10.1128/AAC.01373-20

**Published:** 2020-12-16

**Authors:** Christoph H. R. Senges, Jennifer J. Stepanek, Michaela Wenzel, Nadja Raatschen, Ümran Ay, Yvonne Märtens, Pascal Prochnow, Melissa Vázquez Hernández, Abdulkadir Yayci, Britta Schubert, Niklas B. M. Janzing, Helen L. Warmuth, Martin Kozik, Jens Bongard, John N. Alumasa, Bauke Albada, Maya Penkova, Tadeja Lukežič, Nohemy A. Sorto, Nicole Lorenz, Reece G. Miller, Bingyao Zhu, Martin Benda, Jörg Stülke, Sina Schäkermann, Lars I. Leichert, Kathi Scheinpflug, Heike Brötz-Oesterhelt, Christian Hertweck, Jared T. Shaw, Hrvoje Petković, Jean M. Brunel, Kenneth C. Keiler, Nils Metzler-Nolte, Julia E. Bandow

**Affiliations:** a Applied Microbiology, Faculty of Biology and Biotechnology, Ruhr University Bochum, Bochum, Germany; b Biochemistry and Molecular Biology, Pennsylvania State University, State College, Pennsylvania, USA; c Bioinorganic Chemistry, Faculty of Chemistry and Biochemistry, Ruhr University Bochum, Bochum, Germany; d Acies Bio, Ljubljana, Slovenia; e Department of Chemistry, University of California—Davis, Davis, California, USA; f Department of General Microbiology, Institute of Microbiology and Genetics, Georg-August University Göttingen, Göttingen, Germany; g Microbial Biochemistry, Institute of Biochemistry and Pathobiochemistry, Ruhr University Bochum, Bochum, Germany; h Peptide Lipid Interactions, Leibniz Institute for Molecular Pharmacology, Berlin, Germany; i Microbial Bioactive Compounds, Interfaculty Institute of Microbiology and Infection Medicine Tübingen, University of Tübingen, Tübingen, Germany; j Biomolecular Chemistry, Leibniz Institute for Natural Product Research and Infection Biology-Hans Knöll Institute, Jena, Germany; k Biotechnical Faculty, University of Ljubljana, Ljubljana, Slovenia; l UMR MD1, Membranes et Cibles Thérapeutiques, Faculté de Pharmacie U1261 INSERM, Aix-Marseille Université, Marseille, France

**Keywords:** antibiotic, chemical biology, mechanism of action, physiology, proteomics

## Abstract

New antibiotics are urgently needed to address the mounting resistance challenge. In early drug discovery, one of the bottlenecks is the elucidation of targets and mechanisms. To accelerate antibiotic research, we provide a proteomic approach for the rapid classification of compounds into those with precedented and unprecedented modes of action. We established a proteomic response library of Bacillus subtilis covering 91 antibiotics and comparator compounds, and a mathematical approach was developed to aid data analysis. Comparison of proteomic responses (CoPR) allows the rapid identification of antibiotics with dual mechanisms of action as shown for atypical tetracyclines. It also aids in generating hypotheses on mechanisms of action as presented for salvarsan (arsphenamine) and the antirheumatic agent auranofin, which is under consideration for repurposing. Proteomic profiling also provides insights into the impact of antibiotics on bacterial physiology through analysis of marker proteins indicative of the impairment of cellular processes and structures. As demonstrated for *trans*-translation, a promising target not yet exploited clinically, proteomic profiling supports chemical biology approaches to investigating bacterial physiology.

## Abstract (precis)

New antibiotics are urgently needed to address the mounting resistance challenge. In early drug discovery, one of the bottlenecks is the elucidation of targets and mechanisms. To accelerate antibiotic research, we provide a proteomic approach for the rapid classification of compounds into those with precedented and unprecedented modes of action. We established a proteomic response library of Bacillus subtilis covering 91 antibiotics and comparator compounds, and a mathematical approach was developed to aid data analysis.

## INTRODUCTION

Antibiotics are essential medicines that reduce the mortality as well as the economic and societal impacts of bacterial infections. In 1910, the first antibiotic introduced to the market was salvarsan (arsphenamine) for the treatment of syphilis. To this day, its mechanism of action has not been investigated. The use of salvarsan was discontinued due to toxicity when safer drugs like cell wall biosynthesis (CWB)-inhibiting β-lactams or protein biosynthesis-blocking tetracyclines became available. Numerous antibiotic classes were discovered during the “golden age of antibiotic discovery” (1940 to 1960). The most successful classes were brought to market and subsequently refined over several generations to reduce side effects, broaden the spectrum of activity, and overcome resistance. Antibiotic approvals peaked in the 1980s but declined since. In decades, no structurally new antibiotic class was discovered that entered the market (1). Today, multidrug-resistant bacteria like methicillin-resistant Staphylococcus aureus (MRSA) and Pseudomonas aeruginosa pose enormous challenges (2). With resistance spreading, infectious diseases returned as one of the leading causes of death worldwide, and it is estimated that by 2050, 10 million people will die annually due to antimicrobial resistance (3).

While antibiotic releases are still at a low, the investigation of bioactive molecules of natural and synthetic origins is experiencing a renaissance. Promising recently described compounds include teixobactin, a cyclic depsipeptide produced by Eleftheria terrae that inhibits cell wall biosynthesis (4), and murgocil, a synthetic inhibitor of cell wall biosynthesis that synergizes with β-lactams against MRSA (5). To find novel antibiotics, extensive compound libraries are screened for antibacterial activity (whole-cell activity screening) or for inhibition of a particular protein target or cellular process (target-based screening). Characterizing these initial hits is a bottleneck in antibiotic research, as it is laborious and resource-intensive. Therefore, it is important to identify promising lead structures early on, to focus resources. One of the impediments in characterizing hits from whole-cell activity screens (as opposed to target-based screens) is the time-consuming elucidation of the antibacterial target and mechanism of action. This is usually approached by identifying affected pathways by means of precursor incorporation (6), reporter gene assays (7), identifying targets based on mapping mutations in spontaneous resistant mutants (8), or investigating the effects of treatment of bacteria on a system scale by transcriptome or proteome analysis (6, 9, 10). Systems-based approaches are particularly useful to investigate the effects of compounds with novel molecular targets (stemming from either screening approach) on bacterial physiology as a whole, as was first shown by VanBogelen and Neidhardt (11).

Here, we provide a major update of the Bacillus subtilis proteomic response library, which had its origins in the late 1990s when large pharmaceutical companies investigated natural products as potential new antibacterial agents. It was shown that the acute proteomic response reflects the physiological impact of an antibiotic as well as cellular strategies to control and overcome the physiological challenge. Since compounds with a similar impact on physiology elicit similar responses, the proteomic profiles were used to identify inhibition of the peptidyl transferase reaction as the mechanism of action of the natural product Bay 50-2369 based on the similarities to chloramphenicol and tetracycline (10). Proteome analyses also aided in elucidating the mechanism of action of the natural product acyldepsipeptide, which targets ClpP (12). When industry largely moved to target-based antibiotic discovery, the library was expanded to include agents that inhibit experimental target areas such as fatty acid biosynthesis (FAB) (13) or, inspired by the clinical success of daptomycin, the bacterial membrane. In fact, proteome analysis contributed to a better understanding of the mechanism of action of daptomycin itself (14). The mounting antibiotic resistance challenge increases the urgency to find new antibacterial agents with novel targets and mechanisms of action. Recent, largely academic efforts led to the emergence of compounds that need mechanism-of-action analysis and innovative drug discovery projects that require a better understanding of the physiological impact of inhibiting a novel target. Recognizing this demand, we offer an approach we termed comparison of proteomic responses (CoPR) in support of antibiotic research.

To propel future mechanism-of-action studies and obtain insights into the physiology of antibiotic action, we gathered 55 of our previously reported proteomic profiles of Bacillus subtilis 168 and investigated 36 further proteomic responses. This adds up to a library of response profiles for 91 antibacterial agents and comparator compounds (see Data Set S1, Tables S1 to S71, and Fig. S1 to S35 in the supplemental material). The library covers clinically relevant and experimental drugs like the topoisomerase inhibitors ciprofloxacin and nalidixic acid, the aminoacyl-tRNA synthesis inhibitors mupirocin and AN3334 (15), the RNA polymerase inhibitor rifampicin, and the fatty acid biosynthesis inhibitors platensimycin (13) and platencin (16) and substances used in research, like the ionophores calcimycin and ionomycin, which disturb ion homeostasis (17). Our data have been made publicly available on the SubtiWiki platform (18) (http://subtiwiki.uni-goettingen.de/v4/downloads) and in the supplemental material (Data Set S1, Tables S1 to S71, and Fig. S1 to S35).

To aid the analysis of new compounds, we introduce an approach that we termed CoPR that combines two-dimensional PAGE (2D-PAGE) with a mathematical comparison of the response profiles. A step-by-step protocol on how to use CoPR is available in the supplemental material. CoPR allows the deduction of target areas for cytoplasmic, nonprotein, and extracytoplasmic targets. Marker proteins indicative of the impairment of a specific cellular process or structure were delineated. As examples, we investigated the mechanisms of atypical tetracyclines (which can have dual mechanisms), salvarsan, auranofin, an antirheumatic drug considered for repurposing, and *trans*-translation inhibitors targeting a process not yet clinically exploited (19).

## RESULTS

### Construction of the B. subtilis proteomic response library.

To build the proteomic response library, we chose the Gram-positive model organism B. subtilis 168, which is susceptible to most antibiotics, limiting constraints due to resistance, compound uptake, or efflux, which can impede this type of analysis for pathogens, in particular multiresistant pathogens. The proteome of B. subtilis has been investigated in depth (20), and an extensive knowledgebase exists on its regulatory circuits and protein functions, which provides an optimal basis for a thorough interpretation of proteomic responses. While it is not possible to directly transfer knowledge of the specific proteins upregulated in response to an antibiotic challenge from B. subtilis to pathogens, the inferred target area is not species specific, and hypotheses on target proteins can be tested in model organisms or pathogens.

**FIG 1:**
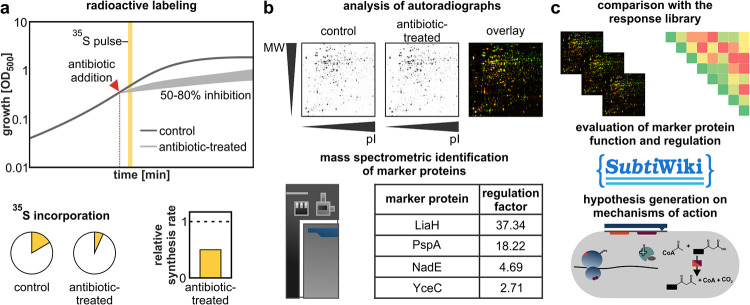
Construction of the Bacillus subtilis proteomic response library. (a) Sample generation. Newly synthesized proteins of B. subtilis are pulse-labeled for 5 min with l-[^35^S]methionine 10 min after the addition of an antibiotic at a concentration that leads to 50 to 80% growth inhibition. Subsequently, the incorporation of ^35^S into protein of control and antibiotic-treated samples is determined by scintillation counting to calculate relative protein synthesis rates. (b) Data generation. Autoradiographs of 2D gels are used for software-based image analysis to identify marker proteins (regulation factor of ≥2 in each of the biological replicates). Marker proteins are excised from nonradioactive gels and identified by mass spectrometry. MW, molecular weight; pI, isoelectric point. (c) Data evaluation. Identified marker proteins and regulation factors are added to the CoPR library to calculate a similarity matrix. The similarity of a proteomic profile to responses in the library is utilized, in conjunction with knowledge on the proteins (function, localization, and regulation, etc.) from the SubtiWiki database and the literature, to generate a hypothesis on the mechanism of action of compounds.

The goal of the proteome analysis is to capture the acute antibiotic impact and bacterial response to antibiotic treatment. From a technical perspective, the construction of the proteomic response library can be divided into three steps: sample generation (Fig. 1a), data generation (Fig. 1b), and data evaluation (Fig. 1c). To generate samples, exponentially growing cultures were treated with antibacterial agents in early to mid-log phase using “physiologically effective concentrations” (PECs) that inhibit the growth of the cultures by 50 to 80%. These concentrations were identified individually for each agent in growth experiments. Proteins produced during a 5-min pulse, starting 10 min after compound addition, were labeled with [^35^S]methionine, and the cells were harvested. Based on total protein quantitation and scintillation counting, global relative protein synthesis (PS) rates after antibiotic treatment were calculated in relation to untreated controls. Proteins were separated by 2D-PAGE, and relative synthesis rates were determined for individual protein spots based on the autoradiographs. To be designated “marker proteins,” the relative synthesis rates had to be at least 2 in each biological replicate. The exquisite sensitivity of pulse-labeling allows the monitoring of changes in the allocation of the cellular translation capacity, revealing adaptations of the proteome, which in gel-free mass spectrometry (MS)-based proteomic approaches remain hidden in a background of accumulated proteins (21). Working with the growing library of proteomic profiles required the development of a data evaluation concept (Fig. 1c). The mathematical approach to the comparison of proteomic responses (CoPR) is based on a matrix of pairwise comparisons of the similarity between two antibiotic responses. For each pairwise comparison, a cosine similarity score (CoPR score) was generated based on the regulation factors (RFs) of the marker proteins. The scores range from 1 (perfect similarity) to 0 (perfect dissimilarity), which allows the rapid identification of similar proteomic responses. Proteomic responses can be further interpreted individually based on the marker proteins, protein modifications, and knowledge on protein regulation and function. This is typically necessary to generate hypotheses on potential new molecular targets or to understand the physiological consequences of antibacterial action.

### The proteomic response library.

**FIG 2:**
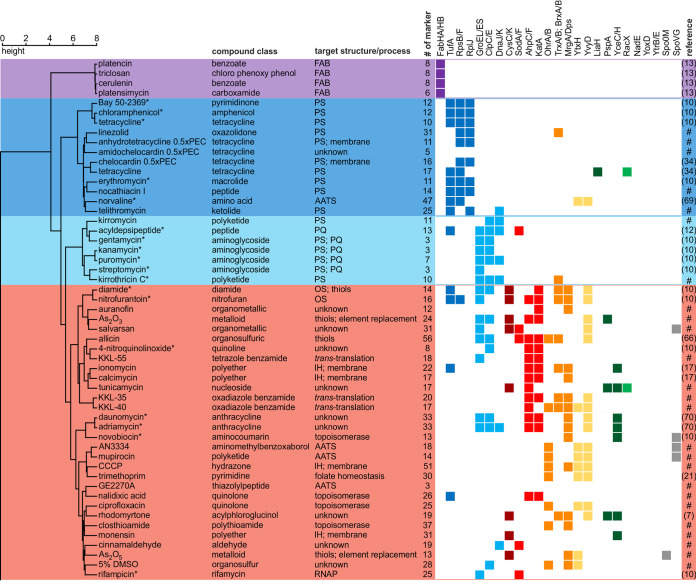
Similarity of proteomic responses to antibiotics affecting cytoplasmic targets and processes. B. subtilis was treated for 10 min with physiologically effective concentrations (PEC) of the agents prior to radioactive labeling of newly synthesized proteins and 2D-PAGE-based proteomic profiling. To generate the dendrogram, Ward’s method was applied to the CoPR scores (68). Abbreviations: AATS, aminoacyl-tRNA supply; IH, ion homeostasis; OS, oxidative stress; PQ, protein quality. Colors underlying the dendrogram indicate groups of antibiotics with similar antibiotic effects and responses: purple, inhibition of fatty acid biosynthesis (FAB); dark blue, inhibition of protein synthesis (PS); light blue, effects on PS resulting in proteotoxic stress; red, effects on redox homeostasis, metal homeostasis, and nucleic acids. Squares indicate marker proteins informative of cellular structures or processes according to the following color code: purple, FAB; dark blue, PS; light blue, proteotoxic stress; dark red, sulfur metabolism; red, detoxification of ROS; orange, prevention of oxidative damage; yellow, general stress; dark green, membrane (structural integrity); medium green, membrane (associated functions); gray, regulation of sporulation and cell division. References indicate the sources of proteomic data (7, 10, 12, 13, 17, 21, 34, 66, 69, 70). #, proteomic response recorded in this work; *, proteome response recorded on a different gel system.

**FIG 3:**
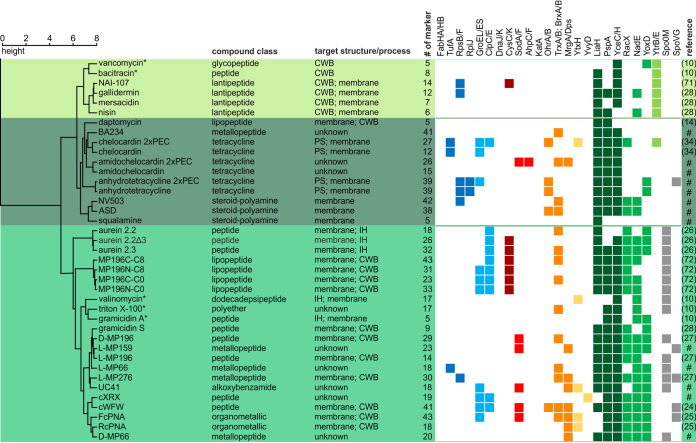
Similarity of proteomic responses to antibiotics affecting extracytoplasmic targets. B. subtilis was treated for 10 min with physiologically effective concentrations (PEC) of the antibacterial agents prior to radioactive labeling of newly synthesized proteins and 2D-PAGE-based proteomic profiling. To generate the dendrogram, Ward’s method was applied to the CoPR scores (68). IH, ion homeostasis. Colors underlying the dendrogram indicate groups of antibiotics with similar antibiotic effects and responses: light green, inhibition of cell wall biosynthesis (CWB) by interference with bactoprenol recycling; dark green, interference with membrane structure; medium green, interference with membrane structure and membrane-associated processes. Squares indicate marker proteins informative of cellular structures or processes according to the following color code: dark blue, protein synthesis (PS); light blue, proteotoxic stress; dark red, sulfur metabolism; red, detoxification of ROS; orange, prevention of oxidative damage; yellow, general stress; dark green, membrane (structural integrity); medium green, membrane (associated functions); light green, inhibition of CWB by interference with bactoprenol recycling; gray, regulation of sporulation and cell division. References indicate the sources of proteomic data (10, 14, 24–28, 34, 71, 72). #, proteomic response recorded in this work; *, proteome response recorded on a different gel system.

The library of proteomic responses covers clinically used antibiotics, experimental antibiotics, comparator compounds, and toxic substances. For some of the compounds, molecular targets and mechanisms have been described, while others have not been characterized yet (Fig. 2 and 3 and Table 1; see also Data Set S1 in the supplemental material). On average, the proteomic responses of B. subtilis comprise 20 different marker proteins, with the number of induced proteins ranging from 0 to 56. Overall, 486 different proteins were identified as marker proteins under at least one condition, 130 of which are proteins of unknown function.

**TABLE 1:** Antibiotics not amenable to CoPR analysis

Compound	Compound class	Process(es) and/or target structure	Description	Reference
Methicillin	Penicillin	Cell wall biosynthesis	No protein met the 2-fold upregulation requirement of marker proteins	10
Cephalexin	Cephalosporin	Cell wall biosynthesis, cell division	No protein met the 2-fold upregulation requirement of marker proteins	This work
PC190723	Benzamide	Cell division	No protein met the 2-fold upregulation requirement of marker proteins	This work
Rotenone	Isoflavone	Respiratory chain	B. subtilis 168 is resistant to rotenone, and no proteins met the 2-fold upregulation requirement	This work
Actinonin	Nonribosomal peptide	Protein synthesis, peptide deformylase	pI shift of protein spots after actinonin treatment prevents quantitative comparison of the proteomic profile to the control	32

**FIG 4:**
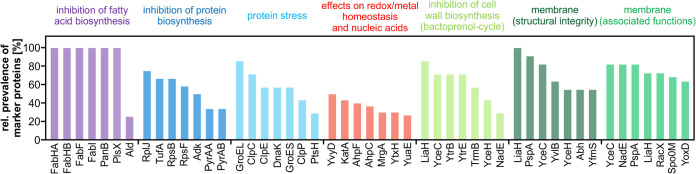
The most common marker proteins for each target area. For each branch of the dendrogram, the relative prevalence of the seven most frequently observed marker proteins is shown. The color code is indicated at the top.

The CoPR-based similarity analysis facilitated the mechanism-of-action-dependent sorting of 86 response profiles. The dendrogram built based on the CoPR scores separates into two main branches: compounds that mostly affect cytoplasmic components or processes (Fig. 2) and compounds with extracytoplasmic targets (Fig. 3). As discussed by VanBogelen and Neidhardt (11), proteins can be used as proteomic signatures to diagnose physiological states. In the context of antibiotics, they can specifically indicate which processes or structures are disturbed. The recurring marker proteins shown alongside the dendrogram (Fig. 2 and 3) allow the rapid matching of new compounds with target areas that are covered by compounds in the library. The homogeneity of proteomic responses varies for different cellular structures and pathways targeted (Fig. 4). Most compounds that impact the structural integrity of the cytoplasmic membrane, for instance, elicit the upregulation of certain marker proteins, most prominently LiaH, which is upregulated in response to 100% of these agents. The less well-defined target area of “redox and metal homeostasis and nucleic acid metabolism” is represented by more diverse responses with various combinations of marker proteins. The most consistent marker for this target area is YvyD, with approximately 50% representation.

Of all target areas, fatty acid biosynthesis inhibitors, which group into the first branch of the dendrogram, gave the most homogeneous response (Fig. 2 and 4). Irrespective of the protein target, the tested inhibitors (FabF inhibitors cerulenin and platensimycin, FabI inhibitor triclosan, and FabF, FabHA, and FabHB inhibitor platencin) resulted in the upregulation of FabHA, FabHB, FabF, and FabI (13). The induction of the fatty acid biosynthesis pathway is a direct countermeasure to the impairment of the pathway by the inhibitors.

Two neighboring branches comprise antibiotics that interfere with protein biosynthesis but affect physiology differently (Fig. 2). Antibiotics like tetracycline, erythromycin, or chloramphenicol interfere with chain elongation, thereby inhibiting protein biosynthesis. They share ribosomal proteins like RpsB, RpsF, or RplJ or elongation factor Tu (TufA) as markers. The upregulation of ribosomal proteins and TufA counteracts the reduction in protein synthesis rates. A different response is observed for agents that compromise translation fidelity and protein quality. Puromycin, which leads to the premature termination of translation; aminoglycosides, which interfere with ribosomal decoding and proofreading; and acyldepsipeptide, which causes uncontrolled proteolysis by ClpP (12), elicit the upregulation of the chaperone systems GroEL/GroES and DnaK/DnaJ as well as the proteases ClpC and ClpE. These proteins prevent the aggregation of misfolded proteins, facilitate refolding, or aid in the degradation of dysfunctional proteins.

Another branch harbors compounds that elicit oxidative stress responses, interfere with metal homeostasis, and/or cause nucleic acid stress (Fig. 2). Some of the agents, like nitrofurantoin and salvarsan, calcimycin and ionomycin, or nalidixic acid and rifampicin, elicit the upregulation of enzymes that detoxify reactive oxygen species (ROS), including the superoxide dismutases SodA and SodF, the catalase KatA, and/or the alkyl hydroperoxide reductase AhpC/AhpF. Especially for comparator compounds but also for the clinically used antibiotic nitrofurantoin, the upregulation of ROS-detoxifying proteins can occur together with the upregulation of chaperones and proteases, indicating that protein homeostasis is critically impaired. Some of the agents, like nitrofurantoin, calcimycin, novobiocin, mupirocin, and trimethoprim, elicit the upregulation of marker proteins involved in the protection of the cell from oxidative damage. Examples are MrgA and Dps, which mainly protect DNA, and/or OhrA/OhrB, the thioredoxin system (TrxA/TrxB), and BrxA/BrxB, which protect proteins. It was discussed previously that all antibiotics or all bactericidal antibiotics cause oxidative stress, which then leads to cell death (22, 23). Although upregulation of oxidative stress-related marker proteins occurs in response to many compounds in this branch as well as the branch covering extracytoplasmic functions, the library shows that, at least during the acute proteomic response with sublethal doses of antibiotics, this is not the case for all antibiotics or all bactericidal antibiotics. No ROS-responsive proteins were upregulated in response to most fatty acid biosynthesis inhibitors, protein biosynthesis inhibitors, or cell wall biosynthesis inhibitors, including bactericidal aminoglycosides, vancomycin, and methicillin.

The second main branch of the dendrogram contains compounds acting on the cell envelope and its functions (Fig. 3). The most prominent marker proteins for this branch are LiaH, PspA, and YceC/H. The paralogous proteins LiaH and PspA stabilize the membrane from the inside. PspA is upregulated in response to a loss of membrane integrity, and LiaH is upregulated in response to inhibition of membrane-based steps of cell wall biosynthesis. YceC and YceH are proteins of unknown function that were previously described as markers for cell envelope stress (24, 25). Other marker proteins that are frequently observed include RacX, NadE, and YoxD (26, 27). The amino acid racemase RacX is involved in cell envelope modification. The NAD^+^ synthase NadE has been described as a marker protein for membrane stress and indicates impairment of membrane-associated processes related to energy metabolism (27), and YoxD, a protein of unknown function, could be involved in the synthesis of alternative lipids, reorganizing the membrane to make it less susceptible to antimicrobial peptides (25, 27, 28).

The “extracytoplasmic” branch splits into three branches (Fig. 3). Compounds in the first branch are vancomycin, bacitracin, and lantibiotics (NAI-107, gallidermin, mersacidin, and nisin), all of which interfere with the function of lipid II and/or the bactoprenol cycle. They share YtrB and YtrE as additional markers. Both proteins are cytoplasmic components of a postulated ABC transporter known to be upregulated in early stationary phase (29). Based on the proteome data, we speculated that the ABC transporter has a function related to the bactoprenol cycle. Indeed, in support of this hypothesis, we found that a mutant that constitutively expresses the *ytrGABCDEF* operon is resistant to the acute effects of nisin (Fig. S36). The second branch includes compounds that interfere with membrane integrity but do not invoke sporulation-related marker proteins. Compounds in the third branch also target the membrane but share the sporulation- and cell division-associated marker Spo0M and the sporulation-associated transcriptional regulator SpoVG.

Not all antibiotics elicit an upregulation of marker proteins in B. subtilis. Neither the inhibition of late steps of cell wall biosynthesis nor the inhibition of cell division resulted in changes of the cytosolic proteome (Table 1). Accordingly, no marker proteins were observed for inhibition of the transpeptidase reaction by methicillin (10) or for inhibition of the cell division protein FtsZ or FtsI by PC190723 (30) and cephalexin, respectively. Also, no marker proteins were observed for rotenone, an electron transport chain inhibitor to which B. subtilis is intrinsically resistant (31) (Fig. S35). On the other end of the spectrum, some compounds have such a profound impact on the proteome that an interpretation of the 2D-PAGE-based proteomic response based on the CoPR approach is obstructed. When peptide deformylases (YkrB and Def) are inhibited by actinonin or downregulated in a conditional mutant, global interference with the cotranslational processing of protein N termini leads to a pI shift in most newly synthesized proteins (32). Using the approach described here, it is not possible to unambiguously match pI-shifted protein spots with spots under control conditions on autoradiographs of the 2D gels. While proteins could still be identified using mass spectrometry, the calculation of 2D-PAGE-based regulation factors is impaired. However, the pI shifts are a direct consequence of the antibiotic action and can thus be used as a proteomic signature that provides insights into the mechanism of action.

### The CoPR similarity matrix provides insights into dual mechanisms of action.

**FIG 5:**
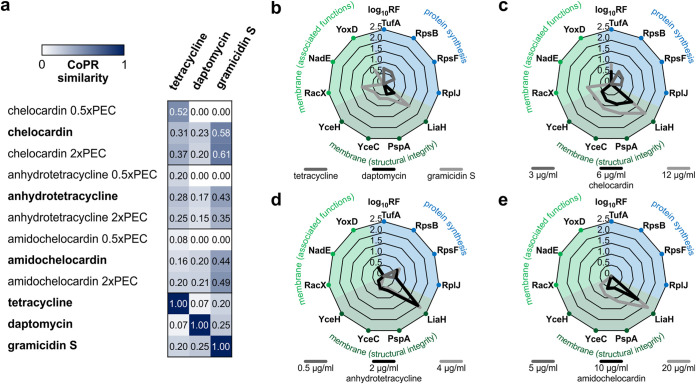
Concentration-dependent shifts in the mechanism of action of atypical tetracyclines. (a) Similarity of the proteomic responses of B. subtilis to different concentrations of tetracyclines and reference antibiotics. High similarity is indicated by a score close to 1 and a high intensity of blue color. Profiles originating from treatment with physiologically effective concentrations (PEC) are indicated in boldface type. (b) Upregulation of marker proteins in response to reference antibiotics. (c to e) Induction of marker proteins by different concentrations of chelocardin (c), anhydrotetracycline (d), and amidochelocardin (e). For panels b to e, the scale represents the log_10_ of the marker protein regulation factor. For panels c to e, the black lines correspond to treatment with the PEC. For proteomic responses to amidochelocardin and anhydrotetracycline, see Fig. S4 and S5 in the supplemental material. Proteomic profiles for tetracycline and chelocardin were taken from a study by Stepanek et al. (34), and profiles for daptomycin and gramicidin S were taken from studies by Müller et al. (14) and Wenzel et al. (28). All sections reflect averages from three biological replicates.

The CoPR approach allows the rapid detection of compounds with potential dual mechanisms of action since they elicit proteomic responses that show similarity to the profiles of compounds with two different target areas. Such compounds are strong candidates for clinical use because they might slow resistance development or lead to an overall bactericidal effect (33). In a recent proteome-based study, we showed that the atypical tetracycline chelocardin has a dual mechanism of action with a concentration-dependent differential impact on physiology (34). Taking chelocardin as an example, the CoPR matrix reflects the concentration-dependent similarity with protein synthesis inhibitors as well as compounds in the extracytoplasmic branch (Fig. 5a). At the physiologically effective concentration, chelocardin shares key marker proteins with both, as depicted exemplarily for tetracycline, daptomycin, and gramicidin S (Fig. 5b and c). The same is true for the atypical tetracycline anhydrotetracycline (Fig. 5d): it also shares marker proteins at the physiologically effective concentration with protein synthesis inhibitors and with compounds affecting the cytoplasmic membrane. Interestingly, the proteomic response to 2-carboxamido-2-deacetyl-chelocardin (amidochelocardin), a recently described derivative of chelocardin that is active against multidrug-resistant pathogens (35), does not show indications of a dual mechanism (Fig. 5e). No marker proteins characteristic of protein synthesis inhibition were upregulated in response to amidochelocardin, even at low concentrations. In congruence with the proteomic response, the protein synthesis rates dropped to 50% at 12 μg/ml chelocardin (34), as measured by the incorporation of [^35^S]methionine, but were unaffected by amidochelocardin even at concentrations of up to 20 μg/ml.

### Metals and metalloids: opportunities for extending the elemental building set for antibiotics.

One approach to quickly introducing new antibiotics is to repurpose medicines approved for other indications. A promising candidate for drug repurposing is auranofin, a late-stage antirheumatic drug. Auranofin, a gold-based organometallic, has been shown to inhibit antibiotic-resistant pathogenic Gram-positive bacteria like MRSA (36). It is an inhibitor of thioredoxin (37) and is thought to exert its antirheumatic effects through interactions with cysteines in transcription factors like AP-1 or NF-κB. While auranofin’s antibacterial mechanism is not fully elucidated, thioredoxin is indeed an essential protein in B. subtilis (38).

Another compound that contains an element rarely used in medicines is the arsenic-based organometalloid salvarsan. While its mechanism has not been proven experimentally, it is believed to be based on the reaction of arsenic with thiols. Salvarsan’s use for the treatment of syphilis was discontinued when safer antibiotics became available. However, with resistance of Treponema pallidum to second-line macrolide antibiotics on the rise and penicillin resistance impending (39), salvarsan might be worth revisiting as an antibiotic or an inspiration for the design of new drugs.

**FIG 6:**
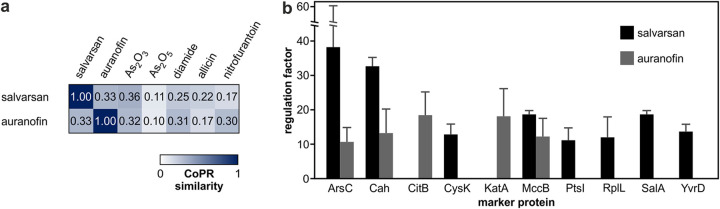
Comparing proteomic responses of B. subtilis 168 to auranofin and salvarsan. (a) Similarity of the proteomic responses of B. subtilis against salvarsan, auranofin, arsenic salts, and reference antibiotics. High similarity is indicated by a score close to 1 and a high intensity of blue color. (b) Comparison of marker proteins of salvarsan and auranofin. Shown are the marker proteins with the highest regulation factors for both compounds. Averages from three biological replicates with standard deviations are given for the marker proteins of each antibiotic (regulation factors of ≥2 in all replicates).

We investigated the mechanisms of action of auranofin and salvarsan by proteomic profiling using the arsenic salts As_2_O_3_ and As_2_O_5_ as comparator compounds. The CoPR similarity matrix revealed that the responses to auranofin, salvarsan, and As_2_O_3_ were similar (Fig. 6a). The CoPR scores also reveal a high similarity to nitrofurantoin, an oral antibiotic used to treat lower urinary tract infections, the mechanism of which is not fully elucidated. Among the marker proteins of auranofin and salvarsan was the arsenate reductase ArsC (Fig. 6b), which aids in the detoxification of arsenate. Both salvarsan and auranofin elicit a number of marker proteins that are involved in protecting the cell from oxidative protein damage (KatA, SodA, MrgA, chaperones, and PepF) or replenishing the cysteine pool (MccB, CysC, CysK, YrhB, YxeK, and YxeP) (Fig. 6b). These marker proteins and the high similarity to the diamide and allicin proteomic responses (both compounds are known to react with cysteines [40, 41]) corroborate that the mechanisms of action of auranofin and salvarsan likely involve direct or indirect thiol targeting.

### Proteomic profiling of *trans*-translation inhibitors.

Compound classes that exploit novel target areas are highly sought after to combat resistant pathogens. One promising novel target area is ribosome rescue, a process required to release ribosomes from stalled translation complexes. In bacteria, the most important ribosome rescue process is *trans*-translation, which not only releases the ribosome but also targets the defective mRNA and the incomplete nascent peptide for degradation (42). In some bacteria, including Escherichia coli and B. subtilis, alternative factors have been found that can release ribosomes but do not eliminate the mRNA or nascent peptide (43–45). For E. coli, a conservative estimate is that 2 to 4% of translation reactions require *trans*-translation to release ribosomes (46). Stalled ribosomes can quickly lead to the depletion of actively translating ribosomes by trapping other ribosomes in polysomes. We investigated the *trans*-translation inhibitors KKL-35 and KKL-40 (oxadiazoles), derivatives of which have been shown to cross-link to the 23S rRNA (47), and KKL-55 (48) (tetrazole), which likely has a different molecular target.

**FIG 7:**
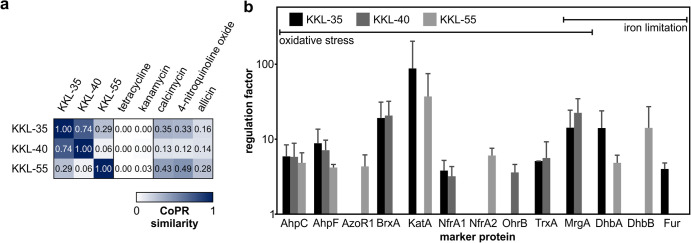
Proteomic response of B. subtilis 168 to inhibition of *trans*-translation. (a) Similarity matrix comparing proteomic responses of B. subtilis to the indicated compounds. Coloring indicates similarity, with high scores being close to 1 and indicated by a high intensity of blue color. (b) Comparison of marker proteins of *trans*-translation inhibitors. Shown are regulation factors of marker proteins related to responses against oxidative stress and iron limitation. Averages from three biological replicates with standard deviations are given for the marker proteins of each antibiotic (regulation factors of ≥2 in all replicates).

Decreased protein synthesis rates were observed after treatment with the oxadiazoles (KKL-35, 36%; KKL-44, 33%) but not to the same extent for the tetrazole (KKL-55, 83%). According to the CoPR similarity matrix, the proteomic profiles show low to no similarity to the profiles of either of the two groups of protein biosynthesis inhibitors, represented by tetracycline (inhibition of the elongation phase of translation) or kanamycin (causing proteotoxic stress). Instead, the inhibitors of *trans*-translation evoke a response similar to those of ionophores that disturb metal homeostasis (calcimycin) and other agents causing oxidative stress responses (4-nitroquinoline oxide and allicin) (Fig. 7a). Several marker proteins of *trans*-translation inhibitors are indicative of oxidative stress and iron limitation (Fig. 7b), responses that are coregulated by PerR and Fur in B. subtilis (reviewed by Moore and Helmann [49]). As bacteria often upregulate compensatory measures, we hypothesize that *trans*-translation is of particular importance under oxidative stress, a condition shown to lead to elevated levels of defective mRNAs (reviewed by Wurtmann and Wolin [50]).

## DISCUSSION

To support antibiotic research, we offer a system-based “comparison of proteomic responses” (CoPR) approach that provides insights into the impact of antibiotics on bacterial physiology and the bacterial strategies to cope with antibiotics. The CoPR library currently comprising responses of B. subtilis to 86 compounds as well as a step-by-step guide for comparative analysis are publicly accessible (http://subtiwiki.uni-goettingen.de/v4/downloads) (see Tables S1 to S71, Fig. S1 to S35, and Data Set S1 in the supplemental material).

The CoPR approach replaces labor-intensive one-on-one comparisons with an expedient and reliable process that identifies the most closely matching responses. The analysis of the response to tetracycline is a demonstration of the robustness of the method across laboratories and long time frames: the response to tetracycline was analyzed twice using two different protocols and different equipment as described in Materials and Methods and previous studies (10, 34) almost 15 years apart. Both response profiles group into the same target area in the dendrogram (Fig. 2). Much like the microscopy-based mechanism-of-action investigation introduced by Nonejuie et al. (51), CoPR provides a means of grouping compounds by target area and mechanism (Fig. 2 and 3) without directly disclosing molecular targets or mechanisms of action. In addition, the proteome profiles provide complementary information by providing detailed insights into the effects of antibiotics and bacterial stress responses on the system level.

As demonstrated for the atypical tetracyclines, compounds with dual mechanisms can be rapidly identified using the CoPR approach (Fig. 5). Chelocardin, which was investigated in a phase II clinical trial in 1977, is active against tetracycline-resistant pathogens (35). The biosynthetic gene cluster of Amycolatopsis sulphurea was recently engineered for the synthesis of derivatives (52). The dual mode of action of chelocardin was first described based on proteome profiling (34). At low concentrations, the proteomic profile is more similar to that of the protein biosynthesis inhibitor tetracycline, indicating that protein synthesis is inhibited, while with rising concentrations, the similarity to membrane-targeting compounds like daptomycin increases. In the original study, deriving this conclusion required elaborate one-on-one comparisons to each proteomic profile in the library. CoPR scoring now rapidly revealed that anhydrotetracycline also has a dual mechanism of action, while no marker proteins of protein synthesis are upregulated in response to amidochelocardin. Structurally, anhydrotetracycline is very similar to tetracycline, while amidochelocardin is very similar to chelocardin, showing that CoPR can distinguish dual mechanisms even between closely related compounds.

Aside from assessing the novelty of antibiotic mechanisms, proteome profiling can also serve to inform structure-activity relationship studies, as was shown for PM47, a derivative of the fatty acid biosynthesis inhibitor platensimycin, for which proteome analysis revealed that it no longer inhibits fatty acid biosynthesis (13). Proteome analysis also offers a starting point for mechanism-of-action elucidation of compounds with unprecedented targets. B. subtilis often reacts to the stress exerted by an antibiotic by inducing proteins that counteract the disturbance of cellular homeostasis (10, 13). The simplest reaction is the compensation of the loss of a particular function by the upregulation of the target, as observed, for example, for translation inhibitors, tRNA synthetase inhibitors, acyldepsipeptide (ClpP), and fatty acid biosynthesis inhibitors (10, 12, 13).

In previous analyses, the in-depth interpretation of proteome profiles based on marker proteins has proven particularly useful for generating hypotheses on the mechanism of action of antibiotics with nonprotein targets, like daptomycin or antimicrobial peptides, which act on the cytoplasmic membrane (14, 27). Here, we used proteomic profiling to investigate metal- and metalloid-containing antibiotics, revisiting the first antibiotic, salvarsan, and investigating auranofin, an antirheumatic drug with potential for repurposing. In 2005, 100 years after its discovery, the chemical structure of salvarsan was finally solved (53). Its antimicrobial mechanism is believed to be based on arsenic but has not been confirmed experimentally. Almost 40% of the marker proteins for salvarsan are related to the detoxification of arsenic, replenishment of the cysteine pool, and reaction to toxic protein modifications. Combining the information on marker proteins and the similarity of the salvarsan proteomic profile to that of As_2_O_3_, those of diamide and allicin (protein-thiol-modifying agents [40, 41]), and that of auranofin (described to inhibit the bacterial thioredoxin reductase [37]), we suggest that the antibiotic mechanism of salvarsan involves the targeting of protein thiols (Fig. 6). Interestingly, the physiologically effective concentrations of auranofin, salvarsan, and As_2_O_3_ are vastly different. The physiologically effective concentration of auranofin is 0.06 μg/ml (88 nM), congruent with a single or a very small group of thiol-containing protein targets, such as thioredoxin reductase. The physiologically effective concentration of As_2_O_3_ (64 μg/ml [647 μM]) is on the order of that of diamide (170 μg/ml [998 μM]), which reacts broadly with all thiols (40). The physiologically effective concentration of salvarsan is 2 μg/ml (10.8 μM), almost 1 order of magnitude lower than that of allicin (14 μg/ml [86 μM]). It remains to be investigated if the comparably good antibacterial activity of salvarsan over As_2_O_3_ and diamide is due to the improved delivery of the active compound to the target(s) in the cell or if salvarsan increases the selectivity for certain critical protein thiols.

Complementary to target-centric approaches, our system-based approach provides insights into the physiological impact of antibiotics. This can be exploited to study the physiological importance or the mechanism of a cellular process. Many aspects of ribosome function were elucidated using antibiotics. In such a chemical biology approach, antibiotics are used to cause a rapid loss of a critical cellular function to then observe the cellular response and deduce the impact of the loss of function on physiology. We employed *trans*-translation inhibitors from a recent compound screen (19) to study the physiology of ribosome rescue in B. subtilis. Many of the marker proteins are indicative of oxidative stress and a disturbance of metal ion homeostasis (Fig. 6). Since compensatory measures are often induced in response to antibiotic stress, we hypothesize that ribosome rescue is of particular importance for the ability of B. subtilis to deal with mRNAs affected by oxidative stress. It has been shown that mRNAs are prime targets of oxidation by reactive oxygen species and that ribosomes stall on oxidized mRNAs when decoding is prevented (50, 54).

As the study on *trans*-translation inhibitors shows, there is still much to learn about basic cellular processes. The presented library of proteomic response profiles reveals other gaps in our current understanding of bacterial physiology in general and the responses to antibiotics in particular. Although we based this study on the well-characterized model organism B. subtilis, across all proteome responses, 27% of the marker proteins have uncharacterized or understudied functions. The compilation of profiles enabled the deduction of marker proteins indicative of an antibiotic’s main target area by association. Among those with high regulation factors are, for example, YtrB/YtrE and YceC as marker proteins for agents with extracytoplasmic targets. Antibiotics might contribute to elucidating their functions. YtrB and YtrE, for instance, are cytoplasmic components of an ABC transporter that we found to be upregulated in response to lantibiotics. We showed that the constitutive production of the ABC transporter protects cells from the inhibitory effect of nisin. As this example shows, in some instances, follow-up experiments might reveal strategies of B. subtilis and perhaps related pathogens like Bacillus anthracis or S. aureus to cope with antibiotic stress and stress in general.

Proteomic profiling provides valuable insights into the effects of antibiotics on bacterial physiology and bacterial strategies to overcome antibiotic stress. The CoPR approach maximizes the utility of proteomic profiling by facilitating rapid comparisons of proteomic responses. We highlighted a variety of uses ranging from antibiotic mechanism-of-action studies, to investigations of bacterial physiology using antibiotics in a chemical biology approach, to analyses of gene function. Future applications may include the characterization of the bacterial response to nonantibiotic drugs, which have recently been shown to impact human commensals (55), or investigations of the effects of secreted metabolites on microorganisms that share a habitat (17). By making available the data and tools, we expect to expand the use of the technique across the scientific community.

## MATERIALS AND METHODS

### Antibacterial agents.

If not stated otherwise, substances were dissolved in dimethyl sulfoxide (DMSO). 2-Carboxamido-2-deacetyl-chelocardin (amidochelocardin) was supplied by T. Lukezic and H. Petkovic. AN3334 was provided by Anacore Pharmaceuticals Inc., Palo Alto, CA. Anhydrotetracycline, GE2270A, linezolid, nalidixic acid, and nocathiacin I were purchased from Merck KgaA, Darmstadt, Germany. Nalidixic acid was dissolved in H_2_O. As_2_O_3_ and As_2_O_5_ were purchased from Alfa Aesar, Haverhill, MA. As_2_O_3_ was dissolved in 1 M NaOH and As_2_O_5_ in distilled H_2_O. ASD was synthesized/purified as described previously by Salmi et al. (56) and dissolved in 0.9% NaCl. Auranofin was purchased from Fisher Scientific, Hampton, NH. BA234 was prepared according to previously described procedures (57), using osmocene carboxylic acid instead of ruthenocene carboxylic acid. Cephalexin, carbonyl cyanide *m*-chlorophenylhydrazone (CCCP), cinnamaldehyde, ciprofloxacin, monensin, mupirocin, rotenone, and tunicamycin were purchased from Sigma-Aldrich, St. Louis, MO. Ciprofloxacin was dissolved in 0.1 M NaOH. Monensin was dissolved in ethanol. Closthioamide was synthesized as described previously by Kloss et al. (58). cXRX was supplied by K. Scheinpflug. Kirromycin was supplied by S. Grond. KKL-35, KKL-40, and KKL-55 were supplied by K. C. Keiler. MP66 and MP159 were prepared as described previously by Albada et al. (59). NV503 was synthesized as described below. PC190723 was prepared as described previously (60). Salvarsan was prepared as described previously by Christiansen and Fargher (61, 62). Squalamine was synthesized/purified as described previously by Zhang et al. (63) and dissolved in H_2_O. Telithromycin was provided by Anacor Pharmaceuticals. UC41 was synthesized as described previously by Czaplewski et al. (60).

### Synthesis of NV503.

All solvents were purified according to previously reported procedures, and the reagents used were commercially available. Methanol (MeOH), ethyl acetate, and dichloromethane were purchased from Carlo Erba Reagents (Val de Reuil, France) and used without further purification. Column chromatography was performed on Carlo Erba Reagents silica gel (70 to 230 mesh). ^1^H nuclear magnetic resonance (NMR) and ^13^C NMR spectra were recorded in deuterated methanol (CD_3_OD) on a Bruker AC 300 spectrometer working at 300 MHz and 75 MHz, respectively (s, singlet; d, doublet; t, triplet; q, quadruplet; m, multiplet). Tetramethylsilane was used as the internal standard. All chemical shifts are given in parts per million. Mass spectroscopy analyses were performed by Spectropole (Analytical Laboratory) of Paul Cézanne University (Marseille, France). The purity of the compounds was checked by analytical high-performance liquid chromatography (HPLC) (C_18_ column, eluent of CH_3_CN-water-trifluoroacetic acid [TFA] [90:10:0.025, vol/vol/vol], and 0.5 to 1 ml/min) with a photodiode array (PDA) detector spanning from 210 nm to 310 nm. All compounds possessed purity above 95%, as determined by analytical HPLC-PDA analysis at 214 and 254 nm.

A mixture of progesterone (123 mg; 0.39 mmol), titanium(IV) isopropoxide (573 μl; 2.02 mmol), and spermine (2.02 mmol) in absolute methanol (5 ml) was stirred under argon at room temperature for 12 h. Sodium borohydride (114 mg; 3 mmol) was then added at −78°C, and the resulting mixture was stirred for an additional 2 h. The reaction was then quenched by adding water (1 ml) to the mixture, and stirring was maintained at room temperature for 20 min. The resulting inorganic precipitate was filtered off over a pad of Celite and washed with methanol and ethyl acetate. The combined organic extracts were dried over Na_2_SO_4_, filtered, and concentrated *in vacuo* to afford the expected crude amino derivative, which was purified by flash chromatography to afford the expected amino derivative. Purification by column chromatography (silica gel; CH_2_Cl_2_-MeOH-NH_4_OH [32%], 7:3:1) afforded a pale-yellow solid in 63% yield. ^1^H NMR (300 MHz, CD_3_OD) δ 5.28 (s, 1H), 3.69 to 3.54 (m, 1H), 3.13 (m, 1H), 2.78 to 2.68 (m, 15H), 2.32 to 0.76 (m, 40H). ^13^C NMR (75 MHz, CD_3_OD) δ 148.60, 128.04, 71.22, 67.32, 59.50, 57.61, 56.24, 50.90, 45.96, 43.79, 41.01, 38.53, 33.45, 30.80, 28.59, 27.21, 25.55, 24.27, 22.63, 19.99, 15.91, 13.21. C_31_H_58_N_4_O mass spectrometry (MS) with electrospray ionisation in positive mode (ESI^+^) *m/z* 503.4673 (100%, [M + H]^+^).

### Proteome analysis.

Bacillus subtilis 168 (*trpC2*) was cultured in Belitzky minimal medium (64) as described previously (10) and exposed at an optical density at 500 nm (OD_500_) of 0.35 to the following antibacterial agents at the indicated concentrations: amidochelocardin at 5, 10, and 20 μg/ml; AN3334 at 3 μg/ml; anhydrotetracycline at 0.5, 2, and 4 μg/ml; As_2_O_3_ at 64 μg/ml; As_2_O_5_ at 40 μg/ml; ASD at 2 μg/ml; auranofin at 0.06 μg/ml; BA234 at 6.25 μg/ml; CCCP at 0.5 μg/ml; cephalexin at 0.0675 μg/ml; cinnamaldehyde at 90 μg/ml; ciprofloxacin at 25 μg/ml; closthioamide at 0.7 μg/ml; cXRX at 16 μg/ml; GE227A at 8 μg/ml; kirromycin at 50 μg/ml; kirrothricin C at 100 μg/ml; KKL-35 at 1 μg/ml; KKL-40 at 0.25 μg/ml; KKL-55 at 2 μg/ml; KKL-896 at 1 μg/ml; linezolid at 0.1 μg/ml; monensin at 3 μg/ml; l-MP66 at 5.25 μg/ml; d-MP66 at 3.75 μg/ml; MP159 at 5 μg/ml; mupirocin at 0.06 μg/ml; nalidixic acid at 250 μg/ml; nocathiacin I at 0.15 μg/ml; NV503 at 4.2 μg/ml; PC190723 at 64 μg/ml; rotenone at 20 μg/ml; salvarsan at 2 μg/ml; squalamine at 1 μg/ml; telithromycin at 5 μg/ml; tunicamycin at 25 μg/ml; and UC41 at 8 μg/ml.

In this study, pulse-labeling with l-[^35^S]methionine and the 2D-PAGE-based proteome analysis were performed as described previously by Wenzel et al. (13), with the exception of kirrothricin C, which was analyzed as described previously by Bandow et al. (10). Briefly, for radioactive labeling with l-[^35^S]methionine, 5 ml of the bacterial culture was treated with an antibiotic for 10 min before 1.8 MBq radioactive methionine (Hartmann Analytic, Braunschweig, Germany) was added. Incorporation was stopped after 5 min by the addition of 1 mg/ml chloramphenicol and an excess of nonradioactive l-methionine and cooling of the cells on ice. Cells were harvested and washed before cell disruption using a VialTweeter sonicator (Hielscher, Teltow, Germany). Cell debris was removed by centrifugation, and protein concentrations were estimated using Roti NanoQuant (Roth, Karlsruhe, Germany). For radioactive gels, 50 μg of protein (300 μg for nonradioactive gels) was loaded onto 24-cm immobilized pH gradient strips, pH 4 to 7 (GE Healthcare, Little Chalfont, United Kingdom), by passive rehydration for 18 h. Proteins were separated by isoelectric focusing in the first dimension using a Multiphore II electrophoresis system (GE Healthcare). In the second dimension, proteins were separated according to molecular size by SDS-PAGE using the Ettan DaltTwelve system (GE Healthcare). Under a few antibiotic conditions (marked with an asterisk in the figures), the Millipore 2D gel electrophoresis system (Merck KgaA) was used for SDS-PAGE. Radioactive gels were dried on Whatman paper and exposed to storage phosphor screens (GE Healthcare). Screens were scanned using a Typhoon Trio^+^ instrument (GE Healthcare) with a 633-nm excitation wavelength and a 390-nm emission filter. Nonradioactive gels were stained with 0.003% ruthenium(II)-tris(4,7)diphenyl-1,10-phenanthroline disulfonate and scanned on the Typhoon Trio^+^ instrument with excitation at 532 nm and a 610-nm emission filter. Image analysis was performed as described previously by Bandow et al. (65) using Decodon Delta 2D 4.2.1 (Decodon, Greifswald, Germany). After background subtraction, the signal intensities of protein spots were normalized to the total signal on the autoradiograph and set in relation to the synthesis rate in the respective control to obtain relative synthesis rates (regulation factors) (equation 1) for individual protein spots. To be designated “marker proteins,” the relative synthesis rates had to be at least 2 in each biological replicate, and the protein had to accumulate in sufficient amounts to allow protein identification from a preparative gel.

(1) $$\text{RF}=\frac{{\text{relative signal intensity}}_{\text{antibiotic treated}}}{{\text{relative signal intensity}}_{\text{untreated control}}}$$

where RF is the regulation factor of an individual protein spot.

Proteins were identified from preparative 2D gels after tryptic in-gel digestion by either matrix-assisted laser desorption ionization–tandem time of flight (MALDI-ToF/ToF) MS (13) or nano ultraperformance liquid chromatography coupled tandem mass spectrometry with electrospray ionization (nUPLC-ESI-MS/MS) (66) as described previously, and the data were uploaded to the PRIDE repository (67).

Data from the literature were included in the downstream analysis only when experiments were performed according to the same protocols (10, 13) and satisfied the same quality standards. Thus, all data included were recorded for B. subtilis 168 grown in Belitzky minimal medium, using radioactive pulse-labeling to delineate relative synthesis rates, 2D-PAGE for protein separation, and mass spectrometry for protein identification. For an overview of treatment times, concentrations used, and the number of replicates of data gathered in this study and from the literature, see Data Set S1 in the supplemental material.

### Comparison of proteomic responses.

RFs for marker proteins (see the definition above) were determined based on the relative signal intensity on autoradiographs compared to untreated controls (equation 1). To calculate CoPR scores, the data were prepared as follows. Regulation factors of the independent replicates were averaged and logarithmized, and values for nonmarker proteins were set to zero. If literature data lacked regulation factors due to the low abundance of a protein in the control, the regulation factor was set to 20. If a protein was identified in several spots, the value in the library reflects the regulation factor of the spot with the highest percent volume representing the most intense spot with the highest protein synthesis rate. Unidentified marker proteins are omitted from the analysis.

Pairwise comparisons based on cosine similarity were performed using the regulation factors of an antibiotic a ${\text{(RF}}_{{\text{AB}}_{\text{a}}}\text{)}$ and an antibiotic b ${\text{(RF}}_{{\text{AB}}_{\text{b}}}\text{)}$, yielding a CoPR score for each pairwise comparison (equation 2). In this approach, proteomic profiles are treated as vectors, with proteins as dimensions and regulation factors as dilatation into these dimensions. A dot product is calculated, giving the sum of products of all pairwise multiplications of regulation factors of one protein, under both conditions. To give the CoPR score for the pairwise comparison, the dot product is divided by the product of the multiplication of the vector lengths. A value of 1 represents perfect similarity between response profiles, while a value of 0 represents perfect dissimilarity.

(2) $$\text{CoPR score}=\frac{\left({\text{log}}_{10}{\text{RF}}_{{\text{AB}}_{\text{a}}}\right)\cdot \left({\text{log}}_{10}{\text{RF}}_{{\text{AB}}_{\text{b}}}\right)}{\left\|{\text{log}}_{10}{\text{RF}}_{{\text{AB}}_{\text{a}}}\right\|\cdot \|{\text{log}}_{10}{\text{RF}}_{{\text{AB}}_{\text{a}}}\|}$$

where the CoPR is the comparison of proteomic responses score, ${\text{RF}}_{{\text{AB}}_{\text{a}}}$ is the regulation factor of antibiotic a, and ${\text{RF}}_{{\text{AB}}_{\text{b}}}$ is the regulation factor of antibiotic b.

Calculations were performed using Microsoft Excel and R 3.3.1 with the spatialEco package in version 1.1-0. For a step-by-step protocol, see the supplemental material.

### Data availability.

Data for protein identification by nUPLC-ESI-MS was uploaded to the PRIDE repository (project name Comparison of Proteomic Responses; project accession number PXD011640 [http://www.ebi.ac.uk/pride/archive/projects/PXD011640]). Protein regulation factors can be found in Data Set S1 in the supplemental material and the SubtiWiki repository (http://subtiwiki.uni-goettingen.de/v4/downloads). Original proteomic profiles and a step-by-step protocol on how to perform the mathematical comparison are available in the supplemental material.

## Supplementary Material

Supplemental file 1

Supplemental file 2
