# Supplementary material for: Comparison of Proteomic Responses as Global Approach to Antibiotic Mechanism of Action Elucidation
Source: Antimicrob Agents Chemother. 2020 Dec 16;65(1):e01373-20. doi: 10.1128/AAC.01373-20 (PMC7927858; doi:10.1128/AAC.01373-20)
Supplement: Supplemental file 1 [file AAC.01373-20-s0001.pdf]

Supplementary Information

Comparison of proteomic responses as global approach to antibiotic mechanism of action elucidation

Christoph H. R. Senges[1], Jennifer J. Stepanek[1], Michaela Wenzel[1,2], Nadja Raatschen[1], Ümran Ay[1], Yvonne Märtens[1], Pascal Prochnow[1], Melissa Vázquez Hernández[1], Abdulkadir Yayci[1], Britta Schubert[1], Niklas B. M. Janzing[1], Helen L. Warmuth[1], Martin Kozik[1], Jens Bongard[1], John N. Alumasa[3], Bauke Albada[4,5], Maya Penkova[4], Tadeja Lukezic[6,7], Nohemy A. Sorto[8], Nicole Lorenz[4], Reece Miller[4], Bingyao Zhu[9], Martin Benda[9], Jörg Stülke[9], Sina Schäkermann[1], Lars I. Leichert[10], Kathi Scheinpflug[11,12], Heike Brötz-Oesterhelt[13], Christian Hertweck[14], Jared T. Shaw[7], Hrvoje Petkovic[15], Jean M. Brunel[16], Kenneth C. Keiler[3], Nils Metzler-Nolte[4], Julia E. Bandow[1*]

[1]Applied Microbiology, Faculty of Biology and Biotechnology, Ruhr University Bochum, 44780 Bochum, Germany
[2]currently: Biology and Biological Engineering, Chalmers University of Technology, 412 96 Gothenburg, Sweden
[3]Biochemistry and Molecular Biology, Pennsylvania State University, PA 16802, USA
[4]Bioinorganic Chemistry, Faculty of Chemistry and Biochemistry, Ruhr University Bochum, 44780 Bochum, Germany
[5]currently: Laboratory of Organic Chemistry, Wageningen University and Research, 6708 WE Wageningen, The Netherlands
[6]ACIES BIO, Tehnoloski park 21, 1000 Ljubljana, Slovenia
[7]currently: National Institute of Biology, 1000 Ljubljana, Slovenia
[8]Department of Chemistry, University of California Davis, CA 95616, USA
[9]Department of General Microbiology, Institute of Microbiology and Genetics, Georg-August University Göttingen, 37073 Göttingen, Germany
[10]Microbial Biochemistry, Institute of Biochemistry and Pathobiochemistry, Ruhr University Bochum, 44780 Bochum, Germany
[11]Peptide Lipid Interactions, Leibniz Institute for Molecular Pharmacology, 13125 Berlin, Germany
[12]currently: Department of Wildlife Diseases, Leibniz Institute for Zoo and Wildlife Research, 10315 Berlin, Germany
[13]Microbial Bioactive Compounds, Interfaculty Institute of Microbiology and Infection Medicine Tübingen, University of Tübingen, 72076 Tübingen, Germany.
[14]Biomolecular Chemistry, Leibniz Institute for Natural Product Research and Infection Biology - Hans Knöll Institute, 07745 Jena, Germany
[15]Biotechnical Faculty, University of Ljubljana, 1001 Ljubljana, Slovenia
[16]UMR MD1, Membranes et Cibles Thérapeutiques, Faculté de pharmacie U1261 INSERM, Aix-Marseille Université, 13385 Marseille, France

***Corresponding Author**
Julia E. Bandow, julia.bandow@rub.de, +49 234 32 23102

Content:

Supplementary tables S1-71
Supplementary tables S1-40 (pages 2-29) contain protein names, functions, and regulation factors for all proteomic response profiles described newly in this work.
Supplementary tables S41-71 (pages 30-52) show data of the mass spectrometry-based identification of marker proteins from non-radioactive gels.

Supplementary figures S1-36
Supplementary figures S1-34 (pages 53-74) show growth curves, representative proteomic profiles, and the structures of the used compounds for all newly described response profiles.
Figure S35 (page 75) shows the growth curves and structures of the antibiotics, which did not elicit a proteomic response in *Bacillus subtilis* 168.
Figure S36 (page 75) shows growth of *B. subtilis* 168 and a Δ*ytrA* mutant that constitutively expresses the *ytrGABCDEF* operon, when treated with nisin.

COPR step-by-step guide
On page 76 a step-by-step guide on how to use the proteomic response library is given.

References
Pages 77 and 78.

Supplementary tables

Tab. S1 Marker proteins of acyldepsipeptide. Given are averages of regulation factors of three independent biological replicates. Protein functions and regulation are taken from the SubtiWiki database[1]. Superscripts indicate that the protein was identified in multiple spots on the gel image (see Fig. S1).

protein	regulation factor	function	regulation
AroA	2.7	biosynthesis of aromatic amino acids	
ClpC[1]	3.1	protein degradation, positive regulator of autolysin synthesis	σ^A, σ^B, σ^F, σ^M, CtsR
ClpC[2]	4.9		
ClpC[3]	8.4		
ClpC[4]	3.7		
ClpE[1]	3.3	protein degradation	σ^A, CtsR
ClpE[2]	4.6		
ClpP	3.0	protein degradation	σ^A, σ^B, CtsR
ClpY	3.9	protein degradation	σ^A, CodY
DnaK[1]	3.3	protein quality control	σ^A, HrcA
DnaK[2]	5.4		
GroEL[1]	5.7	protein folding and refolding	σ^A, HrcA
GroEL[2]	2.7		
GroEL[3]	2.1		
GroEL[4]	10.0		
PunA	3.9	purine salvage and interconversion	σ^A, CcpA
SodA	3.0	detoxification of oxygen radicals	σ^B
ThrS	3.5	translation	σ^A, T-box
TufA[1]	2.8	translation	stringent response
TufA[2]	2.5		
YfkM	4.0	detoxification of methylglyoxal	σ^B, Fur
YwfL	2.5	lipoic acid metabolism	

Tab. S2 Marker proteins of amidochelocardin 5 µg/ml. Given are averages of regulation factors of three independent biological replicates. Protein functions and regulation are taken from the SubtiWiki database[1].

protein	regulation factor	function	regulation
Abh	3.4	transcriptional regulator of transition state genes	σ^M, σ^V, σ^X
PanB	4.2	biosynthesis of coenzyme A	
YdjL	3.8	overflow metabolism; fermentation	AbrB
YwbC	3.5	detoxification of methylglyoxal	
YxxG	3.1	intercellular competition; protection against WapA	σ^A, DegU, WalR, YvrHb

Tab. S3 Marker proteins of amidochelocardin 10 µg/ml. Given are averages of regulation factors of three independent biological replicates. Protein functions and regulation are taken from the SubtiWiki database[1].

protein	regulation factor	function	regulation
Abh	5.2	transcriptional regulator of transition state genes	σ^M, σ^V, σ^X
Apt	2.6	purine salvage and interconversion	stringent response
CodY	2.5	regulation of response to branched-chain amino acid limitation	σ^A, CodY
LiaH	39.2	resistance against oxidative stress and cell wall antibiotics	σ^A, LiaR
LpdV	2.2	utilization of branched-chain keto acids	σ^L, BkdR, CodY
MccA	57.0	methionine-to-cystein conversion	σ^A, CymR, Spx
MtnB	2.5	methionine salvage	σ^A, S-box
PspA	3.6	unknown	σ^W, AbrB
RibH	3.5	riboflavin biosynthesis	σ^A, FMN-box
SufB	3.0	synthesis of iron-sulfur clusters	σ^A
TasA	3.9	biofilm formation	σ^A, AbrB, LutR, RemA, SinR
YceC	4.0	unknown	σ^B, σ^M, σ^W, σ^X
YfmS	3.8	control of chemotaxis	σ^D
YtoP	3.2	unknown	
YxxG	2.7	intercellular competition; protection against WapA	σ^A, DegU, WalR, YvrHb

Tab. S4 Marker proteins of amidochelocardin 20 µg/ml. Given are averages of regulation factors of three independent biological replicates. Protein functions and regulation are taken from the SubtiWiki database[1]. Superscripts indicate that the protein was identified in multiple spots on the gel image (see Fig. S2).

protein	regulation factor	function	regulation
Abh	3.0	transcriptional regulator of transition state genes	σ^M, σ^V, σ^X
AccB	3.2	production of malonyl-CoA	
Adk	2.7	ADP formation	σ^A, stringent response
AhpC[1]	2.6	resistance against peroxide stress	σ^A, PerR
AhpC[2]	3.9		
BkdAA	5.1	utilization of branched-chain keto acids	σ^L, BkdR, CodY
Dps	3.0	iron storage, survival of stress conditions	σ^B
GreA	2.3	transcription elongation	
LiaH[1]	667.8	resistance against oxidative stress and cell wall antibiotics	σ^A, LiaR
LiaH[2]	152.8		
LiaH[3]	37.9		
LpdV[1]	7.6	utilization of branched-chain keto acids	σ^L, BkdR, CodY
LpdV[2]	6.7		
MccA	53.7	methionine-to-cystein conversion	σ^A, CymR, Spx
PanB	9.8	biosynthesis of coenzyme A	
PdxT	2.3	pyridoxal-5-phosphate biosynthesis	Spo0A
PspA	15.6	unknown	σ^W, AbrB
RibH	5.4	riboflavin biosynthesis	σ^A, FMN-box
RsbV	2.6	control of σ^B activity	σ^A, σ^B, CcpA
SodA	2.3	detoxification of oxygen radicals	σ^B
SufB	3.7	synthesis of iron-sulfur clusters	σ^A
ThiD	7.1	biosynthesis of thiamine pyrophosphate	Thi-box
TrxA	2.8	protections of proteins against oxidative damage	σ^A, σ^B, CtsR, Spx
YceC	7.4	unknown	σ^B, σ^M, σ^W, σ^X
YceH	4.2	unknown	σ^B, σ^M, σ^W, σ^X
YdjL	22.6	overflow metabolism, fermentation	AbrB
YfmS	10.1	control of chemotaxis	σ^D
YhdN	2.8	detoxification of methylglyoxal	σ^B, FapR
YuaI	8.2	unknown	σ^W
YvlB	3.1	unknown	σ^W

Tab. S5 Marker proteins of AN3334. Given are averages of regulation factors of three independent biological replicates. Protein functions and regulation are taken from the SubtiWiki database[1].

protein	regulation factor	function	regulation
AroA	3.8	biosynthesis of aromatic amino acids	
BacB	3.8	biosynthesis of the antibiotic bacilysin	σ^A, AbrB, CodY, ScoC
GsiB	10.3	response to water deficits	σ^B, σ^I
IlvC	4.3	biosynthesis of branched-chain amino acids	σ^A, CcpA, CodY, TnrA, T-box
LeuD	13.7	biosynthesis of leucine	σ^A, CcpA, CodY, FsrA, TnrA, T-box
NfrA	3.4	unknown	σ^A, σ^D, Spo0A, Spx
OhrB	4.1	organic peroxide resistance	σ^B
PstS	3.0	high-affinity phosphate uptake	σ^A, PhoP
RocA	6.7	arginine, ornithine and citrulline utilization	σ^L, AbrB, AhrC, CodY, RocR
SpoVG	3.3	cell division; control of sporulation initiation	σ^H, AbrB, SinR
YfkM	2.8	detoxification of methylglyoxal	σ^B, Fur
YlbA	2.8	unknown	AbrB
YneT	3.4	unknown	
YsnF	2.4	survival of ethanol stress	σ^B
YtxH	3.6	unknown	σ^B, σ^H
YuaE	2.9	unknown	
YukJ	6.5	unknown	
YvyD	9.4	dimerization of ribosomes in the stationary phase, protection against paraquat stress	σ^B, σ^D, σ^H, PhoP

Tab. S6 Marker proteins of anhydrotetracycline 0.5 µg/ml. Given are averages of regulation factors of three independent biological replicates. Protein functions and regulation are taken from the SubtiWiki database[1]. Superscripts indicate that the protein was identified in multiple spots on the gel image (see Fig. S4).

protein	regulation factor	function	regulation
Abh	2.7	transcriptional regulator of transition state genes	σ^M, σ^V, σ^X
Adk[1]	3.3	ADP formation	σ^A, stringent response
Adk[2]	3.9		
GrpE	2.6	control of DnaK activity	σ^A, HrcA
HprT	2.9	purine salvage and interconversion; control of ftsH expression	σ^A, σ^F, σ^M, HprT, TolS
PanB	2.8	biosynthesis of coenzyme A	
PpiB	2.4	protein folding	σ^A
PtsH	2.8	PTS-dependent sugar transport and carbon catabolite repression	σ^A, GlcT, stringent response
PurB	2.6	purine biosynthesis	σ^A, PurR, G-Box
RplJ	2.6	translation	RplJ, RplL, stringent response
RpsF	3.5	translation	stringent response
YpuA	4.4	unknown	σ^M
YtpR	2.5	unknown	σ^A, Spx

Tab. S7 Marker proteins of anhydrotetracycline 2 µg/ml. Given are averages of regulation factors of three independent biological replicates. Protein functions and regulation are taken from the SubtiWiki database[1]. Superscripts indicate that the protein was identified in multiple spots on the gel image (see Fig. S4).

protein	regulation factor	function	regulation
Abh	5.6	transcriptional regulator of transition state genes	σ^{M}, σ^{V}, σ^{X}
Adk	3.3	ADP formation	σ^{A}, stringent response
Apt	2.8	purine salvage and interconversion	stringent response
Ctc	3.2	unknown	σ^{A}, σ^{B}, stringent response
DltA	5.9	biosynthesis of teichoic acid	σ^{D}, σ^{M}, σ^{V}, σ^{X}, Spo0A, YvrHB, stringent response
FabD	2.8	fatty acid biosynthesis	σ^{A}, ComA, FapR
FlgK	4.3	motility and chemotaxis	σ^{A}, σ^{D}, ComK, DegU, ScoC
GcaD	7.7	cell wall metabolism	σ^{A}, stringent response
GrpE	6.0	control of DnaK activity	σ^{A}, HrcA
HemE	4.6	heme biosynthesis	
HprT	2.8	purine salvage and interconversion; control of ftsH expression	σ^{A}, σ^{F}, σ^{M}, HprT, TolS
HslO	7.4	unknown	σ^{M}
LiaH[1]	105.8	resistance against oxidative stress and cell wall antibiotics	σ^{A}, LiaR
LiaH[2]	4.0		
Nfo	4.0	repair of oxidative DNA damage in spores	σ^{G}
OhrB	4.3	organic peroxide resistance	σ^{B}
PanB	3.4	biosynthesis of coenzyme A	
PanC	3.4	biosynthesis of coenzyme A	
PspA	4.8	unknown	σ^{W}, AbrB
PtsH	4.1	PTS-dependent sugar transport and carbon catabolite repression	σ^{A}, GlcT, stringent response
PurC	2.4	purine biosynthesis	σ^{A}, PurR, G-box
RplJ	3.6	translation	RplJ, RplL, stringent response
RpsF	4.5	translation	stringent response
RsbW	3.2	control of σ^{B} activity	σ^{A}, σ^{B}, CcpA
SpeB	3.4	spermidine, polyamine biosynthesis	σ^{A}
ThrC	3.7	biosynthesis of threonine	CodY, ThrR, TnrA
YceC	3.7	unknown	σ^{B}, σ^{M}, σ^{W}, σ^{X}
YceH	2.1	unknown	σ^{B}, σ^{M}, σ^{W}, σ^{X}
YdgI	4.1	unknown	
YfmS	9.0	control of chemotaxis	σ^{D}
YhcB	7.4	unknown	
YlbN	4.3	accumulation of 23S rRNA	stringent response
YojG	3.3	biosynthesis of bacillithiol	σ^{A}, Spx
YoxD	2.8	unknown	
YpuA	14.8	unknown	σ^{M}
YqiK	7.1	unknown	σ^{A}
YtpR	3.0	unknown	σ^{A}, Spx
YtsP	3.2	unknown	
YuaE	6.9	unknown	
YvlB[1]	3.1	unknown	σ^{W}
YvlB[2]	2.6		

Tab. S8 Marker proteins of anhydrotetracycline 4 µg/ml. Given are averages of regulation factors of three independent biological replicates. Protein functions and regulation are taken from the SubtiWiki database[1]. Superscripts indicate that the protein was identified in multiple spots on the gel image (see Fig. S4).

protein	regulation factor	function	regulation
Abh^1	7.4	transcriptional regulator of transition state genes	σ^M, σ^V, σ^X
Abh^2	2.9		
Apt	3.2	purine salvage and interconversion	stringent response
Ctc	5.4	unknown	σ^A, σ^B, stringent response
DltA	5.2	biosynthesis of teichoic acid	σ^D, σ^M, σ^V, σ^X, Spo0A, YvrHB, stringent response
FabD	2.7	fatty acid biosynthesis	σ^A, ComA, FapR
FlgK	5.3	motility and chemotaxis	σ^A, σ^D, ComK, DegU, ScoC
GcaD	11.1	cell wall metabolism	σ^A, stringent response
GroES	2.8		
GrpE	10.6	control of DnaK activity	σ^A, HrcA
HemE	5.5	heme biosynthesis	
HprT	2.7	purine salvage and interconversion; control of ftsH expression	σ^A, σ^F, σ^M, HprT, TilS
HslO	11.0	unknown	σ^M
LiaH^1	142.5	resistance against oxidative stress and cell wall antibiotics	σ^A, LiaR
LiaH^2	6.8		
MurAA	3.0	peptidoglycan precursor biosynthesis	σ^A, σ^F
MurF	3.3	peptidoglycan precursor biosynthesis	σ^M, σ^V
Nfo	2.7	repair of oxidative DNA damage in spores	σ^G
OhrB	5.8	organic peroxide resistance	σ^B
OpuAA	2.4	compatible solute transport	σ^A, RemA
PanC	3.3	biosynthesis of coenzyme A	
PspA	3.5	unknown	σ^W, AbrB
PtsH	5.8	PTS-dependent sugar transport and carbon catabolite repression	σ^A, GlcT, stringent response
RplJ	3.6	translation	RplJ, RplL, stringent response
RpsF	5.9	translation	stringent response
RsbW	5.0	control of σ^B activity	σ^A, σ^B, CcpA
SpeB	3.5	spermidine; polyamine biosynthesis	σ^A
SpoVG	3.0	cell division; control of sporulation initiation	σ^H, AbrB, SinR
YceC	4.2	unknown	σ^B, σ^M, σ^W, σ^X
YdgI	6.1	unknown	
YfmS	22.9	control of chemotaxis	σ^D
YhcB	7.1	unknown	
YlbN	4.2	accumulation of 23S rRNA	stringent response
YojG	2.7	biosynthesis of bacillithiol	σ^A, Spx
YoxD	2.8	unknown	
YpuA	12.5	unknown	σ^M
YqeY	2.4	unknown	
YqiK	12.6	unknown	σ^A
YtpR	3.1	unknown	σ^A, Spx
YtsP	3.8	unknwon	
YuaE	3.0	unknown	

Tab. S9 Marker Proteins of As_2O_3. Given are averages of regulation factors of three independent biological replicates. Protein functions and regulation are taken from the SubtiWiki database[1].

protein	regulation factor	function	regulation
AhpF	3.0	resistance against peroxide stress	σ^A, PerR
Ald	25.8	alanine utilization	σ^A, AdeR
AmpS	3.1	required for biofilm formation	
ArsC	26.0	detoxification of arsenate	σ^A, σ^F, ArsR
AzoR1	7.6	quinone detoxification	YodB
ClpC	4.4	protein degradation; positive regulator of autolysin synthesis	σ^A, σ^B, σ^F, σ^M, CtsR
ClpX	3.0	protein degradation	σ^A, CtsR
CysK	9.4	biosynthesis of cysteine; control of CymR activity	σ^A, σ^M, CymR, Spx
CysS	2.9	cysteine synthesis	σ^A, T-box
GroEL	6.3	protein folding and re-folding	σ^A, HrcA
Hag	3.6	motility and chemotaxis	σ^D, CodY, CsrA, ScoC
KatA	10.4	detoxification of hydrogen peroxide	PerR
Pdp	3.0	pyrimidine interconversion	σ^A, CcpA, DeoR
PspA	7.4	unknown	σ^W, AbrB
PtsI	6.3	PTS-dependent sugar transport	σ^A, GlcT, stringent response
TyrS	25.8	synthesis of tyrosyl-tRNA	σ^A, CcpA, CodY, T-box
YjdF	3.6	unknown	σ^A, MarR, RNA switch
YqcK	15.9	unknown	σ^A, σ^F, ArsR
YrhB	28.3	methionine-to-cysteine conversion	σ^A, CymR, Spx
YutF	6.6	unknown	σ^A
YvyD	16.5	dimerization of ribosomes in the stationary phase; protection against paraquat stress	σ^B, σ^D, σ^H, PhoP
YwaA	6.7	biosynthesis of branched-chain amino acids	CodY
YwfI	3.7	lipoic acid metabolism	
YxeK	2.5	utilization and detoxification of S-(2-succino)cysteine	σ^A, CymR

Tab. S10 Marker Proteins of As_2O_5. Given are averages of regulation factors of three independent biological replicates. Protein functions and regulation are taken from the SubtiWiki database[1].

protein	regulation factor	function	regulation
BkdAA	48.5	utilization of branched-chain keto acids	σ^L, BkdR, CodY
Cah	7.3	resistance to cephalosporin C	
Ctc	14.1	unknown	σ^A, σ^B, stringent response
CysK	6.2	biosynthesis of cysteine; control of CymR activity	σ^A, σ^M, CymR, Spx
Dps	7.6	iron storage, survival of stress conditions	σ^B
Eno	4.2	enzyme in glycolysis/gluconeogenesis	σ^A, CggR
Hag	3.9	motility and chemotaxis	σ^D, CodY, CsrA, ScoC
Spo0M	5.9	sporulation	σ^H, σ^W
ThrB	40.4	biosynthesis of threonine	CodY, ThrR, TnrA
YfmS	3.0	control of chemotaxis	σ^D
YhfK	5.8	unknown	
YkwC	80.7	unknown	
YtxH	6.6	unknown	σ^B, σ^H

Tab. S11 Marker proteins of ASD. Given are averages of regulation factors of three independent biological replicates. Protein functions and regulation are taken from the SubtiWiki database[1]. Superscripts indicate that the protein was identified in multiple spots on the gel image (see Fig. S7).

protein	regulation factor	function	regulation
CspR	2.5	translation	σ^E
Ctc	5.4	unknown	σ^A, σ^B, stringent response
Eno	3.7	enzyme in glycolysis/gluconeogenesis	σ^A, CggR
FabZ	4.5	fatty acid biosynthesis	
IspF	8.2	MEP pathway of isoprenoid biosynthesis	σ^B, σ^M
LiaH	8.1	resistance against oxidative stress and cell wall antibiotics	σ^A, LiaR
MsrB	3.5	regeneration of methionine and restoration of protein function after oxidative damage	σ^A, Spx
NadE	2.7	NAD biosynthesis	σ^A, σ^B
OhrB	3.8	organic peroxide resistance	σ^B
PepF	3.9	protein degradation	
Pgm	2.4	enzyme in glycolysis/gluconeogenesis	σ^A, CggR
PrmA	4.6	unknown	σ^A, HrcA
PspA	10.1	unknown	σ^W, AbrB
PtsH	4.2	PTS-dependent sugar transport and carbon catabolite repression	σ^A, GlcT, stringent response
RacX	5.1	production of non-canonical D-amino acids	σ^W
RibH	3.8	riboflavin synthesis	σ^A, FMN-box
RimM	2.9	maturation of 16S rRNA	
RplL^1	4.5	translation	RplL, stringent response
RplL^2	3.2		
RplS	2.9	translation	stringent response
Sat	2.6	sulfate activation	σ^A, CymR, S-box
SdhA	6.4	TCA cycle	σ^A, FarA
SigX	3.9	resistance to cationic antimicrobial peptides	σ^A, σ^X, YvrHb
ThrC	2.6	biosynthesis of threonine	CodY, ThrR, TnrA
TrmK	4.7	tRNA modification	
YceC	5.3	unknown	σ^B, σ^M, σ^W, σ^X
YfmS	8.3	control of chemotaxis	σ^D
YjdA	4.5	unknown	
YjoA	3.0	unknown	
YphP	3.1	de-bacillithiolation of S-bacillithiolated OhrR and MetE	
YqhS	2.3	unknown	
YqiG	2.6	unknown	
YqkB	3.4	unknown	
YqkF	8.2	unknown	σ^E
YsnF	4.4	survival of ethanol stress	σ^B
YutE	4.0	unknown	σ^A
YvlB	4.8	unknown	σ^W
YwrO	3.9	unknown	

Tab. S12 Marker proteins of auranofin. Given are averages of regulation factors of three independent biological replicates. Protein functions and regulation are taken from the SubtiWiki database[1]. Superscripts indicate that the protein was identified in multiple spots on the gel image (see Fig. S8).

protein	regulation factor	function	regulation
ArsC	10.7	detoxification of arsenate	$σ^A$, $σ^F$, ArsR
Cah	13.3	resistance to cephalosporin C	
CitB	18.5	TCA cycle	$σ^A$, CcpA, CcpC, CodY, FsrA
CitZ	5.9	TCA cycle	$σ^A$, CcpA, CcpC, CitB
Hag	2.8	motility and chemotaxis	$σ^D$, CodY, CsrA, ScoC
KatA[1]	18.1	detoxification of hydrogen peroxide	PerR
KatA[2]	6.9		
KatA[3]	6.5		
KatA[4]	5.5		
MrgA	8.4	iron storage	$σ^A$, PerR
YpfD	8.4	unknown	
YrhB[1]	12.2	methionine-to-cystein conversion	$σ^A$, CymR, Spx
YrhB[2]	3.5		
YuaE	3.4	unknown	
YwkD	4.6	unknown	
YxeP	3.5	utilization and detoxification of S-(2-succino)cysteine	$σ^A$, CymR

Tab. S13 Marker proteins BA234. Given are averages of regulation factors of three independent biological replicates. Protein functions and regulation are taken from the SubtiWiki database[1]. Superscripts indicate that the protein was identified in multiple spots on the gel image (see Fig. S9).

protein	regulation factor	function	regulation
AcsA[1]	6.7	utilization of acetate, fatty acids	σ^A, CcpA, CodY
AcsA[2]	9.4		
AtpA	7.7	ATP synthesis	stringent response
CitZ	4.2	TCA cycle	σ^A, CcpA, CcpC, CitB
ClpP	4.3	protein degradation	σ^A, σ^B, CtsR
DisA	3.9	control of sporulation initiation	σ^A, σ^B, σ^F, σ^W, CtsR
FabG	6.9	fatty acid biosynthesis	σ^A, ComA, FapR
FbaA	3.7	enzyme in glycolysis/gluconeogenesis	
GsiB	3.1	response to water deficits	σ^B, σ^I
HemH	3.8	biosynthesis of heme	
IolD	4.5	myo-inositol catabolism	σ^A, CcpA, IolR
LiaH[1]	42.9	resistance against oxidative stress and cell wall antibiotics	σ^A, LiaR
LiaH[2]	33.3		
LiaH[3]	6.7		
LiaR	5.6	regulation of the *liaI-liaH-liaG-liaF-liaS-liaR* operon	σ^A, LiaR
LpdV	3.4	utilization of branched-chain keto acids	σ^L, CodY, BkdR
MlpA	9.3	control of proteolytic activity	
PbpE	9.9	endopeptidase	σ^W
PspA[1]	19.0	unknown	σ^W, AbrB
PspA[2]	18.6		
PspA[3]	12.2		
SalA	3.6	control of alkaline protease expression	SalA
SpeE	3.7	spermidine/polyamine biosynthesis	σ^A
TcyA	7.1	cystein uptake	
TrxB	3.6	keeps thioredoxin in the reduced state	Spx
YaaQ	3.4	unknown	
YbfO	24.5	unknown	σ^W, AbrB
YceC	6.9	unknown	σ^B, σ^M, σ^W, σ^X
YceH	7.7	unknown	σ^B, σ^M, σ^W, σ^X
YdaG	3.2	survival of stress conditions	σ^B
YdbD	4.0	survival of ethanol stress	σ^B
YdgI	4.2	unknown	
YfhM	11.0	survival of ethanol stress	σ^B, σ^W
YhdN	10.2	detoxification of methylglyoxal	σ^B, FapR
YhfE	2.8	unknown	
YhfK	2.5	unknown	
YknY	6.7	resistance to SdpC toxin	σ^W, AbrB
YqiG	3.7	unknown	
YsnF	2.7	survival of ethanol stress	σ^B
YuaE	3.5	unknown	
YuaI	3.7	unknown	σ^W
YugJ	5.7	unknown	Spx
YvgN	3.7	unknown	σ^B, SinR
YvlB[1]	3.6	unknown	σ^W
YvlB[2]	2.9		
YwfI	6.1	lipoic acid metabolism	
YwrO	3.0	unknown	

Tab. S14 Marker proteins of CCCP. Part 1 of 2. Given are averages of regulation factors of three independent biological replicates. Protein functions and regulation are taken from the SubtiWiki database[1]. Superscripts indicate that the protein was identified in multiple spots on the gel image (see Fig. S10).

protein	regulation factor	function	regulation
AcuA	4.0	control of AcsA activity	σ^A, CcpA
Ald	7.5	alanine utilization	σ^A, AdeR
BglA	6.0	beta-glucoside utilization	
BglH	6.6	salicin utilization	σ^A, CcpA, LicT
Ctc	5.1	unknown	σ^A, σ^B, stringent response
Def	8.1	translation	σ^A
Dps	3.8	iron storage; survival of stress conditions	σ^B
EtfA	6.8	fatty acid degradation; calcium carbonate biomineralization	σ^A, CcpA, FadR
EtfB	3.0	fatty acid degradation	σ^A, CcpA, FadR
FadR	3.8	regulation of fatty acid degradation	σ^A, CcpA, FadR
FrlB	4.8	metabolism of aminoacylated fructose	σ^A, CodY, FrlR
GsiB	5.7	response to water deficits	σ^B, σ^I
HisA	7.3	biosynthesis of histidine	
HisB	7.8	biosynthesis of histidine	
HisD	8.9	biosynthesis of histidine	
HisF	9.4	biosynthesis of histidine	
HisG	6.0	biosynthesis of histidine	
HisH[1]	6.5	biosynthesis of histidine	
HisH[2]	7.1		
HisI	8.5	biosynthesis of histidine	
LpdV	6.6	utilization of branched-chain keto acids	σ^L, BkdR, CodY
MelA	13.6	melibiose utilization	σ^A, CcpA
MetN	12.7	methionine uptake	CodY, S-box
MurF	5.4	peptidoglycan precurser biosynthesis	σ^M, σ^V
NagBB	3.1	N-acetylglucosamine utilization	σ^A, CcpA, NagR
NfrA	3.1	unknown	σ^A, σ^D, Spo0A, Spx
OhrB	3.5	organic peroxide resistance	σ^B
RocA[1]	9.2	arginine, ornithine and citrulline utilization	σ^L, AbrB, AhrC, CodY, RocR
RocA[2]	7.8		
RpsW	6.0	translation	
SacA	5.4	utilization of sucrose	σ^A, CcpA, SacT
Tsf	7.5	translation	stringent response
TyrA	4.8	biosynthesis of tyrosine	MtrB

Tab. S15 Marker proteins of CCCP. Part 2 of 2. Given are averages of regulation factors of three independent biological replicates. Protein functions and regulation are taken from the SubtiWiki database[1]. Superscripts indicate that the protein was identified in multiple spots on the gel image (see Fig. S10).

protein	regulation factor	function	regulation
YaaQ	6.3	unknown	
YcsN	3.8	unknown	
YdaE	4.5	survival of ethanol stress and low temperatures	
YdbD[1]	3.0	survival of ethanol stress	σ^B
YdbD[2]	7.6		
YerA	15.3	unknown	
YezE	4.2	unknown	
YfkM	3.2	detoxification of methylglyoxal	σ^B, Fur
YhdF	6.9	unknown	σ^A, σ^B, CitR
YhdN[1]	4.6	detoxification of methylglyoxal	σ^B, FapR
YhdN[2]	8.0		
YjbC	2.3	survival of paraquat stress	σ^B, σ^M, σ^W, σ^X, PerR
YocK	4.1	survival of ethanol stress	σ^B
YpfD	3.2	unknown	
YrbE	8.0	unknown	
YsiB	11.6	fatty acid degradation	σ^A, CcpA, FadR
YsnF	6.9	survival of ethanol stress	σ^B
YtxH	4.6	unknown	σ^B, σ^H
YukJ	6.0	unknown	
YvyD	14.5	dimerization of ribosomes in the stationary phase; protection aganst paraquat stress	σ^B, σ^D, σ^H, PhoP
YxaB	4.6	biofilm formation; survival of salt and ethanol stress	σ^A, σ^B, AbrB
YxbG	12.7	unknown	σ^B

Tab. S16 Marker proteins of cinnamaldehyde. Given are averages of regulation factors of three independent biological replicates. Protein functions and regulation are taken from the SubtiWiki database[1]. Superscripts indicate that the protein was identified in multiple spots on the gel image (see Fig. S11).

protein	regulation factor	function	regulation
Cah	11.3	resistance to cephalosporin C	
ClpP	7.9	protein degradation	σ^A, σ^B, CtsR
DnaK	7.6	protein quality control	σ^A, HrcA
FabG	3.0	fatty acid biosynthesis	σ^A, ComA, FapR
IolS	4.5	unknown; may be involved in myo-inositol catabolism	σ^A, IolR
LeuD	19.6	biosynthesis of leucine	σ^A, CcpA, Cody, FsrA, TnrA, T-box
NfrA	4.6	unknown	σ^A, σ^D, Spo0A, Spx
PbpC[1]	8.8	unknown	
PbpC[2]	13.3		
PtsL	6.4	PTS-dependent sugar transport	σ^A, Glct, stringent response
SalA	8.2	control of alkaline protease expression	SalA
SodA	3.9	detoxification of oxygen radicals	σ^B
SuhB	12.3	unknown	σ^B
ThrS[1]	11.9	translation	σ^A, T-box
ThrS[2]	18.1		
YaaE	3.9	pyridoxal-5-phosphate biosynthesis	Spo0A
YjdA	4.5	unknown	
YqiG	9.1	unknown	
YugJ	5.6	unknown	Spx

Tab. S17 Marker proteins of ciprofloxacin. Given are averages of regulation factors of three independent biological replicates. Protein functions and regulation are taken from the SubtiWiki database[1].

protein	regulation factor	function	regulation
Apt	5.4	purine salvage and interconversion	stringent response
Ctc	7.9	unknown	$σ^A$, $σ^B$, stringent response
DinB	12.4	response to DNA damage	LexA
DnaC	2.5	DNA replication	
EfeM	9.5	elemental iron uptake	$σ^A$, $σ^M$, $σ^W$, $σ^X$, Fur
Fur	5.0	regulation of iron homeostasis	$σ^A$, PerR
GreA	6.0	transcription elongation	
GspA	4.5	unknown	$σ^B$
GtaB	2.8	biosynthesis of teichoic acid	$σ^A$, $σ^B$
OhrB	3.3	organic peroxide resistance	$σ^B$
Pgm	2.3	enzyme in glycolysis/gluconeogenesis	$σ^A$, CggR
PurH	2.2	purine biosynthesis	$σ^A$, PurR, G-box
RsbW	4.2	control of $σ^B$ activity	$σ^A$, $σ^B$, CcpA
ThiE	2.9	biosynthesis of thiamine by 4-methyl-5-5(beta-hydroxyethyl)thiazole phosphate to yield thiamine phosphate	$σ^A$
YceD	46.2	required for survival of ethanol stress	$σ^B$, $σ^M$, $σ^W$, $σ^X$
YdaD	6.0	unknown	$σ^B$
YdaG	5.0	survival of stress conditions	$σ^B$
YeeI	4.6	unknown	
YfhH	2.7	unknown	
YlbN	3.9	accumulation of 23S rRNA	stringent response
YsnA	2.8	purine metabolism	
YtxH	4.1	unknown	$σ^B$, $σ^H$
YtzG	2.6	16s rRNA modification	
YugI	3.1	unknown	stringent response
YugK	14.8	unknown	
YvaK	2.3	unknown	$σ^B$
YvyD	7.8	dimerization of ribosomes in the stationary phase; protection against paraquat stress	$σ^B$, $σ^D$, $σ^H$, PhoP
YwqK	5.3	unknown	

Tab. S18 Marker proteins of closthioamide. Given are averages of regulation factors of three independent biological replicates. Protein functions and regulation are taken from the SubtiWiki database[1]. Superscripts indicate that the protein was identified in multiple spots on the gel image (see Fig. S13).

protein	regulation factor	function	regulation
ArgS	5.5	translation	
AzoR2	5.0	resistance to 2-methylhydroquinone	σ^A, σ^G, MhqR
GbsB[1]	5.9	osmoprotection	σ^A, GbsR
GbsB[2]	3.9		
GlpX	2.3	gluconeogenesis	
GltX	4.9	translation	σ^A, T-box
GuaA	5.4	biosynthesis of GMP	σ^A
IlvE	9.9	biosynthesis of branched-chain amino acids	CodY
Maf	4.2	cell division control	σ^A, σ^M, σ^V, ComK
MenB	3.8	biosynthesis of menaquinone	σ^A
MhqO	4.0	resistance to methyl-hydroxyquinone	σ^A, σ^B, MhqR
MnaA	3.0	biosynthesis of teichoic acid	
MrgA	11.0	iron storage	σ^A, PerR
MtnK	2.9	methionine salvage	σ^A, S-box
MurD	3.8	peptidoglycan precursor biosynthesis	σ^E, SpoIIID, stringent response
MurQ	5.8	cell wall turnover	
NdoA	3.1	unknown	
OhrA	8.6	organic peroxide resistance	σ^A, OhrR
PatA	2.7	biosynthesis of lysine and peptidoglycan	
PckA	5.2	synthesis of phosphoenolpyruvate	σ^A, CcpN
ProS	9.9	translation	
PurA	5.2	purine biosynthesis	σ^A, PurR
PurM	3.5	purine biosynthesis	σ^A, PurR, G-Box
RapG	4.5	control of DegU activity	σ^A, σ^H, CcpA, RghR, SinR
RibE	17.3	riboflavin biosynthesis	σ^A, FMN-box
ThiE	2.6	biosynthesis of thiamine by 4-methyl-5-5(beta-hydroxyethyl)thiazole phosphate to yield thiamine phosphate	σ^A
ThiQ	3.1	unknown	σ^E, SpoIIID
YcnD	10.2	delivery of FMN to enzymes	
YcnE	8.4	unknown	
YdgI	2.8	unknown	
YsnF	3.4	survival of ethanol stress	σ^B
YhdN	5.1	detoxification of methylglyoxal	σ^B, FapR
YopQ	3.8	unknown	
YopX	19.0	unknown	
YorG	6.6	unknown	
YtsJ	4.4	malate utilization	LexA
YtxJ	5.5	unknown	σ^B, σ^H
YxjG	3.9	unknown	S-box

Tab. S19 Marker proteins of cXRX. Given are averages of regulation factors of three independent biological replicates. Protein functions and regulation are taken from the SubtiWiki database[1].

protein	regulation factor	function	regulation
CitZ	2.6	TCA cycle	σ^A, CcpA, CcpC, CitB
ClpC	2.6	protein degradation; positive regulator of autolysin synthesis	σ^A, σ^B, σ^F, σ^M, CtsR
ClpP	3.4	protein degradation	σ^A, σ^B, CtsR
DltA	3.6	biosynthesis of teichoic acid	σ^D, σ^M, σ^V, σ^X, Spo0A, YvrHB, stringent response
FosB	3.8	confers resistance against antimicrobial compounds form *B. amyloliquefaciens*	σ^W
GlpK	2.5	glycerol utilization	σ^A, AbrB, CcpA, GlpP, ResD
GroEL	3.9	protein folding and re-folding	σ^A, HrcA
GsiB	5.1	response to water deficits	σ^B, σ^I
NadE	4.4	NAD biosynthesis	σ^A, σ^B
PspA	3.9	unknown	σ^W, AbrB
RacX	2.8	production of non-canonical D-amino acids	σ^W
Tpx	3.2	unknown	Spx
YceC	3.0	unknown	σ^B, σ^M, σ^W, σ^X
YdbD	2.6	survival of ethanol stress	σ^B
YjdA	2.6	unknown	
YoxD	3.0	unknown	
YpuA	4.0	unknown	σ^M
YvlB	3.2	unknown	σ^W
YxaB	4.2	biofilm formation; survival of salt and ethanol stress	σ^A, σ^B, AbrB

Tab. S20 Marker proteins of 5% DMSO. Given are averages of regulation factors of three independent biological replicates. Protein functions and regulation are taken from the SubtiWiki database[1]. Superscripts indicate that the protein was identified in multiple spots on the gel image (see Fig. S15).

protein	regulation factor	function	regulation
Adk	12.7	ADP formation	σ^A, stringent response
ClpC	3.2	protein degradation; positive regulator of autolysin synthesis	σ^A, σ^B, σ^F, σ^M, CtsR
ClpP	2.7	protein degradation	σ^A, σ^B, CtsR
DapA[1]	10.0	biosynthesis of lysine and peptidoglycan	σ^A, σ^{KN}
DapA[2]	21.0		
Dps[1]	3.9	iron storage; survival of stress conditions	σ^B
Dps[2]	6.0		
Dps[3]	6.9		
FbaA[1]	4.9	enzyme in glycolysis/gluconeogenesis	
FbaA[2]	4.3		
FbaA[3]	16.5		
GreA	6.3	transcription elongation	
GrpE	2.5	control of DnaK activity	σ^A, HrcA
GtaB[1]	51.0	biosynthesis of teichoic acid	σ^A, σ^B
GtaB[2]	21.0		
GtaB[3]	16.5		
IleS	3.8	translation	T-box
IlvD	18.0	biosynthesis of branched-chain amino acids	CodY
KatE	8.8	detoxification of hydrogen peroxide	σ^B
MalS	11.2	malate utilization	
MetE	6.5	biosynthesis of methionine	S-box
OhrB	11.9	organic peroxide resistance	σ^B
RpsC	7.4	translation	σ^A, stringent response
RsbW	4.0	control of σ^B activity	σ^A, σ^B, CcpA
Sat	20.0	sulfate activation	σ^A, CymR, S-box
ThiD	21.0	biosynthesis of thiamine pyrophosphate	Thi-box
ThyA	4.9	biosynthesis of thymidine nucleotides	
YcdF	8.6	survival of ethanol stress and at low temperatures	σ^B
YceE	12.7	survival of ethanol stress and at low temperatures	σ^B, σ^M, σ^W, σ^X
YdaD	5.7	unknown	
YdaE	29.3	survival of ethanol stress and at low temperatures	σ^B
YeeI	4.7	unknown	
YfkM[1]	9.4	detoxification of methylglyoxal	σ^B, Fur
YfkM[2]	7.0		
YhdF	22.3	unknown	σ^A, σ^B, CitR
YloC	21.0	unknown	σ^F
YpfD[1]	12.4	unknown	
YpfD[2]	16.7		
YsnF[1]	20.4	survival of ethanol stress	σ^B
YsnF[2]	20.0		
YsnF[3]	28.8		
YsnF[4]	25.2		
YtxH	5.4	unknown	σ^B, σ^H
YvgN	16.5	unknown	σ^B, SinR
YvrE	5.8	survival of salt stress	σ^B
YxaB[1]	18.2	biofilm formation; surival of salt and ethanol stress	σ^A, σ^B, AbrB
YxaB[2]	12.4		

Tab. S21 Marker proteins of GE2270A. Given are averages of regulation factors of three independent biological replicates. Protein functions and regulation are taken from the SubtiWiki database[1].

protein	regulation factor	function	regulation
CheV	2.7	control of CheA activity	σ^D
Nfo	2.2	repair of oxidative DNA damage in spores	σ^G
QueF	2.5	tRNA modification	preQ1 riboswitch

Tab. S22 Marker proteins of kirromycin. Given are averages of regulation factors of three independent biological replicates. Protein functions and regulation are taken from the SubtiWiki database[1]. Superscripts indicate that the protein was identified in multiple spots on the gel image (see Fig. S17).

protein	regulation factor	function	regulation
ClpC[1]	40.6	protein degradation, positive regulator of autolysin synthesis	σ^A, σ^B, σ^F, σ^M, CtsR
ClpC[2]	8.6		
ClpP	3.4	protein degradation	σ^A, σ^B, CtsR
DnaK	2.4	protein quality control	σ^A, HrcA
GrpE	2.9	control of DnaK activity	σ^A, HrcA
GuaC	21.5	purine salvage and interconversion	CodY, PurR
HemH	2.4	biosynthesis of heme	
PyrAB	3.4	pyrimidine biosynthesis	σ^A, PyrR
YdgI	3.0	unknown	
YhcB	22.6	unknown	
YojG	2.3	biosynthesis of bacillithiol	σ^A, Spx
YvlB	2.1	unknown	σ^W

Tab. S23 Marker proteins of kirrothricin C. Given are averages of regulation factors of three independent biological replicates. Protein functions and regulation are taken from the SubtiWiki database[1].

protein	regulation factor	function	regulation
ClpC	11.5	protein degradation, positive regulator of autolysin synthesis	σ^A, σ^B, σ^F, σ^M, CtsR
DnaK	2.0	protein quality control	σ^A, HrcA
GroEL	4.8	protein folding and re-folding	σ^A, HrcA
GroES	5.3	protein folding and re-folding	σ^A, HrcA
GsiB	13.7	response to water deficits	σ^B, σ^I
GtaB	11.6	biosynthesis of teichoic acid	σ^A, σ^B
PtsH	11.8	PTS-dependent sugar transport and carbon catabolite repression	σ^A, GlcT, stringent response
TrxA	4.4	protection of proteins against oxidative damage	σ^A, σ^B, CtsR, Spx
YdgI	20.0	unknown	
YuaE	20.0	unknown	

Tab. S24 Marker proteins of KKL-35. Given are averages of regulation factors of three independent biological replicates. Protein functions and regulation are taken from the SubtiWiki database[1]. Superscripts indicate that the protein was identified in multiple spots on the gel image (see Fig. S19).

protein	regulation factor	function	regulation
AhpC[1]	5.9	resistance against peroxide stress	σ^A, PerR
AhpC[2]	8.1		
AhpF[1]	8.8	resistance against peroxide stress	σ^A, PerR
AhpF[2]	8.8		
AhpF[3]	5.5		
AhpF[4]	4.8		
BrxA	19.1	de-bacillithiolation of S-bacillithiolated OhrR and MetE	
DhbA	14.1	biosynthesis of the siderophore bacillibactin	σ^A, AbrB, Fur, Kre
Fur	4.0	regulation of iron homeostasis	σ^A, Fur, PerR
GuaB	3.5	biosynthesis of GMP	CodY
HisA	12.1	biosynthesis of histidine	
HisG	5.3	biosynthesis of histidine	
IscU	23.1	transfer of sulfur from SufS to SufB	σ^A
KatA[1]	87.8	detoxification (degradation) of hydrogen peroxide	PerR
KatA[2]	24.1		
KatA[3]	17.1		
MrgA	14.3	iron storage	σ^A, PerR
NfrA	3.8	unknown	σ^A, σ^D, Spo0A, Spx
SufD	2.6	synthesis of Fe-S-clusters	σ^A
Tpx	7.1	unknown	Spx
TrxA	5.1	protection of proteins against oxidative damage	σ^A, σ^B, CtsR, Spx
YcsN	3.7	unknown	
YhzC	195.0	unknown	
YukJ	4.9	unknown	
YvyD	9.3	dimerization of ribosomes in the stationary phase; protection against paraquat stress	σ^B, σ^D, σ^H, PhoP
YwrO	2.8	unknown	

Tab. S25 Marker proteins of KKL-40. Given are averages of regulation factors of three independent biological replicates. Protein functionsand regulation are taken from the SubtiWiki database[1]. Superscripts indicate that the protein was identified in multiple spots on the gel image (see Fig. S20).

protein	regulation factor	function	regulation
AhpC[1]	5.8	resistance against peroxide stress	σ^A, PerR
AhpC[2]	9.9		
AhpF[1]	7.1	resistance against peroxide stress	σ^A, PerR
AhpF[2]	7.0		
AhpF[3]	5.0		
BrxA	20.6	de-bacillithiolation of S-bacillithiolated OhrR and MetE	
Ctc	3.6	unknown	σ^A, σ^B, stringent response
GreA	7.0	transcription elongation	
HisA	10.5	biosynthesis of histidine	
HisG	5.9	biosynthesis of histidine	
IscU	26.3	transfer of sulfur from SufS to SufB	
MrgA	22.5	iron storage	σ^A, PerR
NfrA	3.2	unknown	σ^A, σ^D, Spo0A, Spx
OhrB	3.6	organic peroxide resistance	σ^A
TrxA	5.6	protection of proteins against oxidative damage	σ^A, σ^B, CtsR, Spx
YfkM	2.4	detoxification of methylglyoxal	σ^B, Fur
YhzC	170.2	unknown	
YtpR	5.2	unknown	σ^A, Spx
YtxH	14.2	unknown	σ^B, σ^H
YvyD	12.6	dimerization of ribosomes in the stationary phase; protection against paraquat stress	σ^B, σ^D, σ^H, PhoP

Tab. S26 Marker proteins of KKL-55. Given are averages of regulation factors of three independent biological replicates. Protein functions and regulation are taken from the SubtiWiki database[1]. Superscripts indicate that the protein was identified in multiple spots on the gel image (see Fig. S21).

protein	regulation factor	function	regulation
AhpC	4.8	resistance against peroxide stress	σ^A, PerR
AhpF	4.2	resistance against peroxide stress	σ^A, PerR
AzoR1	4.3	quinone detoxification	YodB
ClpP	5.0	proteind egradation	σ^A, σ^B, CtsR
DhbA	4.8	biosynthesis of the siderophore bacillibactin	σ^A, σ^I, AbrB, Fur, Kre
DhbB	14.2	biosynthesis of the siderophore bacillibactin	σ^A, σ^I, AbrB, Fur, Kre
FlgK	6.2	motility and chemotaxis	σ^A, σ^D, ComK, DegU, ScoC
GroEL[1]	4.2	protein folding and re-folding	σ^A, HrcA
GroEL[2]	7.4		
KatA	37.0	getoxification of hydrogen peroxide	PerR
NagB	4.8	N-acetylglucosamine utilization	σ^A, CcpA, NagR
PepF	3.1	protein degradation	
TenA	2.6	thiamien salvage	Thi-box
YcnD	6.0	delivery of FMN to enzymes	
YhjG	5.1	unknown	
YneT	99.8	unknown	
YqiG	6.7	unknown	
YugJ	4.7	unknown	Spx
YwrO	2.9	unknown	

Tab. S27 Marker proteins of linezolid. Given are averages of regulation factors of three independent biological replicates. Protein functions and regulation are taken from the SubtiWiki database[1].

protein	regulation factor	function	regulation
Adk	7.0	ADP formation	σ^A, stringent response
CcpA	2.8	carbon catabolite repression (CCR)	σ^A
CodY	13.1	regulation of a large regulon in response to branched-chain amino acid limitation	σ^A, CodY
CspR	3.3	translation	σ^E
CwlO	3.5	cell wall synthesis; cell elongation	σ^A, WalR
Fur	4.1	regulation of iron homeostasis	σ^A, PerR
HslO	7.0	unknown	σ^M
IolS	3.4	unknown; maybe involved in myo-inositol catabolism	σ^A, IolR
MnaA	3.6	biosynthesis of teichoic acid	
NusG	3.4	transcription	
PrfB	7.8	translation	σ^A
PurC	13.1	purine biosynthesis	σ^A, PurR, G-box
PyrB	2.5	pyrimidine biosynthesis	σ^A, PyrR
PyrC	3.4	pyrimidine biosynthesis	σ^A, PyrR
RplJ	3.7	translation	RplJ, RplL, stringent response
RpsF[1]	2.9	translation	σ^A, ComK, stringent response
RpsF[2]	3.3		
SpeB	3.0	spermidine; polyamine biosynthesis	σ^A
SsbA	5.0	DNA replication, DNA repair/recombination	σ^A, ComK, stringent response
SucD	3.1	TCA cycle	CcpA
TagD	3.0	biosynthesis of teichoic acid	σ^A, PhoP, WalR
TasA	2.6	biofilm formation	σ^A, AbrB, LutR, RemA, SinR
TrmK	5.4	tRNA modification	
TrxB	3.2	keeps TrxA in reduced state	Spx
YceE	2.2	required for survival of ethanol stress and at low temperatures	σ^B, σ^M, σ^W, σ^X
YfmS	3.0	control of chemotaxis	σ^D
YfmT	2.9	unknown	σ^D
YjcJ	2.6	biosynthesis of methionine	σ^A, S-box
YlbN	2.4	accumulation of 23S rRNA	stringent response
YocH	2.4	cell wall turnover	σ^A, AbrB, Spo0A, WalR
YutI	4.6	unknown	
YwtE	6.0	dephosphorylation of riboflavin precursor; 5-amino-6-ribitylamino-2,3(1H,3)-pyrimidinedione 5'-phosphate	

Tab. S28 Marker proteins of monensin. Given are averages of regulation factors of three independent biological replicates. Protein functions and regulation are taken from the SubtiWiki database[1].

protein	regulation factor	function	regulation
AspS	2.2	translation	T-box
CysK	3.8	biosynthesis of cysteine; control of CymR activity	σ^A, CymR, Spx
FolD	5.7	formylation of Met-tRNA	PurR, stringent response
GuaB	3.2	biosynthesis of GMP	CodY
GudB	4.4	glutamate utilization; control of GltC activity	σ^A
HemK	2.8	heme biosynthesis	σ^A, PerR
HisF	4.6	biosynthesis of histidine	
HisI	5.0	biosynthesis of histidine	
HisZ	5.3	translation	
IlvD	3.7	biosynthesis of branched-chain amino acids	CodY
KtrC	6.6	potassium uptake	σ^A, Spo0A
LuxS	2.8	methionine salvage	σ^B
MetC	5.0	biosynthesis of methionine	σ^A, S-box
PatA	4.1	biosynthesis of lysine and peptidoglycan	
SpoVC	2.9	spore coat formation	σ^A, σ^B, stringent response
TyrZ	3.3	translation	
YaaD	3.9	pyridoxal-5-phosphate biosynthesis	Spo0A
YceC	2.9	unknown	σ^B, σ^M, σ^W, σ^X
YceH	2.6	unknown	σ^B, σ^M, σ^W, σ^X
YdjE	4.5	utilization of sucrose and glucitol	
YfmS	3.5	control of chemotaxis	σ^D
YhcB	5.8	unknown	
YjbL	3.3	unknown	
YkuU	4.3	protection against peroxide stress	AbrB
YqeY	4.9	unknown	
YqhK	3.1	glycine utilization	Gly-box
YtiB	2.5	unknown	σ^B
YuaE	5.6	unknown	
YwfI	3.7	biosynthesis of heme	
YwjH	3.7	pentose phosphate pathway	
YwlF	6.6	pentose phosphate pathway	TnrA

Tab. S29 Marker proteins of L-MP66. Given are averages of regulation factors of three independent biological replicates. Protein functions and regulation are taken from the SubtiWiki database[1].

protein	regulation factor	function	regulation
AzoR2	7.5	resistance to 2-methylhydroquinone	σ^A, σ^G, MhqR
DltA	3.5	biosynthesis of teichoic acid	σ^D, σ^M, σ^X, σ^V, Spo0A, YvrHb, stringent response
FabF	4.0	fatty acid biosynthesis	σ^A, σ^W, FapR
GsiB	2.4	response to water deficits	σ^B, σ^I
LiaH	72.2	resistance against oxidative stress and cell wall antibiotics	σ^A, LiaR
NadE	4.0	NAD biosynthesis	σ^A, σ^B
NfrA	5.3	unknown	σ^A, σ^D, Spo0A, Spx
PspA	7.3	unknown	σ^W, AbrB
Spo0M	3.2	sporulation	σ^H, σ^W
TufA	3.3	translation	stringent response
YceC	6.6	unknown	σ^B, σ^M, σ^W, σ^X
YhdN	2.4	detoxification of methylglyoxal	σ^B, FapR
YjdA	2.8	unknown	
YoxD	2.7	unknown	
YqiW	3.2	de-bacillithiolation of S-bacillithiolated OhrR and MetE	
YqkF	2.8	unknown	σ^E
YuaI	2.7	unknown	σ^W
YwrO	2.8	unknown	

Tab. S30 Marker proteins of D-MP66. Given are averages of regulation factors of three independent biological replicates. Protein functions and regulation are taken from the SubtiWiki database[1].

protein	regulation factor	function	regulation
ClpP	4.3	protein degradation	σ^A, σ^B, CtsR
DltA	2.5	biosynthesis of teichoic acid	σ^D, σ^M, σ^X, σ^V, Spo0A, YvrHb, stringent response
Dps	7.6	iron storage; survival of stress conditions	σ^B
GreA	4.2	transcription elongation	
GsiB	3.7	response to water deficits	σ^I, σ^B
LiaH	44.1	resistance against oxidative stress and cell wall antibiotics	σ^A, LiaR
NadE	9.2	NAD biosynthesis	σ^A, σ^B
NfrA	5.1	unknown	σ^A, σ^D, Spo0A, Spx
PbpE	3.5	endopeptidase	σ^W
PspA	9.1	unknown	σ^W, AbrB
RacX	7.1	production of non-canonical D-amino acids	σ^W
SalA	3.1	control of alkaline protease expression	SalA
Spo0M	6.3	sporulation	σ^H, σ^W
YceC	16.7	unknown	σ^A, σ^M, σ^W, σ^X
YdaG	12.4	survival of stress conditions	σ^B
YdbD	12.3	survival of ethanol stress	σ^B
YjdA	18.5	unknown	
YoxD	9.3	unknown	
YqkF	2.0	unknown	σ^E
YtkL	2.2	unknown	σ^B

Tab. S31 Marker proteins of L-MP159. Given are averages of regulation factors of three independent biological replicates. Protein functions and regulation are taken from the SubtiWiki database[1].

protein	regulation factor	function	regulation
AzoR2	8.4	resistance to 2-methylhydroquinone	σ^A, σ^G, MhqR
CitZ	5.2	TCA cycle	σ^A, CcpA, CcpC, CitB
ClpP	3.5	protein degradation	σ^A, σ^B, CtsR
Dps	2.0	iron storage; survival of stress conditions	σ^B
LiaH	27.9	resistance against oxidative stress and cell wall antibiotics	σ^A, LiaR
LuxS	2.4	methionine salvage	σ^B
MrgA	6.1	iron storage	σ^A, PerR
NadE	10.8	NAD biosynthesis	σ^A, σ^B
NfrA	4.7	unknown	σ^A, σ^D, Spo0A, Spx
PbpE	4.7	endopeptidase	σ^W
PspA	6.2	unknown	σ^W, AbrB
RacX	10.5	production of non-canonical D-amino acids	σ^W
SodA	2.1	detoxification of oxygen radicals	σ^B
SpoVG	2.9	cell division; control of sporulation initiation	σ^H, AbrB, SinR
YceC	18.0	unknown	σ^B, σ^M, σ^W, σ^X
YceH	22.8	unknown	σ^B, σ^M, σ^W, σ^X
YdbD	4.1	survival of ethanol stress	σ^B
YfhM	12.7	survival of ethanol stress	σ^B, σ^W
YfjR	3.1	unknown	YclJ
YjdA	3.8	unknown	
YoxD	4.9	unknown	
YthP	2.2	unknown	σ^W
YvlB	13.0	unknown	σ^W

Tab. S32 Marker proteins of mupirocin. Given are averages of regulation factors of three independent biological replicates. Protein functions and regulation are taken from the SubtiWiki database[1].

protein	regulation factor	function	regulation
OhrB	2.8	organic peroxide resistance	σ^B
PycA	7.9	replenishment of the oxaloacetate pool	σ^A, TnrA, YofA, stringent response
Tpx	6.0	unknown	Spx
YdaG	5.2	survival of stress conditions	σ^B
YtxH	6.5	unknown	σ^B, σ^H
IlvC	2.2	biosynthesis of branched-chain amino acids	σ^A, CcpA, CodY, TnrA, T-box
SpoVG	4.6	cell division; control of sporulation initiation	σ^H, AbrB, SinR
YlbA	3.1	unknown	AbrB
AroA	3.0	biosynthesis of aromatic amino acids	
PstS	4.3	high-affinity phosphate uptake	σ^A, PhoP
YukJ	9.1	unknown	
YvyD	18.2	dimerization of ribosomes in the stationary phase; protection against paraquat stress	σ^B, σ^D, σ^H, PhoP
YuaE	2.8	unknown	
YsnF	6.6	survival of ethanol stress	σ^B

Tab. S33 Marker proteins of nalidixic acid. Given are averages of regulation factors of three independent biological replicates. Protein functions and regulation are taken from the SubtiWiki database[1]. Superscripts indicate that the protein was identified in multiple spots on the gel image (see Fig. S27).

protein	regulation factor	function	regulation
Abh	3.2	transcriptional regulator of transition state genes	σ^M, σ^V, σ^X
AckA	4.0	overflow metabolism	σ^A, CcpA, CodY
AdcA	3.5	zinc uptake	σ^A, Zur
AhpF[1]	4.0	resistance against peroxide stress	σ^A, PerR
AhpF[2]	6.2		
Apt	2.9	purine salvage and interconversion	stringent response
CarB	8.9	biosynthesis of arginine	σ^A, AhrC, CodY
FabF	6.5	fatty acid biosynthesis	σ^A, σ^W, FapR
Fur	5.0	regulation of iron homeostasis	σ^A, PerR
HslO	3.7	unknown	σ^M
KatA	9.3	detoxification (degradation) of hydrogen peroxide	PerR
LysC	5.4	biosynthesis of lysine	L-box
Mbl	6.1	cell shape determination	σ^A, σ^E, stringent response
PyrK	5.5	pyrimidine biosynthesis	σ^A, PyrR
QueC	5.0	tRNA modification	preQ1 riboswitch
SpeA	5.4	spermidine, polyamine biosynthesis	
SufB[1]	2.6	synthesis of Fe-Sclusters	σ^A
SufB[2]	3.9		
SufC	3.3	synthesis of Fe-Sclusters	σ^A
TenA	5.2	thiamine salvage	Thi-box
TufA[1]	3.8	translation	stringent response
TufA[2]	2.9		
YfmT	5.4	unknown	σ^D
YhbJ	43.5	unknown	
YhcB	18.5	unknown	
YlbN	4.9	accumulation of 23S rRNA	stringent response
YkvM	4.8	tRNA modification	preQ1 riboswitch
YqfS	2.4	repair of oxidative DNA damage in spores	σ^G
YuaI	13.2	unknown	σ^W

Tab. S34 Marker proteins of nocathiacin I. Given are averages of regulation factors of three independent biological replicates. Protein functions and regulation are taken from the SubtiWiki database[1]. Superscripts indicate that the protein was identified in multiple spots on the gel image (see Fig. S28).

protein	regulation factor	function	regulation
FusA	2.6	translation	stringent response
GapA	4.8	catabolic enzyme in glycolysis	σ^A, CggR
Hag	3.5	motility and chemotaxis	σ^D, CodY, CsrA, ScoC
PurQ	8.1	purine biosynthesis	σ^A, PurR, G-box
RplD	13.1	translation	σ^A, stringent response
RplJ	4.5	translation	RplJ, RplL, stringent response
RplL	10.0	translation	RplJ, RplL, stringent response
RplR	4.0	translation	σ^A, stringent response
RpsB[1]	16.2	translation	stringent response
RpsB[2]	12.9		
RpsB[3]	6.4		
RpsB[4]	3.7		
RpsF[1]	5.7	translation	σ^A, ComK, stringent response
RpsF[2]	5.3		
SufB	5.2	synthesis of Fe-S clusters	σ^A
TufA[1]	10.1	translation	stringent response
TufA[2]	5.6		
YkrA	9.8	unknown	
YugL	3.0	enzyme in glycolysis/gluconeogenesis	

Tab. S35 Marker proteins of NV503. Given are averages of regulation factors of three independent biological replicates. Protein functions and regulation are taken from the SubtiWiki database[1].

protein	regulation factor	function	regulation
AzoR2	8.6	resistance to 2-methylhydroquinone	σ^A, σ^G, MhqR
BglH	4.6		
Ctc	2.7	unknown	σ^A, σ^B, stringent response
DltA	4.9	biosynthesis of teichoic acid	σ^D, σ^M, σ^V, σ^X, Spo0A, YvrHB, stringent response
FabZ	4.8	fatty acid biosynthesis	
Hom	6.6	biosynthesis of methionine and threonine	CodY, ThrR, TnrA
HslO	2.4	unknown	σ^M
IspF	3.6	MEP pathway of isoprenoid biosynthesis	σ^B, σ^M
LiaH	215.4	resistance against oxidative stress and cell wall antibiotics	σ^A, LiaR
MsrB	3.8	regeneration of methionine and restoration of protein function after oxidative damage	σ^A, Spx
NadE	5.4	NAD biosynthesis	σ^A, σ^B
NadF	4.5	generation of NADP from NAD	
NusA	3.8	transcription	σ^A, σ^B, NusA, stringent response
OdhB	6.6	TCA cycle	σ^A, CcpA
PdxS	3.1	pyridoxal-5-phosphate biosynthesis	Spo0A
PrmA	7.8	unknown	σ^A, HrcA
PspA	7.9	unknown	σ^W, AbrB
RacX	12.0	production of non-canonical D-amino acids	σ^W
RibH	3.2	riboflavin biosynthesis	σ^A, FMN-box
RimM	4.1	maturation of 16S rRNA	
RpsB	3.0	translation	stringent response
SdhA	4.5	TCA cycle	σ^A, FarA
SkfG	3.5	unknown	σ^A, AbrB, PhoP, Spo0A
ThrS	8.4	translation	σ^A, T-box
TrmK	5.7	tRNA modification	
TrxB	4.6	keeps TrxA in reduced state	Spx
YceC	7.7	unknown	σ^B, σ^M, σ^W, σ^X
YceH	2.9	unknown	σ^B, σ^M, σ^W, σ^X
YdbD	5.8	survival of ethanol stress	σ^B
YfjR	4.2	unknown	YclJ
YfmS	12.1	control of chemotaxis	σ^D
YhfE	3.5	unknown	
YjdA	5.8	unknown	
YphP	4.7	de-bacillithiolation of S-bacillithiolated OhrR and MetE	
YqeK	2.3	unknown	
YqkB	8.7	unknown	
YqkF	5.3	unknown	σ^E
YsnF	4.1	survival of ethanol stress	σ^B
YuaI	3.1	unknown	σ^W
YugI	4.9	unknown	stringent response
YvlB	5.0	unknown	σ^W
YwrO	2.9	unknown	
YxaB	3.5	biofilm formation; survival of salt and ethanol stress	σ^A, σ^B, AbrB

Tab. S36 Marker proteins of salvarsan. Given are averages of regulation factors of three independent biological replicates. Protein functions and regulation are taken from the SubtiWiki database[1].

protein	regulation factor	function	regulation
AbrB	3.0	regulation of gene expression during the transition from growth to stationary phase	σ^A, AbrB, Spo0A
AmpS	4.2	required for biofilm formation	
ArsC	38.2	detoxification of arsenate	σ^A, σ^F, ArsR
Cah	32.7	resistance to cephalosporin C	
Cmk	8.8	synthesis of CTP and dCTP	
CysC	3.2	sulfate reduction and activation	σ^A, CymR, S-box
CysK	12.9	biosynthesis of cysteine, control of CymR activity	σ^A, σ^M, CymR, Spx
GreA	3.6	transcription elongation	
GroEL	9.1	protein folding and re-folding	σ^A, HrcA
GroES	8.3	protein folding and re-folding	σ^A, HrcA
HemH	5.7	biosynthesis of heme	
IolS	4.6	unknown	σ^A, IolR
MccB	18.7	methionine-to-cysteine conversion	σ^A, CymR, Spx
NusA	8.1	transcription	σ^A, σ^B, NusA, stringent response
PepF	5.1	protein degradation	
PtsI	11.1	PTS-dependent sugar transport	σ^A, GlcT, stringent response
PyrF	3.2	pyrimidine biosynthesis	σ^A, PyrR
RplL	12.0	translation	RplJ, RplL, stringent response
RplR	5.3	translation	σ^A, stringent response
SalA	18.7	control of alkaline protease expression	SalA
SodA	4.7	detoxification of oxygen radicals	σ^B
SpoVG	2.8	cell division, control of sporulation initiation	σ^H, AbrB, SinR
YfkO	4.2	protection against NaOCl stress	σ^A, HypR
YlaL	3.8	unknown	
YmaB	3.5	unknown	σ^A, NrdR, PerR, ResD
YocJ	4.2	quinone detoxification	YodB
YuaE	7.4	unknown	
YvrD	13.7	unknown	
YvyD	4.8	dimerization of ribosomes in the stationary phase, protection against paraquat stress	σ^B, σ^D, σ^H, PhoP
YwfI	3.6	lipoic acid metabolism	
YxeK	12.3	utilization and detoxification of S-(2-succino)cysteine	σ^A, CymR

Tab. S37 Marker proteins of squalamine. Given are averages of regulation factors of three independent biological replicates. Protein functions and regulation are taken from the SubtiWiki database[1].

protein	regulation factor	function	regulation
LiaH	3.5	resistance against oxidative stress and cell wall antibiotics	σ^A, LiaR
PrmA	4.4	unknown	σ^A, HrcA
SdhA	5.8	TCA cycle	σ^A, FarA
YqkF	8.8	unknown	σ^E
YxaB	2.4	biofilm formation; survival of salt and ethanol stress	σ^A, σ^B, AbrB

Tab. S38 Marker proteins of telithromycin. Given are averages of regulation factors of three independent biological replicates. Protein functions and regulation are taken from the SubtiWiki database[1].

protein	regulation factor	function	regulation
AdcA	4.2	zinc uptake	σ^A, Zur
Adk	2.5	ADP formation	σ^A, stringent response
DivIVA	21.8	septum placement	Spo0A
DnaK	4.8	protein quality control	σ^A, HrcA
Eno	13.9	enzyme in glycolysis/gluconeogenesis	σ^A, CggR
Gid	6.1	tRNA modification	ComK, DnaA
IolS	9.7	unknown, may be involved in myo-inositol catabolism	σ^A, IolR
LeuS	2.5	translation	T-box
MetE	4.1	biosynthesis of methionine	S-box
OppA	6.2	initiation of sporulation; competence development	CodY, ScoC, TnrA
PdhD	9.3	links glycolysis and TCA cycle; enzyme in TCA cycle	σ^A, stringent response
Pgk	11.5	enzyme in glycolysis/gluconeogenesis	σ^A, CggR
PrfA	8.1	translation	
PupG	6.8	purine salvage and interconversion	σ^A, CcpA
PyrAA	3.9	pyrimidine biosynthesis	σ^A, PyrR
RplJ	6.9	translation	RplJ, stringent response
SufD	2.5	synthesis of Fe-S-clusters	σ^A
TpiA	30.1	enzyme in glycolysis/gluconeogenesis	σ^A, CggR
TufA	12.9	translation	stringent response
YceE	2.7	required for survival of ethanol stress and at low temperatures	σ^B, σ^M, σ^W, σ^X
YhdK	9.6	control of σ^M activity	σ^A, σ^M
YitJ	2.6	methionine biosynthesis; tetrahydrofolate interconversion	S-box
YqfS	7.9	repair of oxidative DNA damage in spores	σ^G
YrpE	2.9	zinc-binding protein	σ^A, Zur
YxjG	2.3	unknown	S-box

Tab. S39 Marker proteins of tunicamycin. Given are averages of regulation factors of three independent biological replicates. Protein functions and regulation are taken from the SubtiWiki database[1]. Superscripts indicate that the protein was identified in multiple spots on the gel image (see Fig. S33).

protein	regulation factor	function	regulation
AhpC	3.6	resistance against peroxide stress	σ^A, PerR
AhpF[1]	3.2	resistance against peroxide stress	σ^A, PerR
AhpF[2]	3.1		
CysC	3.7	sulfate reduction and activation	σ^A, CymR, S-box
DhbB	102.8	biosynthesis of the siderophore bacillibactin	σ^A, AbrB, Fur, Kre
GuaB	8.3	biosynthesis of GMP	CodY
MntA	4.9	manganese uptake	MntR
NagBB	2.4	N-acetylglucosamine utilization	σ^A, CcpA, NagR
PspA	3.9	unknown	σ^W, AbrB
RacX	5.0	production of non-canonical D-amino acids	σ^W
TasA	4.0	biofilm formation	σ^A, AbrB, LutR, RemA, SinR
YceC	2.7	unknown	σ^B, σ^M, σ^W, σ^X
YdbD	2.5	survival of ethanol stress	σ^B
YgxA	4.9	unknown	σ^E, σ^F
YqiG	3.2	unknown	
YrhB	5.3	methionine-to-cystein conversion	σ^A, CymR, Spx
YugK	3.1	unknown	
YvlB	10.3	unknown	σ^W

Tab. S40 Marker proteins of UC41. Given are averages of regulation factors of three independent biological replicates. Protein functions and regulation are taken from the SubtiWiki database[1].

protein	regulation factor	function	regulation
FabZ	2.5	fatty acid biosynthesis	
FosB	8.7	confers resistance against antimicrobial compounds form *B. amyloliquefaciens*	σ^W
GamA	5.7	glucosamine utilization	σ^A, GamR
GroEL	3.4	protein folding and re-folding	σ^A, HrcA
LiaH	19.0	resistance against oxidative stress and cell wall antibiotics	σ^A, LiaR
MrgA	11.2	iron storage	σ^A, PerR
MurB	2.5	peptidoglycan precursor biosynthesis	σ^A, σ^E, σ^M, SpoIIID, stringent response
NadE	10.7	NAD biosynthesis	σ^A, σ^B
NfrA	5.7	unknown	σ^A, σ^D, Spo0A, Spx
PbpC	10.0	unknown	
PspA	23.0	unknown	σ^W, AbrB
SodA	4.0	detoxification of oxygen radicals	σ^B
Spo0M	5.5	sporulation	σ^H, σ^W
TrmB	13.8	tRNA modification	
YceH	11.8	unknown	σ^B, σ^M, σ^W, σ^X
YjdA	3.5	unknown	
YoxD	11.8	unknown	
YvlB	5.6	unknown	σ^W

Tab. S41 nUPLC-ESI-MS-based protein identification for amidochelocardin 5, 10, and 20 µg/ml. Protein names were taken from SubtiWiki database[1]. Superscripts indicate that the protein was identified in multiple spots on the gel image (see Fig. S2).

protein	protein name	MW [Da]	pI	PLGS score	peptides	coverage [%]
AccB	acetyl-CoA carboxylase	17217	4.4	2363	6	60
AhpC[1]	alkyl hydroperoxide reductase (small subunit)	20614	4.3	64870	42	90
AhpC[2]				21997	32	71
Dps	mini-ferritin	16583	4.4	1218	2	13
GreA	transcription elongation factor	17260	4.5	2477	7	68
LpdV	dihydrolipoamide dehydrogenase (E3 subunit)	50404	5.0	8039	50	74
MccA	O-acetylserine-thiol-lyase	32997	5.2	1593	33	69
MtnB	methylthioribulose-1-phosphate dehydratase	23474	5.7	8578	31	72
PanB	2-methyl-2-oxobutanoate hydroxymethyltransferase	29739	5.3	14071	37	89
PdxT	pyridoxal-5-phosphate synthase	21433	5.0	13859	6	44
RibH	riboflavin synthase (beta subunit)	16276	5.2	36364	49	92
RsbV	anti-anti-σB	11931	4.7	12444	21	84
SodA	superoxide dismutase	22476	5.1	53335	30	89
SufB	FeS scaffold, synthesis of Fe-S-clusters	52696	5.0	2512	44	64
ThiD	4-amino-5-hydroxymethyl-2-methylpyrimidine pyrophosphate kinase	29105	5.7	14039	61	93
TrxA	thioredoxin	11385	4.3	15787	31	68
YhdN	aldo/keto reductase	37289	4.8	380	12	38
YtoP	similar to endo-1.4-beta-glucanase	39211	5.7	3629	30	73
YvlB	unknown	41055	5.4	4151	25	56
YxxG	immunity protein	16418	4.4	10624	40	92

Tab. S42 nUPLC-ESI-MS-based protein identification for AN3334. Protein names were taken from SubtiWiki database[1].

protein	protein name	MW [Da]	pI	PLGS score	peptides	coverage [%]
AroA	3-deoxy-D-arabino-heptulosonate 7-phosphate synthase	39514	5.3	1287	26	62
BacB	oxidase	26822	5.2	526	22	67
GsiB	general stress protein	13789	5.1	3946	29	75
IlvC	ketol-acid reductoisomerase (2,3-dihydroxy-3methlybutanoate,2-acetolacetat)	37434	5.4	20494	107	82
LeuD	3-isopropylmalate dehydratase (small subunit)	23007	4.6	9069	39	76
NfrA	FMN-containing NADPH-linked nitro/flavin reductase	28302	5.7	2190	17	58
SpoVG	negative effector of asymmetric septation	10886	5.1	2393	11	64
TrxA	thioredoxin	11385	4.3	3407	11	68
YfkM	glyoxalase III-like enzyme	18851	4.7	3237	25	59
YlbA	unknown	13484	5.0	2163	9	69
YneT	unknown	14979	5.3	12943	26	70
YtxH	unknown	16544	5.1	7754	26	69
YuaE	unknown	19098	6.2	4991	22	69
YvyD	ribosome hibernation promoting factor	21966	5.2	6768	40	68

Tab. S43 nUPLC-ESI-MS-based protein identification for anhydrotetracycline 0.5, 2, and 4 µg/ml. Protein names were taken from SubtiWiki database[1].

protein	protein name	MW [Da]	pI	PLGS score	peptides	coverage [%]
Abh	transcriptional regulator of transition state genes	10249	5.8	16707	9	52
DefB	N-formylcysteine deformylase	20642	5.5	6591	19	74
FabD	malonyl CoA-acyl carrier protein transacylase	34013	4.5	11683	50	85
FlgK	flagellar hook-filament junction proteins	54321	4.4	18979	60	84
GcaD	bifunctional N-acetylglucosamine-1-phosphate uridyltransferase/glucosamine-1-phosphate acetyltransferase	49423	5.7	1584	11	15
HemE	uroporphyrinogen decarboxylase (uroporphyrinogen III)	39621	5.3	5542	31	68
HprT	hypoxanthine phosphoribosyltransferase	20226	4.6	6127	17	72
MurAA	UDP-N-acetylglucosamine 1-carboxyvinyltransferase	46671	5.5	1584	29	48
MurF	UDP-N-acetylmuramoyl-L-alanyl-D-glutamyl-meso-2,6-diaminopimeloyl-D-alanyl-D-alanine synthetase	49691	5.3	800	14	44
Nfo	type IV apurinic/apyrimidinic endonuclease	33047	5.4	355	14	30
OhrB	general stress protein	14591	4.7	5660	11	82
OpuAA	glycin betain and arsenobetaine ABC transporter (ATP-binding protein)	43565	4.7	17777	91	68
PanB	2-methyl-2oxobutanoate hydroxymethyltransferase	29739	5.3	402	4	32
PanC	pantothenate synthase	31928	4.6	4213	29	55
PpiB	peptidyl-prolyl isomerase	15246	5.5	2458	8	34
PtsH	histidine-containing phosphocarrier protein HPr of PTS	9183	4.6	3706	6	85
PurB	adenylsuccinate lyase	49453	5.8	8215	50	59
PurC	phosphoribosylaminoimidazole succinocarboxamide synthase	27443	4.9	2092	13	49
SpoVG	negative effector of asymetric septation	10886	5.1	19921	30	89
ThrC	threonine synthase	37440	5.2	557	5	13
YdgI	unknown	23344	4.7	1130	12	40
YoxD	unknown	25283	5.3	14514	42	69
YpuA	unknown	31275	4.5	15963	41	73
YqeY	unknown	16755	5.5	2062	7	49
YqiK	cytoplasmic glycerophosphodiester phosphodiesterase	27102	5.9	644	9	37
YtpR	unknown	21682	4.3	1974	11	78
YtsP	unknown	18057	4.4	2783	20	68
YuaE	unknown	19098	6.2	3456	23	54
YvlB	unknown	41055	5.4	852	16	32

Tab. S44 nUPLC-ESI-MS-based protein identification for As_2O_3. Protein names were taken from SubtiWiki database[1].

protein	protein name	MW [Da]	pI	PLGS score	peptides	coverage [%]
AhpF	alkyl hydroperoxide reductase/NADH dehydrogenase	54839	4.7	1302	20	31
Ald	L-alanine dehydrogenase	39658	5.2	3551	20	60
AmpS	aminopeptidase	45769	5.0	2138	10	20
ArsC	arsenate reductase	15585	4.7	3898	13	57
ClpC	AAA unfoldase; ATPase subunit of the ClpC-ClpP protease	90063	5.7	3732	45	48
CysS	cysteine-tRNA synthetase	53873	5.1	1999.	30	47
GroEL	chaperonin; co-repressor for HrcA	57388	4.5	15563	174	86
GrpE	heat-shock protein	21669	4.3	3557	13	31
Hag	flagellin protein	32606	4.8	1852	3	13
KatA	vegetative catalase	54757	6.1	4687	70	59
PdhA	pyruvate dehydrogenase	41522	5.8	264	9	28
Pdp	pyrimidine nucleoside phosphorylase	46178	4.8	924	12	24
PepF	oligoendopeptidase	77024	5.5	7798	53	50
PnpA	polynucleotide phosphorylase	77415	4.9	3311	30	23
PspA	unknown	25125	5.8	276	2	8
PtsI	phosphotransferase system enzyme I	63039	4.6	11287	113	63
RpsD	ribosomal protein S4	22821	10.2	394	1	4
YfjR	3-hydroxyisobutyrate dehydrogenase	30454	4.9	1461	9	30
YkaA	unknown	23829	4.9	2780	17	30
YocJ	azoreductase	22962	5.0	5739	44	78
YqkC	unknown	16679	5.1	4049	12	41
YrhB	cystathionine lyase/ homocysteine gamma-lyase	40860	5.2	4290	26	40
YtcJ	unknown	58440	5.5	65	4	9
YutF	p-nitrophenyl phosphatase	27938	4.5	1231	6	23
YvyD	ribosome hibernation promoting factor	21966	5.2	2541	13	33
YwaA	branched-chain amino acid aminotransferase	40266	5.0	7315	82	69
YwfI	GcvH:E2 amidotransferase	28883	5.1	1655	9	20
YxeK	N-acetyl S-(2-succino)cysteine oxygenase	49308	5.7	2801	26	57
YxeP	N-acetylcysteine deacetylase	41526	5.3	403	1	2

Tab. S45 nUPLC-ESI-MS-based protein identification for As_2O_5. Protein names were taken from SubtiWiki database[1].

protein	protein name	MW [Da]	pI	PLGS score	peptides	coverage [%]
BkdAA	2-oxoisovalerate dehydrogenase	36310	4.8	1027	12	43
Cah	cephalosporin C deacetylase	35784	5.4	6521	48	67
Ctc	ribosomal protein	22042	4.2	5597	11	34
CysK	cysteine synthase	32799	5.5	4560	53	65
Dps	mini-ferritin	16583	4.4	4691	28	77
Eno	enolase	46552	4.5	2171	26	35
GroEL	chaperonin; co-repressor for HrcA	57388	4.5	1713	6	19
GsiB	general stress protein	13789	5.1	2452	8	47
Hag	flagellin protein	32606	4.8	11948	62	59
Spo0M	unknown	29714	4.3	4635	32	43
YfmS	soluble chemotaxis receptor	30305	4.5	5702	34	56
YhfK	unknown	22745	5.2	2680	14	64
YkwC	beta-hydroxyacid dehydrogenase	30691	5.1	500	8	30
YqfS	type IV apurinic/apyrimidinic endonuclease	33047	5.4	600	8	21
YtxH	unknown	16544	5.1	1442	7	36

Tab. S46 MALDI-TOF/TOF-based protein identification for ASD. Protein names were taken from SubtiWiki database[1]. Superscripts indicate when a protein was identified in multiple spots. Superscripts indicate that the protein was identified in multiple spots on the gel image (see Fig. S7).

protein	protein Name	MW [Da]	pI	peptides	protein score C.I.%
CspR	tRNA (Um34/Cm34) methyltransferase homolog	18574	6.1	10	100
Ctc	unknown	22042	4.4	8	100
Eno	enolase	46552	4.7	17	100
FabZ	β-hydroxyacyl-ACP dehydratase	14759	9.1	10	100
IspF	2-C-methyl-D-erythrol-2,4-cyclodiphosphate synthase	17115	5.5	13	100
LiaH	similar to phage shock protein	31591	5.3	11	100
MsrB	peptide methionine sulfoxide reductase	16591	5.9	7	100
OhrB	general stress protein	14591	4.9	3	100
PepF	oligopeptidase	70100	5.3	19	100
Pgm	2,3-bisphosphoglycerate-independent phsphoglycerate mutase	56274	5.3	17	100
PrmA	unknown	34573	4.8	7	100
PspA	phage shock protein A homolog	25125	5.9	2	100
PtsH	histidine-containing phosphocarrier protein HPr of the PTS	9184	4.9	6	100
RibH	riboflavin synthase (beta subunit)	16276	5.4	8	100
RimM	16S rRNA processing RNase	20190	5.4	8	100
RplL[1]	ribosomal protein L12	12743	4.6	7	100
RplL[2]				7	100
RplS	ribosomal protein L19	13739	10.8	5	100
Sat	sulfate adenylyltransferase	42857	5.7	22	100
SdhA	succinate dehydrogenase (flavoprotein subunit)	65308	5.8	20	100
SigX	RNA polymerase ECF-type sigma factor	23165	6.1	7	100
ThrC	threonine synthase	37440	5.3	12	100
TrmK	tRNA:m1A22 methyl transferase	23690	5.4	4	100
YceC	unknown	20754	5.5	1	100
YfmS	soluble chemotaxis receptor	30306	4.7	14	100
YjoA	unknown	17782	5.8	12	100
YphP	bacilliredoxin	15865	4.8	9	100
YqhS	similar to 3-dehydroquinate dehydratase	16421	5.3	6	100
YqiG	similar to NADH-dependent falvin oxidoreductase	40780	5.3	20	100
YqkB	unknown	11757	5.0	8	100
YqkF	NADPH-dependent 4-Hydroxy-2,3-trans-nonenal reducatese	34696	5.3	13	100
YsnF	unknown	32258	4.4	9	100
YutE	unknown	16640	4.8	6	100
YvlB	unknown	37434	5.5	17	100

Tab. S47 nUPLC-ESI-MS-based protein identification for auranofin. Protein names were taken from SubtiWiki database[1]. Superscripts indicate when a protein was identified in multiple spots. Superscripts indicate that the protein was identified in multiple spots on the gel image (see Fig. S8).

protein	protein name	MW [Da]	pI	PLGS score	peptides	coverage [%]
ArsC	arsenate reductase	15585	4.7	1050	10	30
Cah	cephalosporin C deacetylase	35784	5.4	14983	75	75
CitB	aconitase	99271	4.9	555	22	9
CitZ	citrate synthase II	41702	5.5	298	1	2
Hag	flagellin protein	32606	4.8	24064	71	91
KatA[1]	vegetative catalase	54757	6.1	3951	65	54
KatA[2]				1082	24	29
KatA[3]				2301	39	51
KatA[4]				9874	98	80
MrgA	mini-ferritin; DNA-binding stress protein	17321	4.6	6471	14	89
YjoA	bacillithiol S-transferase	17781	5.8	3853	13	42
YpfD	unknown	42376	4.6	1179	14	15
YrhB[1]	cystathionine lyase; homocysteine gamma-lyase	40860	5.2	727	7	16
YrhB[2]				7932	47	48
YuaE	bacillithiol S-transferase	19098	6.2	5659	35	62
YwkD	unknown	14823	5.4	436	2	6
YxeP	N-acetylcysteine deacetylase	41526	5.3	1040	8	13

Tab. S48 nUPLC-ESI-MS-based protein identification for BA234. Protein names were taken from SubtiWiki database[1]. Superscripts indicate that the protein was identified in multiple spots on the gel image (see Fig. S9).

protein	protein name	MW [Da]	pI	PLGS score	peptides	coverage [%]
AcsA[1]	acetyl-CoA synthetase	64850	5.5	883	26	39
AcsA[2]		64850	5.5	2712	60	39
AtpA	ATP synthase (subunit alpha)	54564	5.0	1227	17	31
CitZ	citrate synthase II	41702	5.5	1281	25	50
ClpP	ATP-dependent Clp protease proteolytic subunit	21668	5.0	6278	36	46
DisA	DNA integrity scanning protein; has diadenylate cyclase activity	40708	5.6	1018	14	44
FabG	beta-ketoacyl-acyl carrier protein reductase	25283	5.3	7521	36	71
FbaA	fructose-1,6-bisphosphate aldolase	30381	5.0	2947	35	74
GsiB	general stress protein	13789	5.1	293	1	7
HemH	coproporphyrin ferrochelatase	35325	4.6	1204	18	34
IolD	formation of 5-deoxy-D-glucuronic acid	70335	5.1	620	9	12
LiaH[1]	unknown	25682	6.2	6289	29	60
LiaH[2]				2590	14	48
LiaH[3]				915	4	20
LiaR	two-component response regulator	23110	5.0	1000	7	24
LpdV	2-oxoisovalerate dehydrogenase	50404	5.0	3032	40	44
MlpA	control of proteolytic activity	45965	5.3	844	14	31
PbpE	penicillin-binding protein PBP 4*	51404	4.8	2659	41	55
PspA[1]	unknown	25125	5.8	896	12	59
PspA[2]				4361	28	63
PspA[3]				1409	11	46
SalA	MRP family regulator	38614	5.2	1154	13	33
SpeE	spermidine synthase	31315	5.1	1251	24	66
TcyA	cystine and diaminopimelate ABC transporter (binding protein)	29495	9.0	726	3	12
TrxB	thioredoxin reductase	34497	5.0	9665	59	70
YaaQ	ci-di-AMP-binding PII-like protein	11959	6.1	6875	18	46
YbfO	unknown	51755	6.7	178	1	3
YceC	unknown	21809	5.3	3629	29	63
YceH	unknown	41646	5.7	5803	97	64
YdaG	putative pyridoxamine-5'-phosphate oxidase	15867	5.2	2005	8	44
YdbD	unknown	30238	4.9	4926	11	25
YdgI	unknown	23344	4.7	2096	14	47
YfhM	survival of ethanol stress	32737	6.1	2552	15	42
YhdN	aldo/keto reductase	17161	6.1	368	4	28
YhfE	unknown	38711	5.9	3406	47	42
YhfK	unknown	22745	5.2	3495	18	67
YknY	ABC transporter (ATP-binding protein)	25255	5.8	1272	9	33
YqiG	unknown	40779	5.2	2679	29	44
YsnF	unknown	30706	4.2	5449	40	75
YuaE	bacillithiol S-transferase	19098	6.2	6097	23	48
YuaI	unknown	19830	5.1	6731	19	66
YugJ	unknown	42705	5.2	9109	54	55
YvgN	glyoxal reductase	31643	5.1	5783	62	76
YvlB[1]	unknwon	41055	5.4	4747	28	50
YvlB[2]				3848	25	49
YwfI	GcvH:E2 amidotransferase	28883	5.1	3911	31	45
YwrO	unknown	19942	5.2	5802	32	70

Tab. S49 nUPLC-ESI-MS-based protein identification for CCCP. Part 1 of 2. Protein names were taken from SubtiWiki database[1]. Superscripts indicate that the protein was identified in multiple spots on the gel image (see Fig. S10).

protein	protein name	MW [Da]	pI	PLGS score	peptides	coverage [%]
AcuA	Gcn5-related N-acetyltransferase	24316	5.0	973	9	39
Ald	L-alanine dehydrogenase	39658	5.1	11216	79	70
BglA	6-phospho-beta-glucosidase	54806	4.6	1653	43	41
BglH	phospho-beta-glucosidase	53255	5.0	4515	74	46
Ctc	ribosomal protein	22042	4.2	8256	40	53
Def	formylmethionine deformylase	20642	5.5	1848	6	34
Dps	mini-ferritin	16583	4.4	11774	59	93
EtfA	electron transfer flavoprotein	34519	4.7	5108	58	58
EtfB	electron transfer flavoprotein	28499	4.2	6999	44	67
FadR	transcriptional repressor	21964	6.1	3828	31	41
FrlB	fructoselysine-6-P-glycosidase	36852	5.4	10338	48	46
GsiB	general stress protein	13789	5.1	2417	17	37
HisA	phosphoribosylformimino-5-aminoimidazole carboxamide ribotide isomerase	26512	4.8	10828	38	69
HisB	imidazoleglycerol-phosphate dehydratase	21535	5.6	4054	34	34
HisD	histidinol dehydrogenase	46227	4.8	6015	80	65
HisF	imidazole glycerol phosphate synthase	27277	5.2	4583	45	69
HisG	ATP phosphoribosyltransferase	23606	4.5	10498	55	85
HisH[1]	imidazole glycerol phosphate synthase	23287	5.7	2161	20	53
HisH[2]				3922	29	68
HisI	phosphoribosyl-AMP cyclohydrolase /phosphoribosyl-ATP pyrophosphohydrolase	23885	4.6	4821	26	47
LpdV	2-oxoisovalerate dehydrogenase	50404	5.0	2005	42	41
MelA	alpha-galactosidase	49231	5.8	592	6	15
MetN	methionine ABC transporter (ATP-binding protein)	30336	8.7	1072	14	32
MurF	UDP-N-acetylmuramoyl-L-alanyl-D-glutamyl-meso-2,6- diaminopimeloyl-D-alanyl-D-alanine synthetase	49691	5.3	2536	34	41
NagBB	glucosamine-6-phosphate deaminase	27271	5.8	4035	24	67
NfrA	FMN-containing NADPH-linked nitro/flavin reductase	28302	5.7	4462	24	45
OhrB	unknown	14591	4.7	2584	24	60
RocA[1]	3-hydroxy-1-pyrroline-5-carboxylate dehydrogenase	56284	5.6	1853	34	47
RocA[2]				4821	59	65
RsbW	anti-sigma factor; protein serine kinase	17981	4.3	9055	42	67
SacA	phosphosucrase	54779	5.4	2827	31	47
Tsf	elongation factor Ts	32333	5.0	727	10	20
TyrA	prephenate dehydrogenase	41408	5.5	2554	34	49

Tab. S50 nUPLC-ESI-MS-based protein identification for CCCP. Part 2 of 2. Protein names were taken from SubtiWiki database[1]. Superscripts indicate that the protein was identified in multiple spots on the gel image (see Fig. S10).

protein	protein name	MW [Da]	pI	PLGS score	peptides	coverage [%]
YaaQ	c-di-AMP-binding PII-like protein	11959	6.1	6160	21	46
YcsN	unknown	34118	5.3	3337	31	46
YdaE	lyxose isomerase	19239	4.9	1222	10	24
YdbD[1]	unknown	30238	4.9	3454	10	24
YdbD[2]				6757	32	30
YerA	unknown	66602	5.8	1489	39	45
YezE	putative transcriptional regulator	21873	4.9	1809	12	45
YfkM	glyoxalase III-like enzyme	18851	4.7	4841	50	54
YhdF	unknown	31489	5.2	4637	33	45
YhdN[1]	aldo/keto reductase	37289	4.8	13675	56	83
YhdN[2]				2795	10	34
YjbC	unknown	23105	5.1	2724	19	52
YocK	general stress protein	18819	4.3	6597	37	85
YpfD	unknown	42376	4.6	1583	20	30
YrbE	unknown	37764	5.7	2902	35	53
YsiB	3-hydroxyacyl-CoA dehydratase	27536	5.0	8800	50	60
YsnF	unknown	30706	4.2	15276	84	78
YtxH	unknown	16544	5.1	7830	70	72
YukJ	unknown	25538	4.4	2404	28	34
YvyD	ribosome hibernation promoting factor	21966	5.2	7030	57	68
YxaB	putative exopolysaccaride synthase	39739	4.5	8781	55	62
YxbG	unknown	29414	4.5	1788	15	48

Tab. S51 nUPLC-ESI-MS-based protein identification for cinnamaldehyde. Protein names were taken from SubtiWiki database[1]. Superscripts indicate that the protein was identified in multiple spots on the gel image (see Fig. S11).

protein	protein name	MW [Da]	pI	PLGS score	peptides	coverage [%]
Cah	cephalosporin C deacetylase	35784	5.4	6577.41	35	65
ClpP	ATP-dependent Clp protease proteolytic subunit	21668	5.0	6485.99	17	53
DnaK	class I heat-shock protein (molecular chaperone)	65962	4.6	11461.70	139	85
FabG	beta-ketoacyl-acyl carrier protein reductase	25283	5.3	10841.58	47	60
GrpE	heat-shock protein	21669	4.3	3224.89	19	31
IolS	unknown	35146	5.4	3316.54	33	55
LeuD	3-isopopylmalate dehydratase	23007	4.6	3468.53	37	34
NfrA	FMN-containing NADPH-linked nitro/flavin reductase	28302	5.7	1543.46	10	40
PbpC	penicillin-binding protein 3	74360	6.2	2116.73	28	29
PtsI	phosphotransferase system (PTS) enzyme I	63039	4.6	12297.67	169	76
SalA	MPR family regulator	38614	5.2	4399.83	40	45
SodA	superoxide dismutase	21433	5.0	20481.95	46	93
SuhB	nucleotidase	29741	5.1	4629.27	39	55
ThrS[1]	threonyl-tRNA synthetase (major)	73468	5.2	4710.60	106	64
ThrS[2]				1370.10	34	29
YaaE	pyridoxal-5-phasphae synthase (glutaminase domain)	22476	5.1	21574.71	44	73
YjdA	unknown	27431	5.7	1810.73	29	30
YqiG	unknown	40779	5.2	1079.60	13	15
YugJ	unknown	42705	5.2	6905.62	46	68
YulF	minor NADP-dependent scyllo-inositol dehydrogenase	36492	5.2	2027.01	34	33

Tab. S52 nUPLC-ESI-MS-based protein identification for ciprofloxacin. Protein names were taken from SubtiWiki database[1].

protein	protein name	MW [Da]	pI	PLGS score	peptides	coverage [%]
Apt	adenine phosphoribosyltransferase	18863	4.7	9476.61	24	66
Ctc	general stress protein	22042	4.2	17147.20	54	50
DinB	nuclease inhibitor	19891	5.6	6896.76	47	97
DnaC	Replicative DNA helicase	50544	4.6	226.183	21	54
EfeM	iron uptake system component	42769	4.7	8508.50	59	58
Fur	ferric uptake regulation protein	17416	5.3	4124.02	22	60
GreA	transcription elongation factor	17260	4.5	15277.65	37	79
GspA	general stress protein	33500	5.1	6633.55	48	98
GtaB	UTP-glucose-1-phosphate uridylyltransferase	33049	4.9	20579.84	106	88
OhrB	organic hydroperoxide resistance protein	14591	4.7	25773.42	29	77
Pgm	2,3-bisphosphoglycerate-independent phosphoglycerate mutase	56273	5.2	1695.051	35	58
PurH	bifunctional purine biosynthesis protein	55719	5.1	6233.26	53	65
RbsW	serine-protein kinase	17981	4.3	11352.46	49	75
ThiE	thiamine-phosphate synthase	23666	5.1	9791.47	33	61
YceD	general stress protein	20681	4.3	28209.68	69	95
YdaD	general stress protei	31062	4.8	2551.04	18	49
YdaG	general stress protein	15867	5.2	6482.83	33	73
YeeI	transcriptional regulator	26297	4.3	7310.90	30	73
YfhH	hypothetical protein	12001	4.6	34405.25	28	88
YlbN	hypothetical protein	19981	4.3	3427.48	10	74
YsnA	non-canonical purine NTP pyrophosphatase	21919	5.0	9920.10	45	75
YtxH	hypothetical protein	16544	5.1	5381.43	38	61
YtzG	16S rRNA pseudouridine synthase	26772	5.5	8460.87	39	78
YugI	general stress protein	14274	5.9	6192.22	9	78
YugK	NADH-dependent butanol dehydrogenase 2	43378	4.6	22443.02	157	83
YvaK	carboxylesterase	28392	5.0	3383.07	30	78
YvyD	sigma-54 modulation protein	21966	5.2	7333.49	49	59
YwqK	hypothetical protein	18205	4.5	2998.59	14	55

Tab. S53 nUPLC-ESI-MS-based protein identification for closthioamide. Protein names were taken from SubtiWiki database[1]. Superscripts indicate that the protein was identified in multiple spots on the gel image (see Fig. S13).

protein	protein name	MW [Da]	pI	PLGS score	peptides	coverage [%]
ArgS	arginyl-tRNA synthetase	62683	5.1	21560	109	85
AzoR2	azoreductase	23272	5.1	8975	35	76
GbsB[1]	choline dehydrogenase (FAD-dependent)	43392	4.9	13032	69	58
GbsB[2]		43392	4.9	3032	18	39
GlpX	class II fructose-1,6-bisphosphatase	33951	5.1	16122	59	61
GltX	glutamyl-tRNA synthetase	55687	5.0	16893	59	71
GuaA	GMP synthetase	57812	4.7	2731	28	47
IlvE	branched-chain amino acid aminotransferase	39651	5.0	13649	64	68
Maf	nucleotide pyrophosphotase	21281	5.7	2569	11	30
MenB	naphthoate synthase	29880	5.3	6887	20	46
MhqO	hydroquinone-specific dioxygenase	35056	4.8	14057	42	65
MnaA	UDP-N-acetylglucosamine 2-epimerase	42634	5.5	934	25	68
MrgA	mini-ferritin; DNA-binding stress protein	17321	4.6	6959	19	78
MtnK	5-methylthioribose kinase	45319	4.9	11999	55	60
MurD	UDP-N-acetylmuramoyl-L-alanyl-D-glutamate synthetase	49619	5.6	6297	82	61
MurQ	N-acetyl-muramic acid-6P etherase	32679	5.4	16531	28	68
NdoA	mRNA interferase	12969	4.9	13947	11	63
OhrA	peroxiredoxin	14858	5.1	18262	23	89
PatA	aminotransferase	43508	6.0	2992	28	44
PckA	phosphoenolpyruvate carboxykinase	58235	5.1	11399	71	66
PurA	adenylosuccinate synthetase	47642	5.5	10769	72	81
PurM	phosphoribosylaminoimidazole synthetase	37028	4.5	507	12	32
RapG	response regulator aspartate phosphatase	42842	4.8	1763	28	53
RibE	riboflavin synthase	23481	5.9	1069	17	53
ThiE	thiamine-phosphate pyrophosphorylase	23666	5.1	7015	23	59
ThiQ	sporulation protein	47123	5.0	5267	38	42
YcnD	NADPH-FMN oxidoreductase	27850	5.1	3343	7	22
YcnE	unknown	10937	4.9	14956	19	81
YdgI	unknown	23344	4.7	1167	12	40
YhdN	aldo/keto reductase, specific for NADP	37289	4.8	20705	45	75
YopQ	unknown	53504	5.2	3194	41	53
YopX	unknown	15184	4.3	8721	11	68
YorG	unknown	36603	4.8	5046	36	66
YsnF	unknown	30706	4.2	2891	29	80
YtpQ	unknown	31043	5.3	4022	29	49
YtsJ	NADP-dependent malate dehydrogenase	43639	5.1	6279	42	74
YxjG	putative methionine synthetase	43137	5.0	15658	81	77

Tab. S54 nUPLC-ESI-MS-based protein identification for cXRX. Protein names were taken from SubtiWiki database[1].

protein	protein name	MW [Da]	pI	PLGS score	peptides	coverage [%]
GroEL	chaperonin, co-repressof for HrcA	24422	4.5	35524	179	79
HslO	putative disulfide bon chaperone	31791	4.7	14737	53	93
MinD	cell division inhibitor	29388	5.0	8767	48	62
PepF	oligoendopeptidase	77024	5.5	8410	102	52
TpiA	triosphosphate isomerase	27012	4.8	4669	30	58

Tab. S55 nUPLC-ESI-MS-based protein identification for 5% DMSO. Protein names were taken from SubtiWiki database[1]. Superscripts indicate that the protein was identified in multiple spots on the gel image (see Fig. S15).

protein	protein name	MW [Da]	pI	PLGS score	peptides	coverage [%]
Adk	adenylate kinase	24104	4.4	8637	53	86
ClpC	AAA unfoldase; ATPase subunit of the ClpC-ClpP protease	90063	5.7	1722	41	37
ClpP	ATP-dependent Clp protease proteolytic subunit	21668	5.0	3599	23	26
DapA[1]	dihydrodipicolinate synthase	31022	4.9	2919	30	71
DapA[2]				6256	51	72
Dps[1]	mini-ferritin	16583	4.4	2392	16	30
Dps[2]				2715	14	30
Dps[3]				2303	16	36
Dps[4]				7096	40	74
FbaA[1]	fructose-1,6-bisphosphate aldolase	30381	5.0	1959	23	39
FbaA[2]				601	2	9
FbaA[3]				1234	13	20
GreA	transcription elongation factor	17260	4.5	5226	19	61
GrpE	heat-shock protein	21669	4.3	7387	42	41
GsiB	general stress protein	13789	5.1	2597	18	53
GtaB[1]	UTP-glucose-1-phosphate uridylyltransferase	33049	4.9	1344	14	33
GtaB[2]				7537	54	60
GtaB[3]				2766	31	53
IleS	isoleucyl-tRNA synthetase	104778	5.2	917	39	22
IlvD	dihydroxy-acid dehydratase (2,3-dihydroxy-3-methylbutanoate, 2,3-dihydroxy-3-methylpentanoate)	59462	5.2	1510	54	43
KatE	catalase 2	77431	5.8	1635	44	40
MalS	malate dehydrogenase (decarboxylating)	62110	4.9	5233	53	54
MetE	methionine synthase	86752	4.9	3606	62	43
OhrB	unknown	14591	4.7	1592	9	39
RpsC	ribosomal prtein S3	24307	10.1	12578	78	82
RsbW	anti-sigma factor, prtein serine kinase	17981	4.3	3096	30	75
Sat	sulfate adenylyltransferase	42856	5.6	2147	22	41
ThiD	4-amino-5-hydroxymethyl-2-methylpyrimidinepyrophosphate kinase	28998	4.9	2186	21	56
ThyA	thymidylate synthase A	32786	5.5	283	1	4
YcdF	unknown	27758	5.3	2118	10	22
YceE	unknown	20935	4.4	9800	23	53
YdaD	unknown	31062	4.8	8610	60	69
YdaE	lyxose isomerase	19239	4.9	2815	21	32
YeeI	unknown	26297	4.3	2464	15	33
YfkM[1]	glyoxalase III-like enzyme	18851	4.7	4902	42	44
YfkM[2]				5966	47	55
YhdF	unknown	31489	5.2	2301	27	36
YloC	unknown	33609	4.9	3193	31	45
YpfD[1]	unknown	42376	4.6	3145	37	48
YpfD[2]				1771	26	40
YsnF[1]	unknown	30706	4.2	20132	115	81
YsnF[2]				3703	28	66
YsnF[3]				5937	52	79
YsnF[4]				4636	39	60
YtxH	unknown	16544	5.1	9838	49	55
YvgN	glyoxal reductase	31643	5.1	1922	27	25
YvrE	glucoco-lactonase	33183	4.6	2964	40	50
YxaB[1]	putative exopolysaccaride synthase	39739	4.5	12229	80	80
YxaB[2]				3388	38	45

Tab. S56 nUPLC-ESI-MS-based protein identification for GE2270A. Protein names were taken from SubtiWiki database[1].

protein	protein name	MW [Da]	pI	PLGS score	peptides	coverage [%]
CheV	CheA modulator	34611	4.6	5698	45	84
Nfo	type IV apurinic/apyrimidinic endonuclease	33047	5.4	2878	31	52
QueF	nitrile reductase	19362	4.9	909	6	50

Tab. S57 nUPLC-ESI-MS-based protein identification for kirromycin. Protein names were taken from SubtiWiki database[1].

protein	protein name	MW [Da]	pI	PLGS score	peptides	coverage [%]
DnaK	class I heat-shock protein (molecular chaperone)	65962	4.6	14142	91	79
GrpE	heat shock protein	21669	4.3	2226	12	31
GuaC	GMP reductase	35827	6.1	6728	50	78
HemH	coproporphyrin ferrochelatase	35325	4.6	215	80	26
YhcB	unknown	19003	4.6	611	3	21
YojG	N-acetylglucosamin-malate deacetylase	25560	4.7	1016	13	44
YvlB	unknown	41055	5.4	7146	37	66

Tab. S58 nUPLC-ESI-MS-based protein identification for KKL-35 and KKL-40. Protein names were taken from SubtiWiki database[1]. Superscripts indicate that the protein was identified in multiple spots on the gel image (see Fig. S19 and S20).

protein	protein name	MW [Da]	pI	PLGS score	peptides	coverage [%]
AhpC[1]	alkyl hydroperoxide reductase (small subunit)	20614	4.3	23466	43	95
AhpC[2]		20614	4.3	12861	28	78
AhpF[1]	alkyl hydroperoxide reductase (large subunit)	54839	4.7	7685	113	64
AhpF[2]		54839	4.7	1243	34	36
AhpF[3]		54839	4.7	4532	57	52
AhpF[4]		54839	4.7	4549	62	51
BrxA	bacilliredoxin	32606	4.8	3311	10	45
CtC	ribosomal protein	22042	4.2	3515	13	41
DhbA	2,3-dihydro-2,3-dihydroxybenzoate dehydrogenase	27436	5.3	20634	63	80
Fur	transcriptional repressor Fur family	17416	5.3	3330	13	38
GreA	transcription elongation factor	17260	4.5	33270	39	79
GuaB	IMP dehydrogenase	52957	6.2	5642	50	70
HisA	phosphoribosylformimino-5-aminoimidazole carboxamide ribotide isomerase	26512	4.8	21854	48	79
HisG	ATP phosphoribosyltransferase	23606	4.5	5105	16	73
IscU	sulfur shuttle protein	16155	4.7	379	5	47
KatA[1]	vegetative catalase	54757	6.1	10683	84	63
KatA[2]		54757	6.1	1182	11	57
KatA[3]		54757	6.1	1453	27	40
MrgA	mini-ferritin, DANN-binding stress protein	17321	4.6	7532	15	57
NfrA	FMN-containing NADPH-linked nitro/flavin reductase	28302	5.7	11430	26	58
OhrB	general stress protein	14591	4.7	2542	10	51
SufD	Fes scaffold	48263	5.0	5971	41	58
Tpx	unknown	18204	4.7	4622	17	62
TrxA	thioredoxin	32606	4.8	2823	6	34
YcsN	unknown	34118	5.3	5954	27	46
YfkM	glyoxalase III-like enzyme	18851	4.7	8142	32	62
YhzC	unknown	9112	7.1	314	6	37
YtxH	general stress protein	16544	5.1	3952	18	78
YukJ	unknown	25538	4.4	1649	9	40
YvyD	ribosome hibernation promoting factor	21966	5.2	4178	16	56
YwrO	unknown	19942	5.2	4584	21	67

Tab. S59 nUPLC-ESI-MS-based protein identification for KKL-55. Protein names were taken from SubtiWiki database[1]. Superscripts indicate that the protein was identified in multiple spots on the gel image (see Fig. S21).

protein	protein name	MW [Da]	pI	PLGS score	peptides	coverage [%]
AhpC	alkyl hydroperoxide reductase (small subunit)	20614	4.3	14147	56	80
AhpF	alkyl hydroperoxide reductase (large subunit)/NADH dehydrogenase	54839	4.7	346	17	30
AzoR1	azoreductase	22962	5.0	1947	29	88
ClpP	ATP-dependent Clp protease proteolytic subunit	21668	5.0	893	13	56
DhbA	2,3-dihydro-2,3-dihydroxybenzoate dehydrogenase	27436	5.3	197	6	28
DhbB	isochorismatase	35084	4.4	2054	16	53
FlgK	flagellar hook-filament junction protein	54321	4.4	2968	25	53
GroEL[1]	chaperonin; co-repressor for HrcA	57388	4.5	1881	39	44
GroEL[2]				754	25	39
KatA	vegetative catalase	54757	6.1	379	10	16
NagB	glucosamine-6-phosphate deaminase	27271	5.8	330	6	28
PepF	oligoendopeptidase	77024	5.5	773	22	33
TenA	thiaminase II	27399	5.0	123	8	30
YcnD	NADPH-FMN oxidoreductase	27850	5.1	127	3	17
YhjG	unknown	54322	6.7	1027	26	36
YneT	unknown	14979	5.3	209	5	47
YqiG	unknown	40779	5.2	789	9	20
YugJ	unknown	42705	5.2	1395	17	40
YwrO	unknown	19942	5.2	3012	12	50

Tab. S60 nUPLC-ESI-MS-based protein identification for linezolid. Protein names were taken from SubtiWiki database[1]. Superscripts indicate that the protein was identified in multiple spots on the gel image (see Fig. S22).

protein	protein name	MW [Da]	pI	PLGS score	peptides	coverage [%]
Adk	adenylate kinase	24104	4.5	7371	40	66
CcpA	transcriptional regulator LacI family	36917	5.1	7342	87	89
CodY	transcriptional pleotropic repressor	28995	4.8	27872	.97	83
CspR	tRNA (Um34/Cm34) methyltransferase homolog	18565	5.9	1020	12	36
CwlO	enopeptidase-type autolysin	51002	5.3	4123	32	47
Fur	transcriptional repressor Fur family	17416	5.3	3268	19	43
HslO	putative disulfide bond chaperone	31791	4.7	8461	74	48
IolS	unknown	35146	5.4	10059	59	74
MnaA	UDP-N-acetylglucosamine 2-epimerase	42607	5.5	4271	28	57
NusG	sequence-specific RNA polymerase pause factor	20113	5.3	14781	46	69
PrfB	peptide chain release factor 2	42046	4.9	9751	77	84
PurC	phsphoribosylaminoimidazole succinocarboxamide synthase	27443	4.9	19134	88	90
PyrB	aspartate carbamoyltrasnferase	34202	5.5	5394	47	47
PyrC	dihydroorotase	46503	5.2	11305	79	83
RplJ	ribosomal protein L10	18067	5.2	28409	99	95
RpsF[1]	ribosomal protein S6	11117	5.0	21909	35	87
RpsF[2]		11117	5.0	7639	12	56
SpeB	agmanitase	32396	4.8	11935	69	68
SsbA	single-strand DNA-binding protein	18731	4.8	20221	34	80
SucD	succinyl-CoA synthetase (alpha subunit)	31362	5.6	1586	17	35
TagD	glycerol-3-phosphate cytidylyltransferase	15261	5.7	5247	30	68
TasA	major component of biofilm matrix	28287	5.4	2908	17	42
TrmK	tRNA:m1A22 methyl transferase	23690	5.2	8639	25	60
TrxB	thioredoxin reductase (NADPH)	34497	5.0	6903	38	54
YceE	general stress protein	20935	4.4	18711	38	46
YfmS	soluble chemotaxis receptor	30305	4.5	5673	17	52
YfmT	vanillin dehydrogenase	53287	5.1	2094	31	60
YjcJ	cystathionine beta-lyase	42464	5.8	8808	54	65
YlbN	unknown	19981	4.3	1944	6	41
YocH	peptidoglycan hydrolase	30163	8.8	5772	12	55
YutI	putative Fe-S scaffold protein	12416	4.2	26470	15	58
YwtE	unknown	31568	4.8	2790	24	72

Tab. S61 MALDI-TOF/TOF-based protein identification for monensin. Protein names were taken from SubtiWiki database[1].

protein	protein name	MW [Da]	pI	peptides	protein score C.I.%
AspS	aspartyl-tRNA synthetase	65934	4.9	33	100
CysK	cysteine synthase	32799	5.6	16	100
FolD	methylenetetrahydrofolate dehydrogenase	30666	5.7	17	100
FruC	fructokinase	34235	4.8	16	100
GcvPB	glycine decarboxylase	54393	5.4	14	100
GuaB	IMP dehydrogenase	52958	8.1	10	83
GudB	glutamate dehydrogenase; trigger enzyme	47182	5.7	9	100
HemL	glutamate-1-semialdehyde aminotransferase	46420	5.2	20	100
HisF	imidazole glycerol phosphate synthase	27277	5.4	14	100
HisZ	histidyl-tRNA synthetase	43477	5.3	17	100
IlvD	dihydroxy-acid dehydratase (2,3-dihydroxy-3-methylbutanoate, 2,3-dihydroxy-3-methylpentanoate)	59510	5.4	12	100
KtrC	low affinity potassium transporter KtrC-KtrD; peripheric membrane component	24333	5.7	11	100
LuxS	S-ribosylhomocysteine lyase	17703	5.3	7	100
MetC	cystathionine beta-lyase	42465	5.8	18	100
PatA	aminotransferase	43421	6.0	2	100
PdxS	pyridoxal-5-phosphate synthase	31591	5.3	15	100
SpoVC	peptidyl-tRNA hydrolase	20859	5.7	12	100
TyrS	tyrosyl-tRNA synthetase	47708	5.4	18	100
YceC	unknown	21810	5.5	13	100
YceH	unknown	41646	5.9	24	100
YfmS	soluble chemotaxis receptor	30306	4.7	14	100
YhcB	unknown	19003	4.8	7	100
YhfK	unknown	22746	5.3	12	100
YjbI	unknown	15106	5.3	7	100
YkuU	alkyl hydroperoxide reductase	20434	4.7	6	100
YqeY	unknown	16756	5.7	14	100
YtiB	unknown	21098	6.2	13	100
YuaE	bacillithiol S-transferase	19099	6.2	8	100
YwfI	coproheme decarboxylase	29486	5.2	11	100
YwlF	ribose-5-phosphate isomerase	16200	5.5	10	100

Tab. S62 MALDI-TOF/TOF-based protein identification for L-MP66, D-MP66, and L-MP159. Protein names were taken from SubtiWiki database[1].

protein	protein name	MW [Da]	pI	peptides	protein score C.I.%
AzoR2	azoreductase	23257	5.3	7	100
CitZ	citrate synthase II	41702	5.6	16	100
ClpP	ATP-dependent Clp protease proteolytic subunit	21668	5.2	12	100
DltA	D-alanyl-D-alanine carrier protein ligase	55773	5.1	13	100
Dps	mini-ferritin	16583	4.6	8	100
TufA	elongation factor Tu	44565	4.9	6	100
GsiB	general stress protein	13789	5.3	5	100
IolS	unknown	35146	5.5	16	100
LiaH	unknown	25682	6.2	13	100
MrgA	mini-ferritin; DNA-binding stress protein	17322	4.8	3	100
NadE	NH_3-dependent NAD^+ synthetase	30376	5.1	16	100
PspA	unknown	25125	5.9	11	100
RacX	amino acid racemase	25270	5.5	3	100
SodA	superoxide dismutase	22561	5.3	6	100
SpoVG	negative effector of asymetric septation	10886	5.3	10	100
YceC	unknown	21810	5.5	14	100
YceH	unknown	41646	5.9	13	100
YdbD	unknown	30238	5.1	10	100
YhdN	aldo/keto reductase, specific for NADP	3789	5.0	10	100
YjdA	unknown	27432	5.7	5	99
YoxD	unknown	25283	5.5	20	100
YqiW	bacilliredoxin	16186	5.0	7	100
YqkF	NADPH-dependent 4-Hydroxy-2,3-trans-nonenal reducstase	34695	5.3	10	100
YthP	ABC transporter (ATP-binding protein)	26490	5.4	5	100
YtkL	unknown	24816	5.1	8	100
YuaI	unknown	19830	5.3	7	100
YvlB	unknown	41056	5.5	15	100
YwrO	unknown	19942	5.3	7	100

Tab. S63 nUPLC-ESI-MS-based protein identification for mupirocin. Protein names were taken from SubtiWiki database[1].

protein	protein name	MW [Da]	pI	PLGS score	peptides	coverage [%]
IlvC	ketol-acid reductoisomerase (2,3-dihydroxy-3-methlybutanoate,2-acteolactate)	37434	5.4	19820	100	85
OhrB	general stress protein	14591	4.7	1198	18	79
PycA	pyruvate carboxylase	127856	5.4	4037	158	71
Tpx	unknown	18204	4.7	16285	33	85
YdaG	general stress protein	15867	5.2	4615	18	81
YtxH	general stress protein	16544	5.1	5773	30	65

Tab. S64 nUPLC-ESI-MS-based protein identification for nalidixic acid. Protein names were taken from SubtiWiki database[1]. Superscripts indicate that the protein was identified in multiple spots on the gel image (see Fig. S27).

protein	protein name	MW [Da]	pI	PLGS score	peptides	coverage [%]
Abh	transition state regulator	10249	5.8	40401	10	51
AckA	acetate kinase	43110	5.2	7460	51	73
AdcA	zinc-binding lipoprotein	35637	5.2	9968	55	45
AhpF[1]	NADH dehydrogenase	54839	4.7	13589	151	79
AhpF[2]				1520	30	64
Apt	adenine phosphoribosyltransferase	18863	4.7	5576	14	45
CarB	carbamoyl-phosphate synthase pyrimidine-specific large chain	117576	4.8	429	26	27
FabF	3-oxoacyl-[acyl-carrier-protein] synthase	43977	4.8	5099	35	77
Fur	ferric uptake regulation	17416	5.3	2888	12	37
HslO	chaperonin	31791	4.7	5586	52	47
KatA	vegetative catalase	54757	6.1	6034	63	64
LysC	aspartokinase	43780	4.6	1140	23	19
Mbl	MreB-like protein	35838	5.7	2307	23	50
PyrK	dihydroorotate dehydrogenase B	28081	5.6	2207	23	57
QueC	7-cyano-7-deazaguanine synthase	24513	5.2	4765	25	58
SpeA	arginine decarboxylase	53549	5.1	591	15	25
SufB[1]	FeS cluster assembly protein	52696	5.0	1260	31	41
SufB[2]				1339	33	46
SufC	ABC transporter ATP-binding protein	29012	4.6	7620	60	79
TenA	thiaminase	27399	5.0	5632	36	72
TufA[1]	elongation factor Tu	43565	4.7	26544	239	88
TufA[2]				4083	39	52
YfmT	aldehyde dehydrogenase	53287	5.1	393	7	14
YhbJ	efflux system component	23607	4.4	2389	20	29
YhcB	hypothetical protein	19003	4.6	5906	20	42
YkvM	7-cyano-7-deazaguanine reductase	19362	4.9	2539	13	48
YlbN	hypothetical protein	19987	4.3	3585	11	55
YqfS	endonuclease IV	33047	5.4	2697	17	52
YuaI	N-acetyltransferase	19830	5.2	1957	3	17

Tab. S65 nUPLC-ESI-MS-based protein identification for nocathiacin I. Protein names were taken from SubtiWiki database[1]. Superscripts indicate that the protein was identified in multiple spots on the gel image (see Fig. S28).

protein	protein name	MW [Da]	pI	PLGS score	peptides	coverage [%]
FusA	elongation factor G	76568	4.6	4624	101	45
GapA	glyceraldehyde 3-phosphate dehydrogenase	35810	5.0	10501	33	81
Hag	flagellin protein	32606	4.8	49890	114	88
PurQ	phsphoribosylformylglycinamidine synthase	24768	4.6	1863	5	17
RplD	ribosomal protein L4	22377	10.5	19258	52	71
RplJ	ribosomal protein L10	12742	4.4	28422	12	84
RplL	ribosomal protein L12	12742	4.4	89820	110	93
RplR	ribosomal protein L18	12960	10.5	1342	5	33
RpsB[1]	ribosomal protein S2	27949	6.3	10424	52	56
RpsB[2]				735	9	30
RpsB[3]				13434	73	81
RpsB[4]				36171	127	89
RpsF[1]	ribosomal protein S6	11117	5.0	9751	31	87
RpsF[2]				4618	8	42
SufB	Fe-S carrier protein	52696	5.0	2721	38	48
TufA[1]	elongation factor Tu	43565	4.7	36386	154	82
TufA[2]				17777	91	68
YkrA	unknown	28886	4.6	4206	21	39
YugL	glucose-6-phosphate isomerase	14274	5.9	29701	42	79

Tab. S66 MALDI-TOF/TOF-based protein identification for NV503. Protein names were taken from SubtiWiki database[1].

protein	protein name	MW [Da]	pI	peptides	protein score C.I.%
BglH	phospho-beta-glucosidase	48644	5.1	19	100
Ctc	ribosomal protein	22042	4.4	8	100
FabZ	β-hydroxyacyl-ACP dehydratase	14759	9.1	10	100
Hom	homoserine dehydrogenase (NADPH)	47465	5.1	17	100
HslO	putative disulfide bon chaperone	31791	4.9	14	100
IspF	2-C-methyl-D-erythrol-2,4-cyclodiphosphate synthase	17115	5.5	13	100
LiaH	unknown	31591	5.3	11	100
MsrB	peptide methionine sulfoxide reductase	16591	5.9	7	100
NadF	inorganic polyphosphate/ATP-NAD kinase	29993	6.0	15	100
NusA	transcription termination factor	41700	4.8	18	100
OdhB	2-oxoglutarate dehydrogenase complex	45960	5.1	9	100
PdxS	pyridoxal-5-phosphate synthase (synthase domain)	31591	5.3	15	100
PrmA	unknown	34573	4.8	7	100
PspA	phage shock protein A homolog	25125	5.9	2	100
RibH	riboflavin synthase (beta subunit)	16276	5.4	8	100
RimM	16S rRNA processing RNase	20190	5.4	8	100
SdhA	succinate dehydrogenase	65308	5.8	20	100
SkfG	unknown	14468	5.3	4	100
ThrS	threonmyl-tRNA synthetase (major)	73468	5.4	9	100
TrmK	tRNA:m1A22 methyl transferase	23690	5.4	4	100
TrxB	thioredoxin reductase (NADPH)	34498	5.2	19	100
YceC	unknown	20754	5.5	1	100
YfjR	3-hydroxyisobutyrate dehydrogenase	27848	5.0	5	100
YfmS	soluble chemotaxis receptor	30306	4.7	14	100
YhfE	unknown	38712	5.9	12	100
YphP	bacilliredoxin	15865	4.8	9	100
YqeK	unknown	21269	6.0	15	100
YqkB	unknown	11757	5.0	8	100
YqkF	NADPH-dependent 4-Hydroy-2,3-trans-nonenal reductase	34696	5.3	13	100
YsnF	unknown	32258	4.4	9	100
YugI	unknown	14274	6.0	9	100
YvlB	unknown	37434	5.5	17	100
YxaB	putative exopolysaccaride synthase	39738	4.7	14	100

Tab. S67 nUPLC-ESI-MS-based protein identification for salvarsan. Protein names were taken from SubtiWiki database[1].

protein	protein name	MW [Da]	pI	PLGS score	peptides	coverage [%]
GroEL	chaperonin; co-repressor for HrcA	57425	4.5	2942	24	41
PtsI	phosphotransferase system (PTS) enzyme I	63193	4.6	6078	42	64
PepF	oligoendopeptidase	77187	5.6	1843	31	38
AmpS	aminopeptidase	45798	5.0	424	8	16
YxeK	*N*-acetyl S-(2-succino)cysteine oxygenase	49369	5.7	1941	13	26
NusA	transcription termination factor	41782	4.6	767	11	26
MccB	cystathionine lyase/homocysteine gamma-lyase	44886	5.2	647	8	21
SalA	MRP family regulator	38639	5.2	728	8	38
HemH	coproporphyrin ferrochelatase	35405	4.6	612	4	11
IolS	unknown	35168	5.4	798	10	30
Cah	cephalosporin C deacetylase	35807	5.4	3748	19	59
CysK	cysteine synthase	32820	5.5	9650	28	69
YwfI	GcvH:E2 amidotransferase	28959	5.1	1685	7	29
YvrD	unknown	28222	4.7	5953	14	43
YmaB	unknown	23388	4.5	1174	10	44
Cmk	cytidylate kinase (CMP, dCMP)	25097	5.0	4410	11	46
YvyD	ribosome hibernation promoting factor	21980	5.2	2539	6	49
PyrF	orotidine-5-phosphate decarboxylase	26106	5.8	1011	13	67
YfkO	NAD(P)H-flavin nitroreductase	26585	5.7	2531	15	49
CysC	adenylyl-sulfate kinase	22715	5.1	6362	14	71
YocJ	azoreductase	22977	5.0	1999	15	40
SodA	superoxide dismutase	22490	5.1	1712	11	63
YuaE	bacillithiol S-transferase	19111	6.2	592	4	25
GreA	transcription elongation factor	17272	4.5	3242	8	59
YlaL	unknown	17952	5.2	1829	11	73
RplR	ribosomal protein L18	12969	10.5	592	3	24
ArsC	arsenate reductase	15824	4.8	4440	11	57
SpoVG	negative effector of asymetric septation	10893	5.1	2356	8	58
RplL	ribosomal protein L12 (BL9)	12751	4.4	24735	12	58
GroES	chaperonin	10176	4.6	21127	13	93
AbrB	transcriptional regulator	10830	6.8	374	3	27

Tab. S68 MALDI-TOF/TOF-based protein identification for squalamine. Protein names were taken from SubtiWiki database[1].

protein	protein Name	MW [Da]	pI	peptides	protein score C.I.%
LiaH	unknown	31591	5.26	11	100
PrmA	unknown	34573	4.79	7	100
SdhA	succinate dehydrogenase (/flavoprotein subunit)	65308	5.77	20	100
YqkF	NADP-dependent 4-Hydroxy-2,3-trans-nonenal reductase	34696	5.30	13	100
YxaB	putative exopolysaccaride synthase	39738	4.72	14	100

Tab. S69 nUPLC-ESI-MS-based protein identification for telithromycin. Protein names were taken from SubtiWiki database[1]. Superscripts indicate that the protein was identified in multiple spots on the gel image (see Fig. S32).

protein	protein name	MW [Da]	pI	PLGS score	peptides	coverage [%]
AdcA	zinc transport system zinc-binding lipoprotein	35637	5.2	16660	45	78
Adk	adenylate kinase	24104	4.5	6039	27	69
DivIVA	septum site-determining protein	19329	4.9	9565	25	68
DnaK	chaperone protein	65962	4.6	4353	46	53
Eno	enolase	46552	4.5	4481	33	64
Gid	methylenetetrahydrofolate--tRNA-(uracil-5-)-methyltransferase	48032	5.8	1774	25	76
IolS	aldo/keto reductase family protein	35146	5.4	4916	31	69
MetE[1]	5-methyltetrahydropteroyltriglutamate-homocysteine methyltransferase	86752	4.9	1036	23	35
MetE[2]				5632	66	68
MetE[3]				9199	98	67
PdhD	dihydrolipoyl dehydrogenase	49701	4.8	5631	40	67
Pgk	phosphoglycerate kinase	42163	4.8	4646	26	54
PupG	purine nucleoside phosphorylase	29108	4.9	1891	26	78
PyrAA	carbamoyl-phosphate synthase pyrimidine-specific small chain	40093	5.8	5049	34	45
RplJ	50S ribosomal protein L10	18067	5.2	23050	53	92
SufD	FeS cluster assembly protein	48263	5.0	17771	57	82
TpiA	triosephosphate isomerase	27012	4.8	13240	27	90
TufA[1]	elongation factor Tu	43565	4.7	24283	85	91
TufA[2]				3607	38	58
YceE	hypothetical protein	20935	4.4	6790	7	46
YhdK	anti-sigma-M factor	10634	8.7	659	5	93
YitJ	bifunctional homocysteine S-methyltransferase/5,10-methylenetetrahydrofolate reductase	67885	5.3	1972	40	79
YqfS	endonuclease IV	33047	5.4	1272	8	35
YrpE	zinc-binding protein	28851	5.1	254	17	64

Tab. S70 nUPLC-ESI-MS-based protein identification for tunicamycin. Protein names were taken from SubtiWiki database[1]. Superscripts indicate that the protein was identified in multiple spots on the gel image (see Fig. S33).

protein	protein name	MW [Da]	pI	PLGS score	peptides	coverage [%]
AhpC	alkyl hydroperoxide reductase subunit C	20614	4.3	29880	72	100
AhpF[1]	NADH dehydrogenase	54389	4.7	4262	47	66
AhpF[2]				7282	90	75
CysC	adenylyl-sulfate kinase	22530	5.1	14681	41	65
DhbB	isochorismatase	35084	4.4	3730	38	80
GuaB	inosine-5'-monophosphate dehydrogenase	52957	6.2	1711	25	48
Hag	flagellin	32606	4.8	9198	23	51
MntA	manganese-binding lipoprotein	33397	6.2	1993	16	42
NagBB	glucosamine-6-phosphate deaminase 2	27271	5.8	3458	23	49
PspA	phage shock protein A homolog	25125	5.8	1309	15	55
RacX	amino acid racemase	25269	5.4	6137	20	49
TasA	spore coat protein N	28287	5.4	2285	22	56
YceC	stress response protein SCP2	21809	5.3	7867	39	81
YdbD	manganese catalase	30238	4.9	3787	12	42
YgxA	hypothetical protein	34374	5.8	1957	28	67
YqiG	NADH-dependent flavin oxidoreductase	40779	5.2	5018	44	64
YrhB	cystathionine gamma-lyase	40860	5.2	2578	26	86
YugK	NADH-dependent butanol dehydrogenase 2	43378	4.6	2970	39	61
YvlB	hypothetical protein	41055	5.4	5191	27	52

Tab. S71 nUPLC-ESI-MS-based protein identification for UC41. Protein names were taken from SubtiWiki database[1].

protein	protein name	MW [Da]	pI	PLGS score	peptides	coverage [%]
ClpC	AAA unflodase, ATPase subunit of the ClpC-CplP protease	90063	5.7	11593	162	78
FosB	fosfomycin resistance protein	17161	6.1	2240	18	77
HisS	histidyl-tRNA synthetase	48101	5.2	24996	121	89
NfrA	FMN-containing NADPH-linked nitro/flavin reductase	28302	5.7	20181	34	63
PbpE	penicillin-binding Protein PBP4* (spore cortex)	51404	4.8	16515	92	87
PdhB	pyruvate dehydrogenase (E1 beta subunit)	35452	4.5	2457	22	45
PspA	phage shock protein A homolog	25125	5.8	13563	91	86
RacX	amino acid racemase	25269	5.4	12339	37	31
RplL	ribosomale protein L12	12742	4.3	144653	114	94
SalA	MRP family regulator	38614	5.2	10282	52	56
SodA	superoxide dismutase	22476	5.1	19007	23	84
YceH	unknown	41646	5.7	15255	157	85
YdgG	unknown	17263	5.6	10876	15	74
YdjP	unknown	30489	4.6	11487	33	56
YfhM	unknown	32737	6.1	23449	64	78
YhfK	unknown	22745	5.2	51576	75	92
YjbC	general stress protein	23105	5.1	13172	51	73
YjbL	unknown	15106	5.2	2710	17	95
YlbA	unknown	13484	5.0	17399	26	84
YojG	N-acetylglucosamin-malate deacetylase	25560	4.7	1045	13	30
YpqE	unknown	17924	5.3	1156	12	67
YuaG	membrane-associated scaffold protein	55959	5.1	34541	179	83
YuaI	unknown	19830	5.2	17957	23	91
YukJ	unknown	25538	4.4	6418	31	98
YvbH	unknown	23787	4.9	6136	18	59
YvlB	unknown	41055	5.4	11859	52	84
YwrO	unknown	19942	5.2	16233	24	62
YycD	unknown	7456	4.8	3430	4	76

Supplementary figures

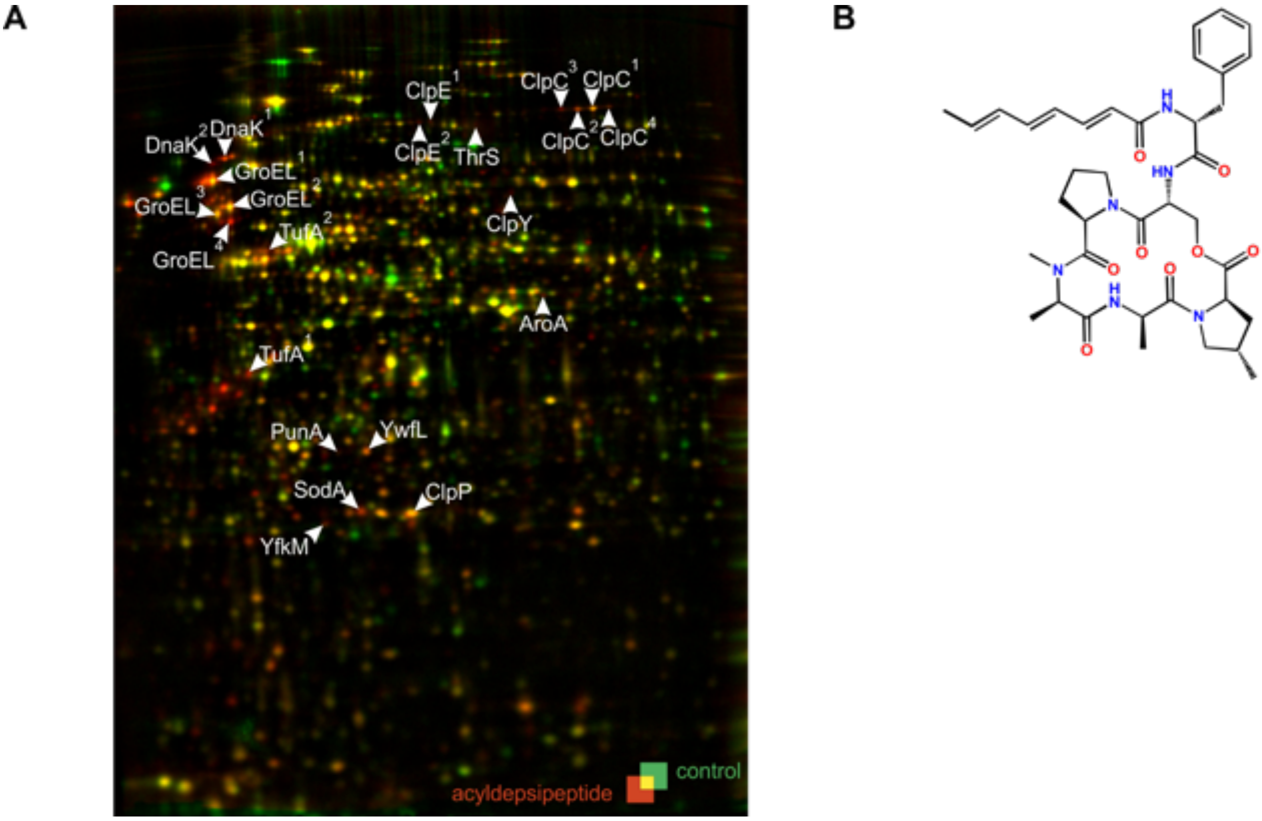

Fig. S1 Proteomic response of *B. subtilis* towards acyldepsipeptide. (A) Identified marker proteins based on 2D-PAGE analysis of cytosolic proteins of *B. subtilis* treated with 1 µg/ml of acyldepsipeptide (B). Superscripts indicate when a protein was identified in multiple spots. Synthesis of cytosolic proteins of untreated (green) and antibiotic-treated (red) *B. subtilis* were compared based on incorporation of radioactive [^{35}S]-methionine. Data taken from Brötz-Oesterhelt *et al*., 2005[4]. Proteins were identified based on comparison with Eymann *et al*., 2004[5]. Acyldepsipeptide binds ClpP and enables uncontrolled proteolysis in the absence of a regulatory Clp-ATPase subunit[4]. All sections show representative data.

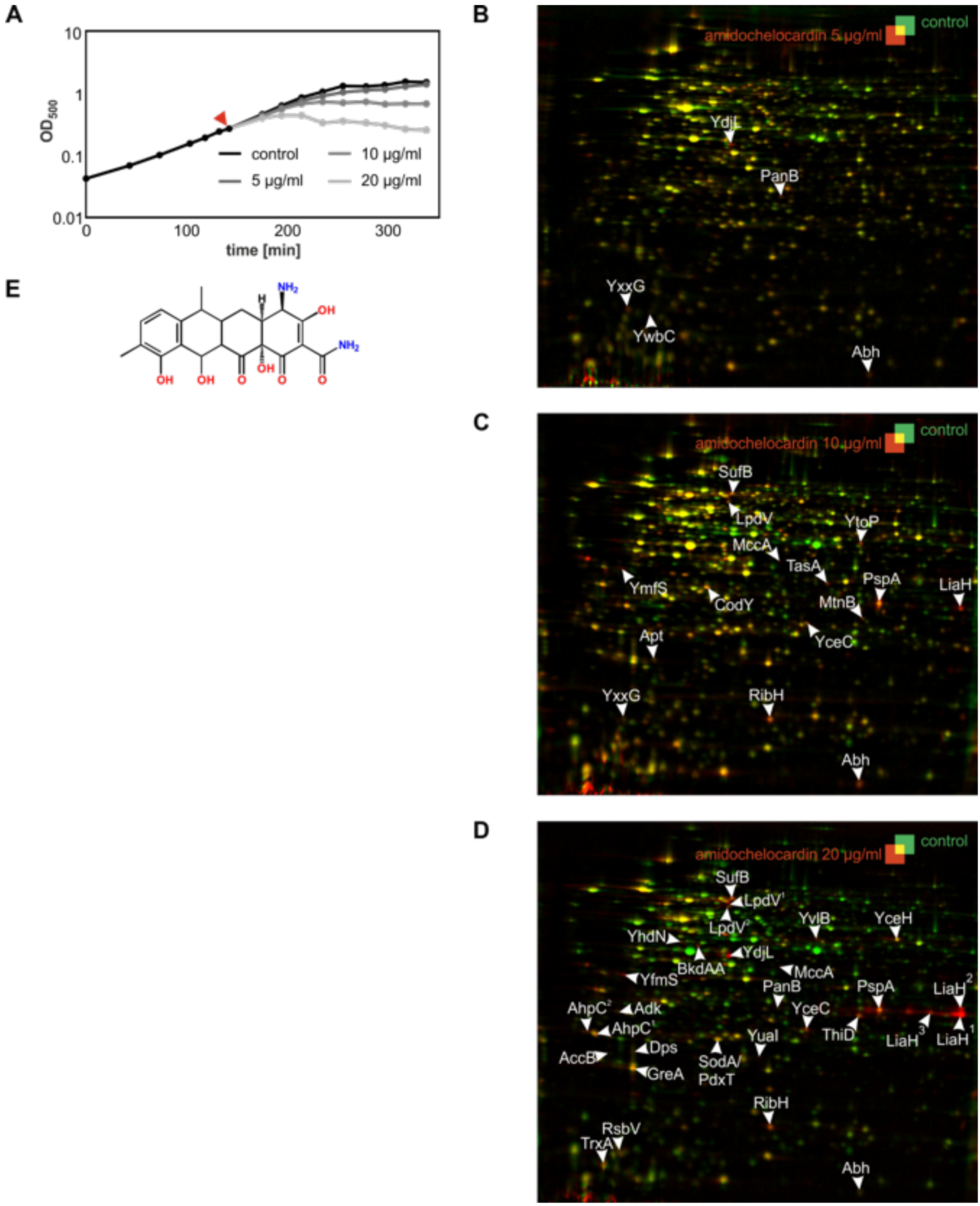

Fig. S2 Proteomic response of *B. subtilis* towards amidochelocardin. (A) growth curves of *B. subtilis*, comparing an untreated control with cultures treated with 2-carboxamido-2-deacetyl-chelochardin (amidochelocardin). Identified marker proteins based on 2D-PAGE analysis of cytosolic proteins of *B. subtilis* treated with 5 µg/ml (B), 10 µg/ml (C), and 20 µg/ml (D) of amidochelocardin (E). Superscripts indicate when a protein was identified in multiple spots. Synthesis of cytosolic proteins of untreated (green) and antibiotic-treated (red) *B. subtilis* were compared based on incorporation of radioactive [^{35}S]-methionine. By applying CoPR approach, we have gained strong indication that the atypical tetracycline amidochelocardin concentration-dependently targets protein synthesis and/or the cytoplasmic membrane. All sections show representative data.

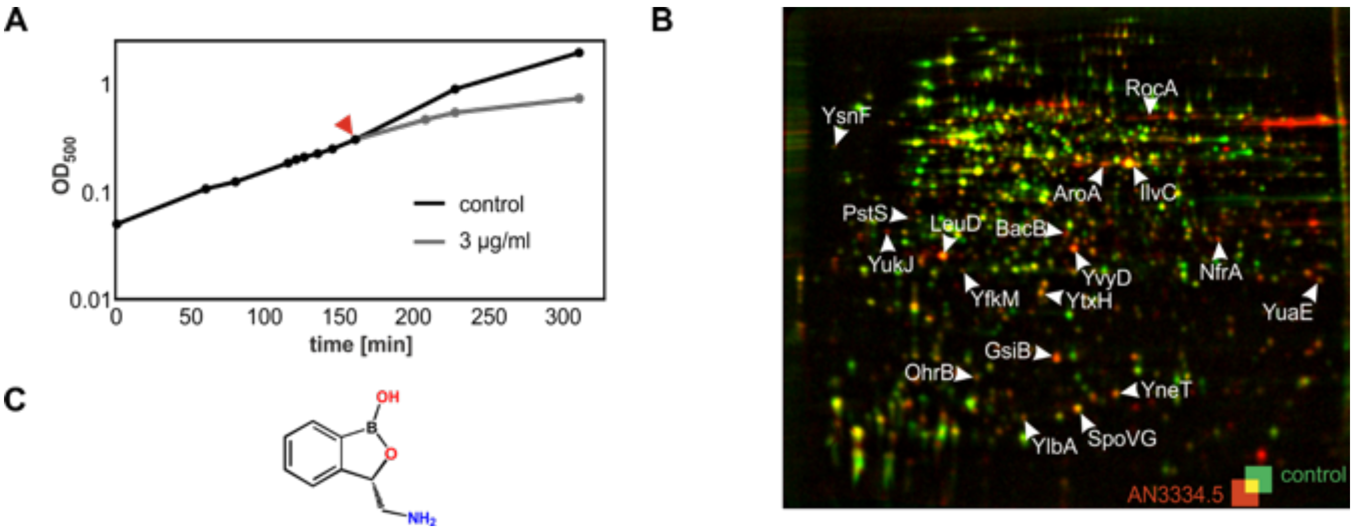

Fig. S3 Proteomic response of *B. subtilis* towards AN3334. (A) growth curves of *B. subtilis*, comparing an untreated control with a culture treated with AN3334. Identified marker proteins based on 2D-PAGE analysis of cytosolic proteins of *B. subtilis* treated with 3 µg/ml (B) of AN3334 (C). Synthesis of cytosolic proteins of untreated (green) and antibiotic-treated (red) *B. subtilis* were compared based on incorporation of radioactive [^{35}S]-methionine. AN3334 inhibits the bacterial leucyl-tRNA synthetase[6]. All sections show representative data.

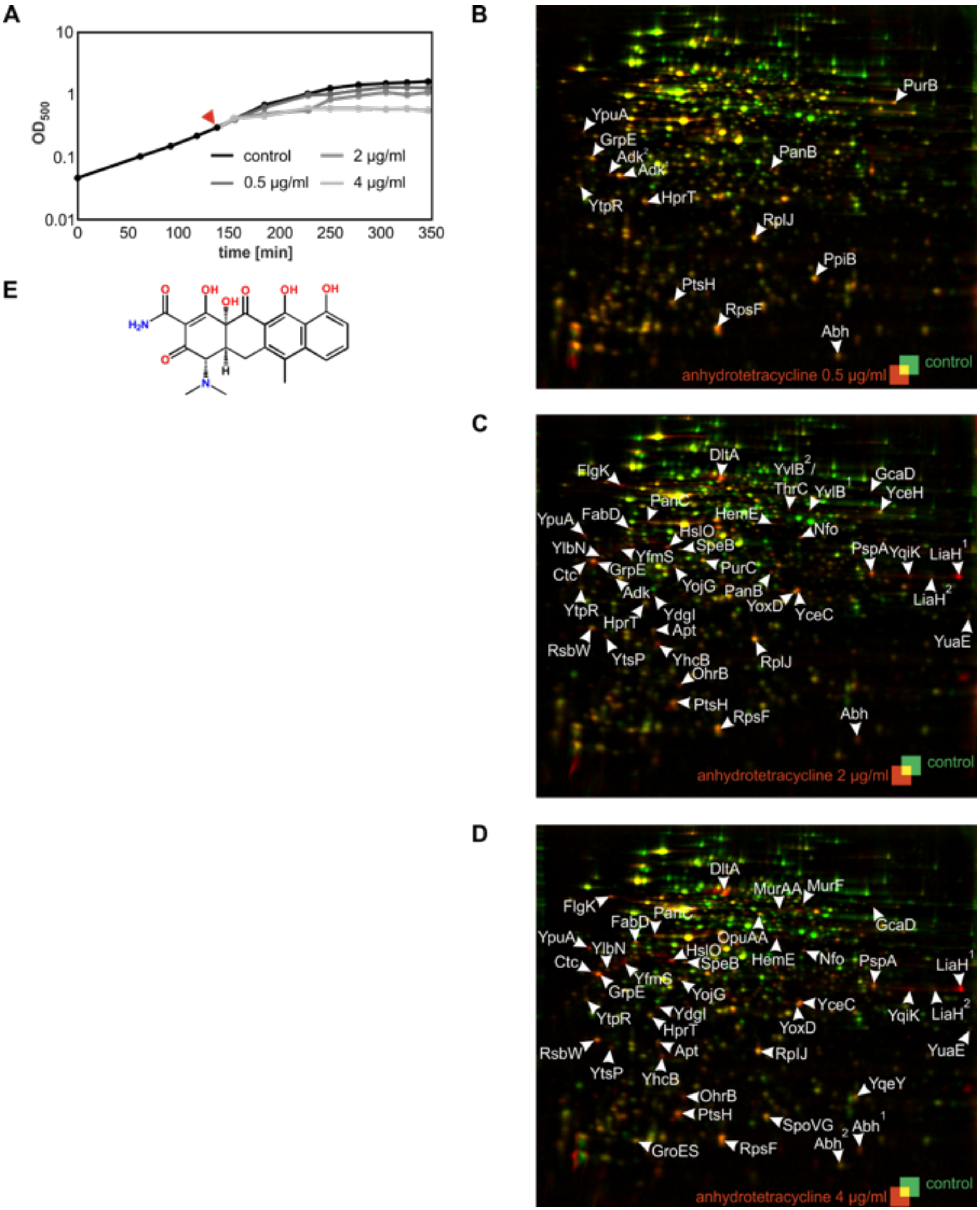

Fig. S4 Proteomic response of *B. subtilis* towards anhydrotetracycline. (A) growth curves of *B. subtilis*, comparing an untreated control with cultures treated with anhydrotetracycline. Identified marker proteins based on 2D-PAGE analysis of cytosolic proteins of *B. subtilis* treated with 0.5 µg/ml (B), 2 µg/ml (C), and 4 µg/ml (D) of anhydrotetracycline (E). Superscripts indicate when a protein was identified in multiple spots. Synthesis of cytosolic proteins of untreated (green) and antibiotic-treated (red) *B. subtilis* were compared based on incorporation of radioactive [^{35}S]-methionine. Anhydrotetracycline concentration-dependently targets protein synthesis and/or the cytoplasmic membrane[7]. All sections show representative data.

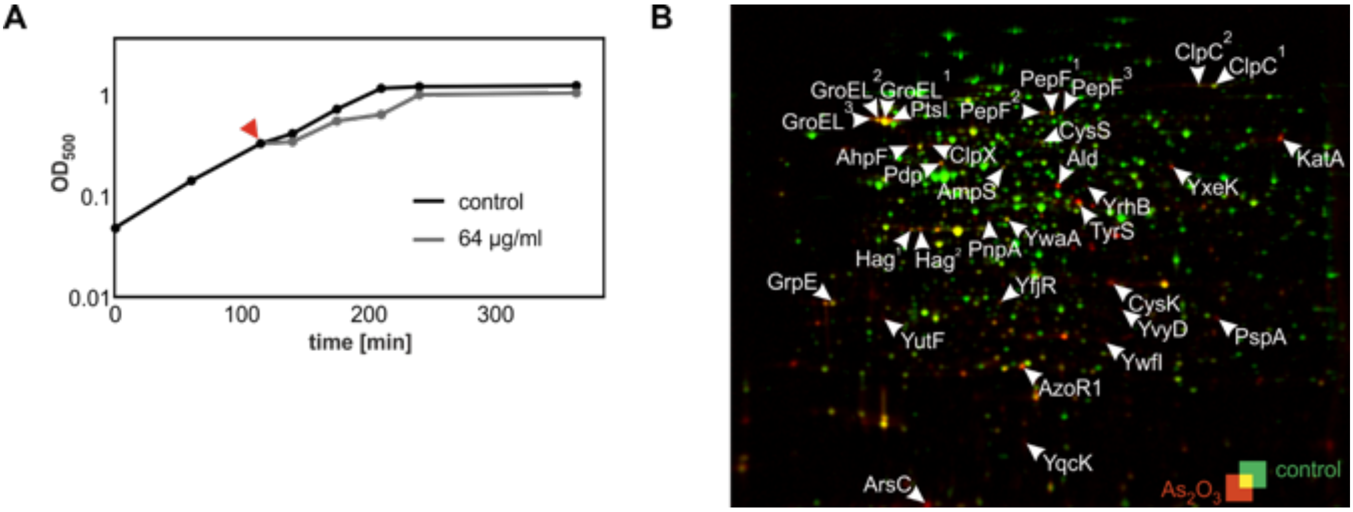

Fig. S5 Proteomic response of *B. subtilis* towards As_2O_3. (A) growth curves of *B. subtilis*, comparing an untreated control with a culture treated with As_2O_3. Identified marker proteins based on 2D-PAGE analysis of cytosolic proteins of *B. subtilis* treated with 64 µg/ml (B) of As_2O_3. Superscripts indicate when a protein was identified in multiple spots. Synthesis of cytosolic proteins of untreated (green) and antibiotic-treated (red) *B. subtilis* were compared based on incorporation of radioactive $[^{35}S]$-methionine. Arsenic reacts with thiols[8]. All sections show representative data.

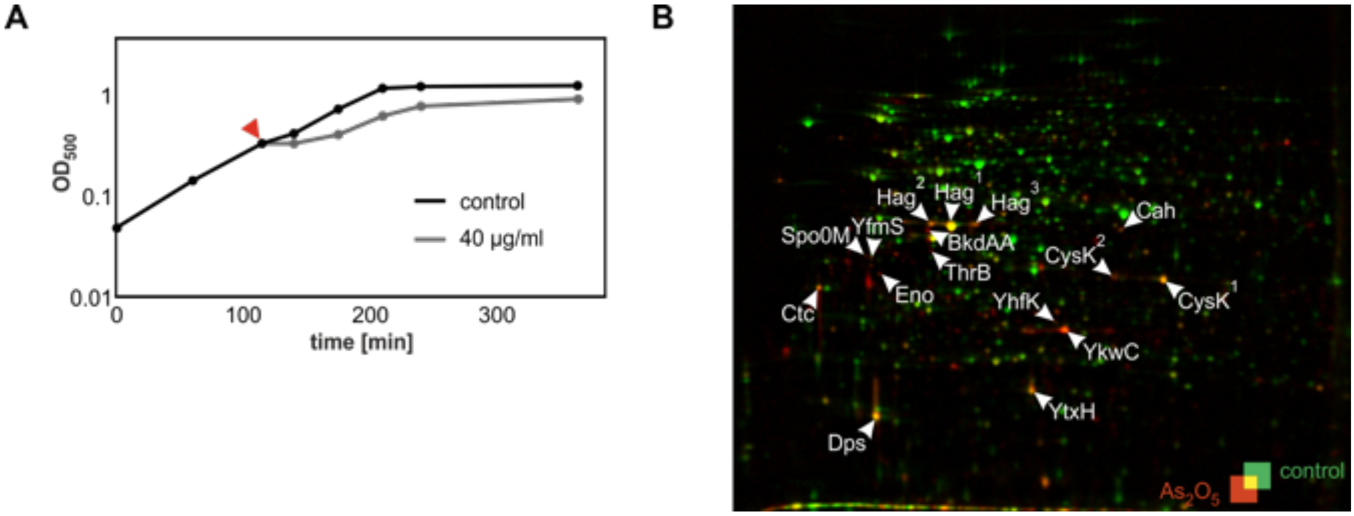

Fig. S6 Proteomic response of *B. subtilis* towards As_2O_5. (A) growth curves of *B. subtilis*, comparing an untreated control with a culture treated with As_2O_5. Identified marker proteins based on 2D-PAGE analysis of cytosolic proteins of *B. subtilis* treated with 40 µg/ml (B) of As_2O_5. Superscripts indicate when a protein was identified in multiple spots. Synthesis of cytosolic proteins of untreated (green) and antibiotic-treated (red) *B. subtilis* were compared based on incorporation of radioactive $[^{35}S]$-methionine. Arsenic reacts with thiols[8]. All sections show representative data.

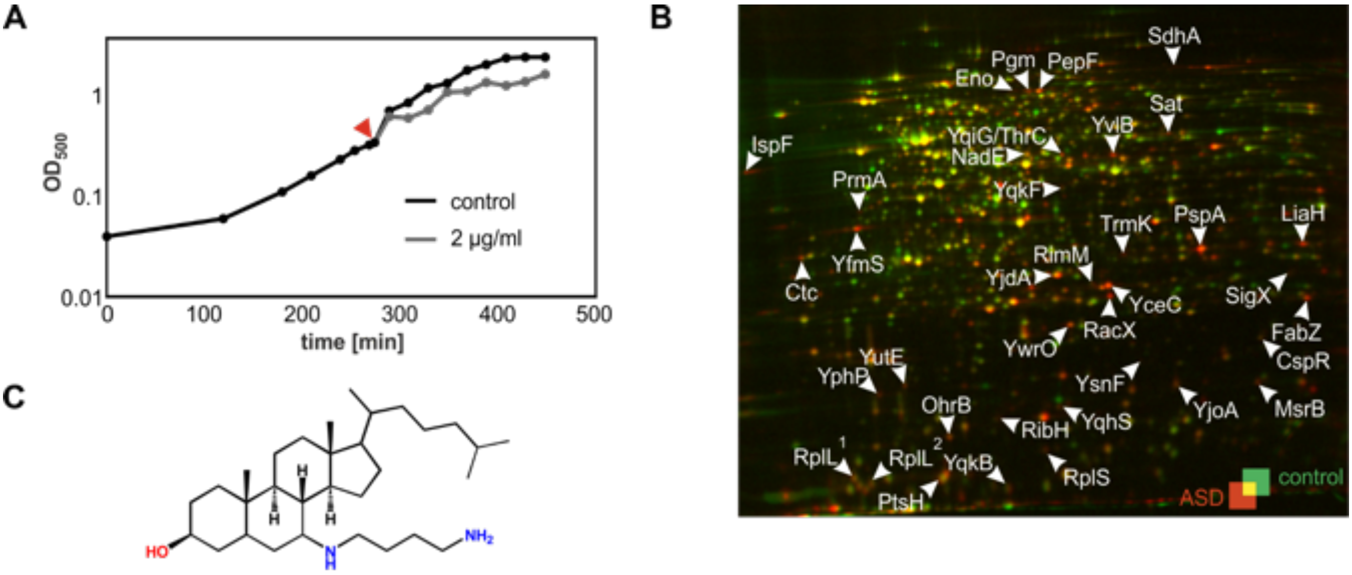

Fig. S7 Proteomic response of *B. subtilis* towards ASD. (A) growth curves of *B. subtilis*, comparing an untreated control with a culture treated with ASD. Identified marker proteins based on 2D-PAGE analysis of cytosolic proteins of *B. subtilis* treated with 2 µg/ml (B) of ASD (C). Superscripts indicate when a protein was identified in multiple spots. Synthesis of cytosolic proteins of untreated (green) and antibiotic-treated (red) *B. subtilis* were compared based on incorporation of radioactive [^{35}S]-methionine. Squalamine-like compounds disturb bacterial membranes[9]. All sections show representative data.

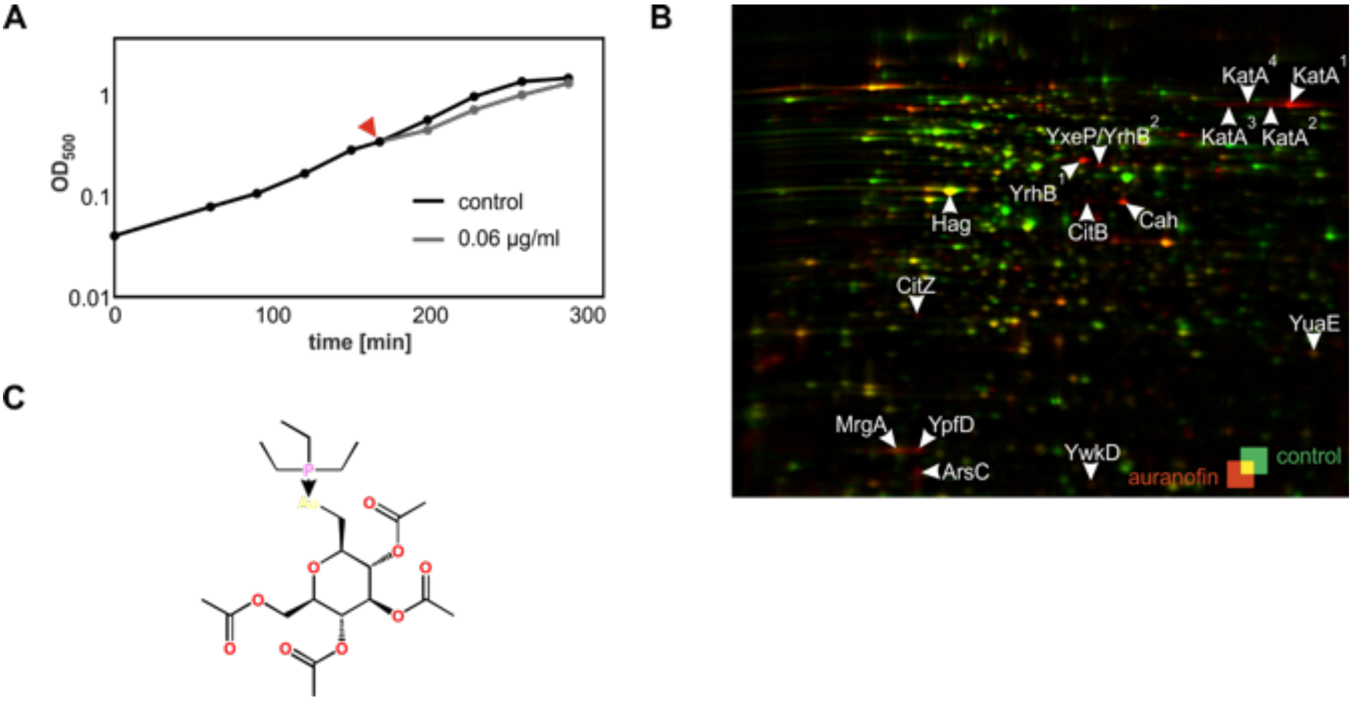

Fig. S8 Proteomic response of *B. subtilis* towards auranofin. (A) growth curves of *B. subtilis*, comparing an untreated control with a culture treated with auranofin. Identified marker proteins based on 2D-PAGE analysis of cytosolic proteins of *B. subtilis* treated with 0.06 µg/ml (B) of auranofin (C). Superscripts indicate when a protein was identified in multiple spots. Synthesis of cytosolic proteins of untreated (green) and antibiotic-treated (red) *B. subtilis* were compared based on incorporation of radioactive [^{35}S]-methionine. Auranofin impairs the thiol-redox homeostasis of bacteria[10]. All sections show representative data.

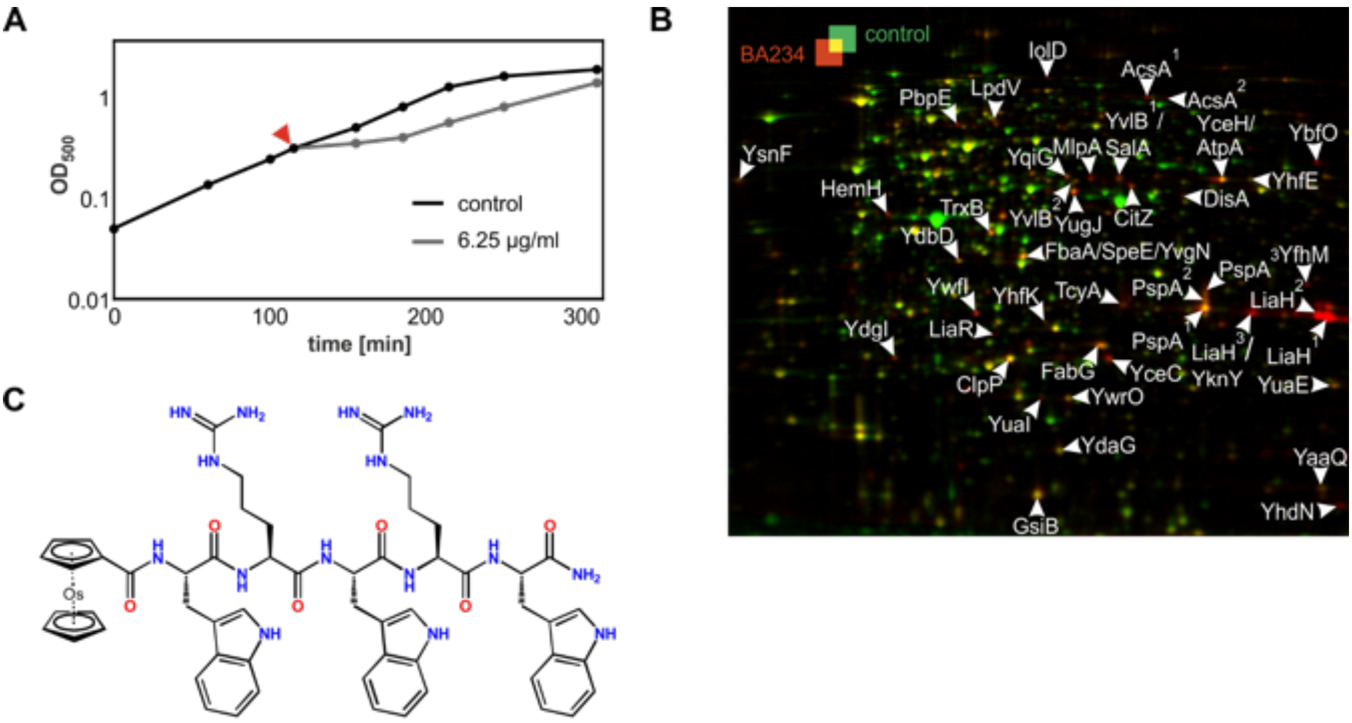

Fig. S9 Proteomic response of *B. subtilis* towards BA234. (A) growth curves of *B. subtilis*, comparing an untreated control with a culture treated with BA234. Identified marker proteins based on 2D-PAGE analysis of cytosolic proteins of *B. subtilis* treated with 6.25 µg/ml (B) of BA234 (C). Superscripts indicate when a protein was identified in multiple spots. Synthesis of cytosolic proteins of untreated (green) and antibiotic-treated (red) *B. subtilis* were compared based on incorporation of radioactive [^{35}S]-methionine. BA234 distrubes the bacterial membrane. All sections show representative data.

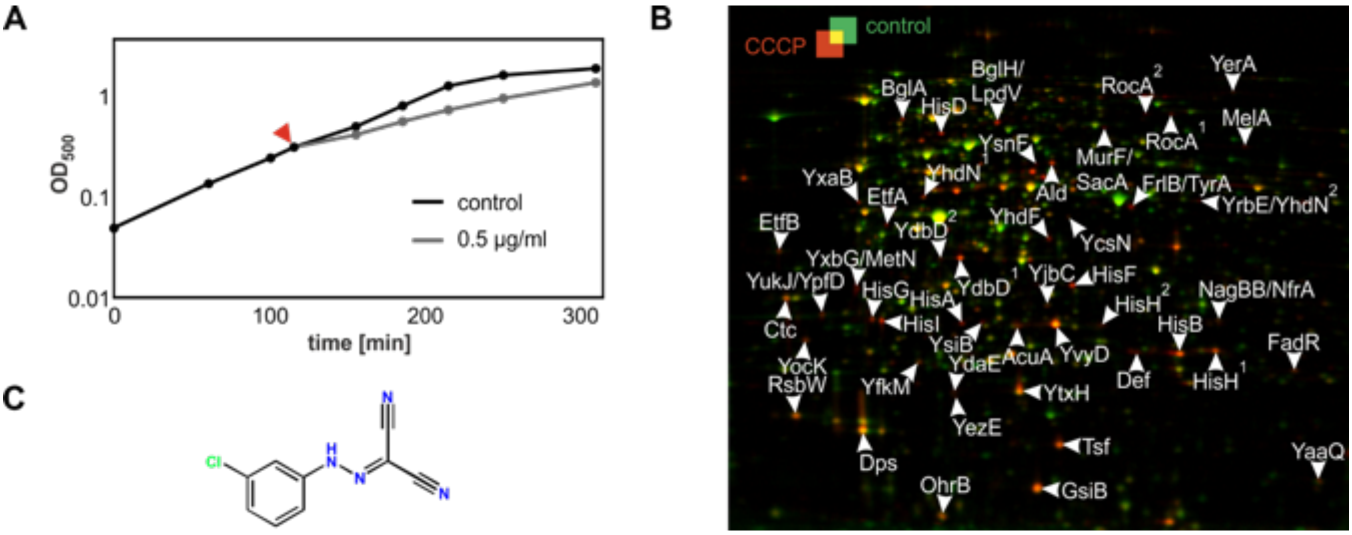

Fig. S10 Proteomic response of *B. subtilis* towards CCCP. (A) growth curves of *B. subtilis*, comparing an untreated control with a culture treated with CCCP. Identified marker proteins based on 2D-PAGE analysis of cytosolic proteins of *B. subtilis* treated with 0.5 µg/ml (B) of CCCP (C). Superscripts indicate when a protein was identified in multiple spots. Synthesis of cytosolic proteins of untreated (green) and antibiotic-treated (red) *B. subtilis* were compared based on incorporation of radioactive [^{35}S]-methionine. CCCP is a protonophore and disturbes the respiratory chain[11,12]. All sections show representative data.

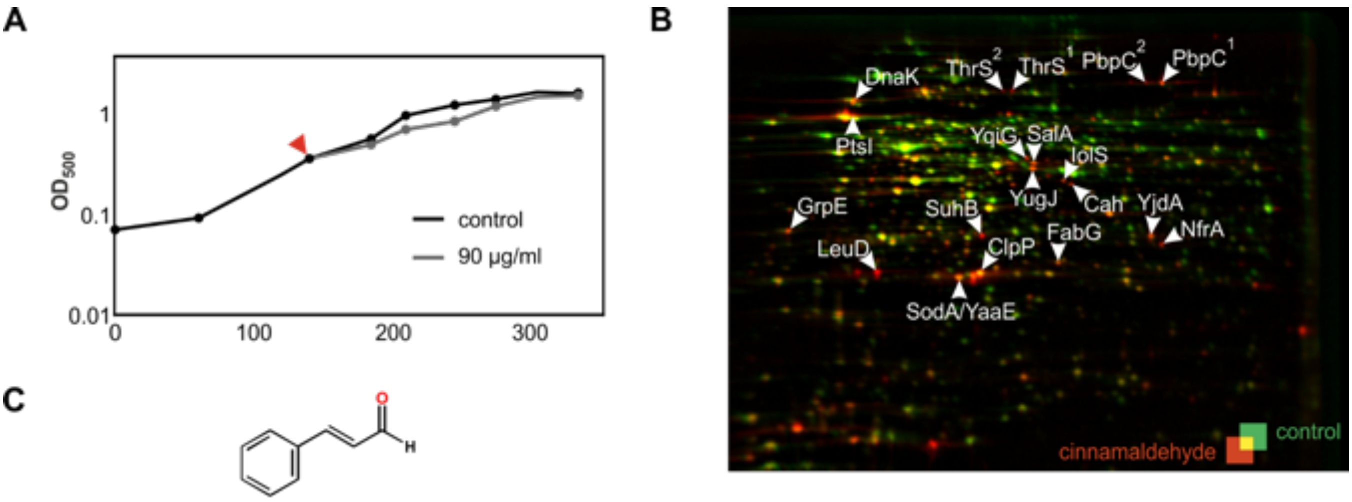

Fig. S11 Proteomic response of *B. subtilis* towards cinnamaldehyde. (A) growth curves of *B. subtilis*, comparing an untreated control with a culture treated with cinnamaldehyde. Identified marker proteins based on 2D-PAGE analysis of cytosolic proteins of *B. subtilis* treated with 90 µg/ml (B) of cinnamaldehyde (C). Superscripts indicate when a protein was identified in multiple spots. Synthesis of cytosolic proteins of untreated (green) and antibiotic-treated (red) *B. subtilis* were compared based on incorporation of radioactive [^{35}S]-methionine. Cinnamaldehyde inhibits FtsZ, permeabilizes the membrane and reacts with thiols[13-15]. All sections show representative data.

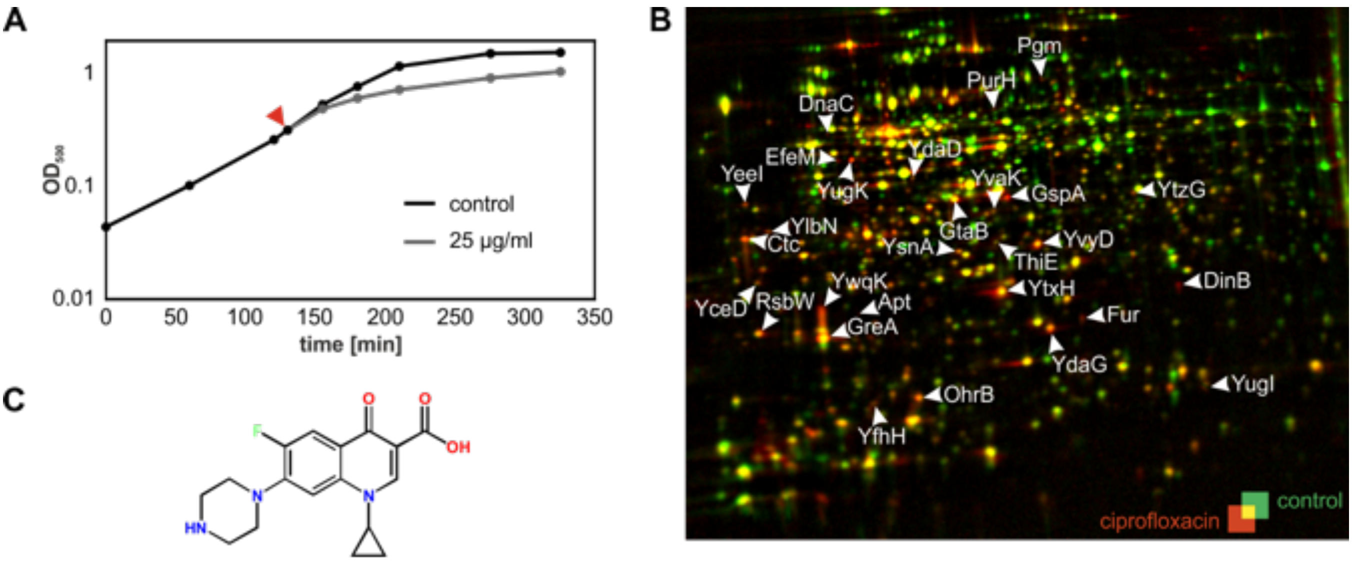

Fig. S12 Proteomic response of *B. subtilis* towards ciprofloxacin. (A) growth curves of *B. subtilis*, comparing an untreated control with a culture treated with ciprofloxacin. Identified marker proteins based on 2D-PAGE analysis of cytosolic proteins of *B. subtilis* treated with 25 µg/ml (B) of ciprofloxacin (C). Synthesis of cytosolic proteins of untreated (green) and antibiotic-treated (red) *B. subtilis* were compared based on incorporation of radioactive [^{35}S]-methionine. Ciprofloxacin inhibits DNA gyrase and topoisomerase IV[16-18]. All sections show representative data.

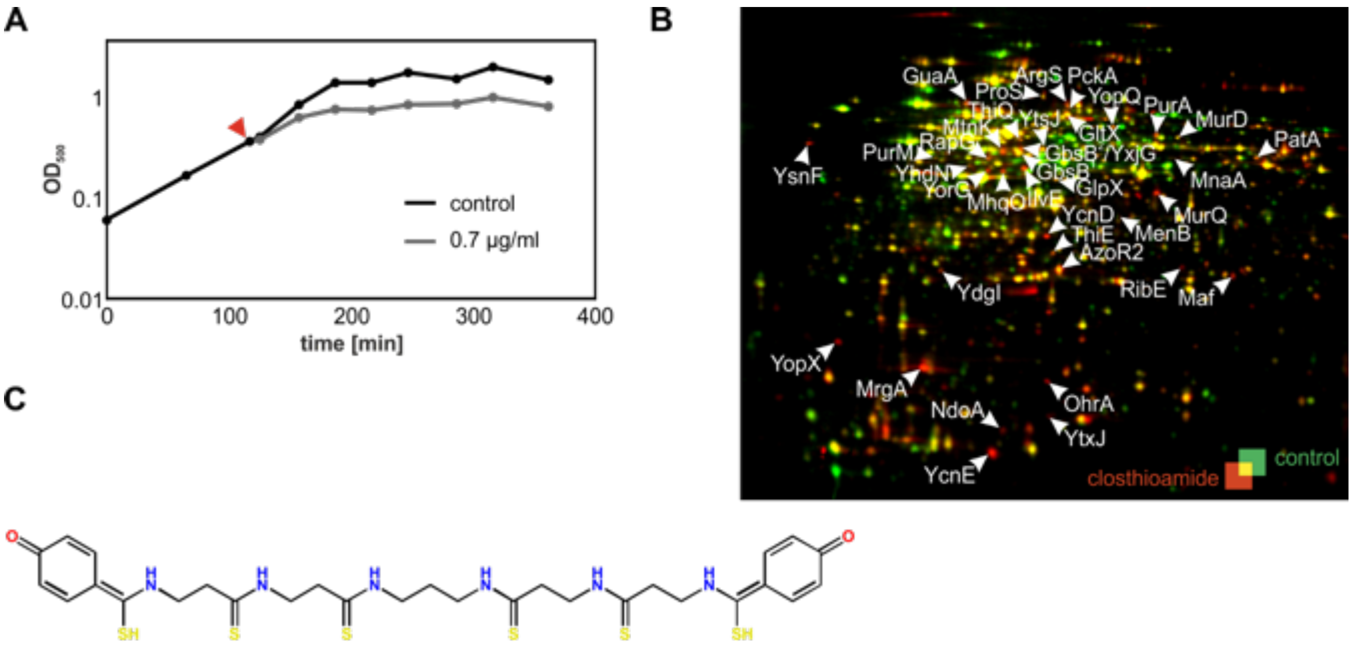

Fig. S13 Proteomic response of *B. subtilis* towards closthioamide. (A) growth curves of *B. subtilis*, comparing an untreated control with a culture treated with closthioamide. Identified marker proteins based on 2D-PAGE analysis of cytosolic proteins of *B. subtilis* treated with 0.7 µg/ml (B) of closthioamide (C). Superscripts indicate when a protein was identified in multiple spots. Synthesis of cytosolic proteins of untreated (green) and antibiotic-treated (red) *B. subtilis* were compared based on incorporation of radioactive [^{35}S]-methionine. Closthioamide inhibits DNA gyrase and topoisomerase IV[19]. All sections show representative data.

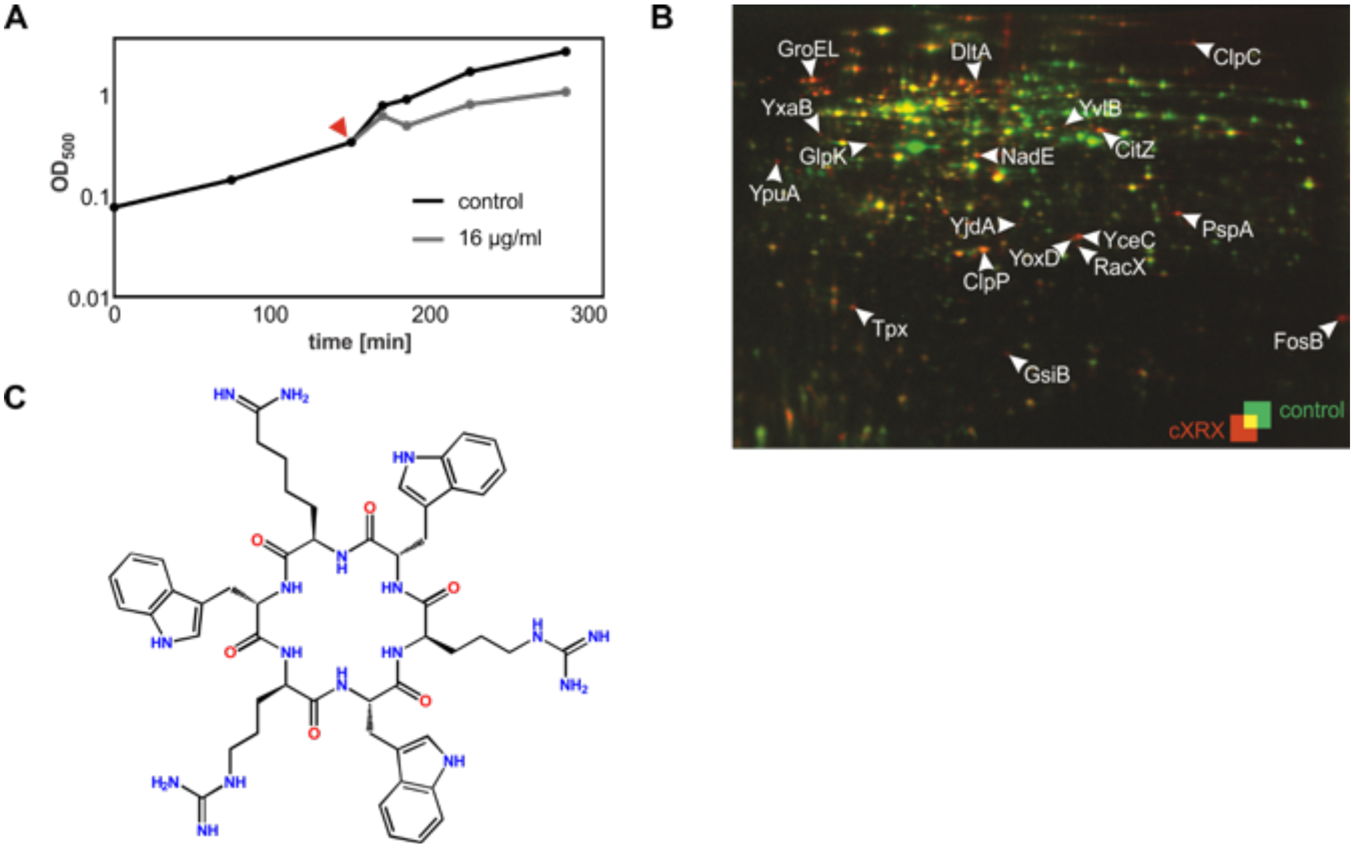

Fig. S14 Proteomic response of *B. subtilis* towards cXRX. (A) growth curves of *B. subtilis*, comparing an untreated control with a culture treated with cXRX. Identified marker proteins based on 2D-PAGE analysis of cytosolic proteins of *B. subtilis* treated with 16 µg/ml (B) of cXRX (C). Synthesis of cytosolic proteins of untreated (green) and antibiotic-treated (red) *B. subtilis* were compared based on incorporation of radioactive [^{35}S]-methionine. cXRX disturbes the bacterial membrane. All sections show representative data.

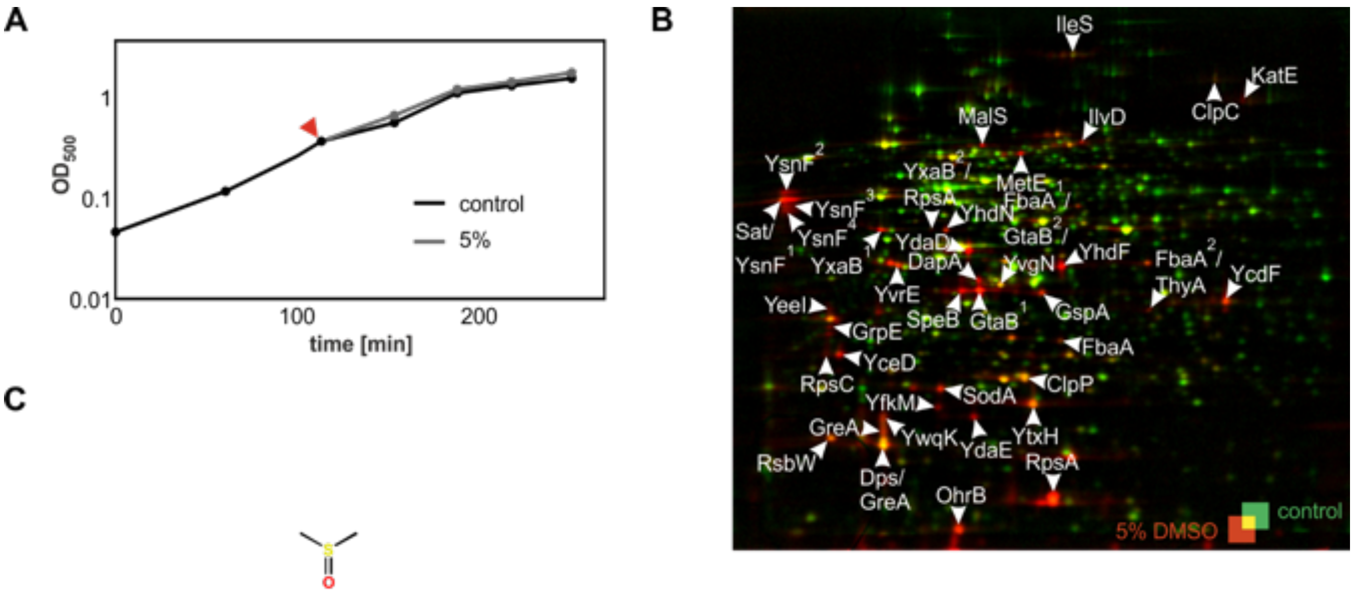

Fig. S15 Proteomic response of *B. subtilis* towards 5% DMSO. (A) growth curves of *B. subtilis*, comparing an untreated control with a culture treated with DMSO. Identified marker proteins based on 2D-PAGE analysis of cytosolic proteins of *B. subtilis* treated with 5% (B) of DMSO (C). Synthesis of cytosolic proteins of untreated (green) and solvent-treated (red) *B. subtilis* were compared based on incorporation of radioactive [^{35}S]-methionine. All sections show representative data.

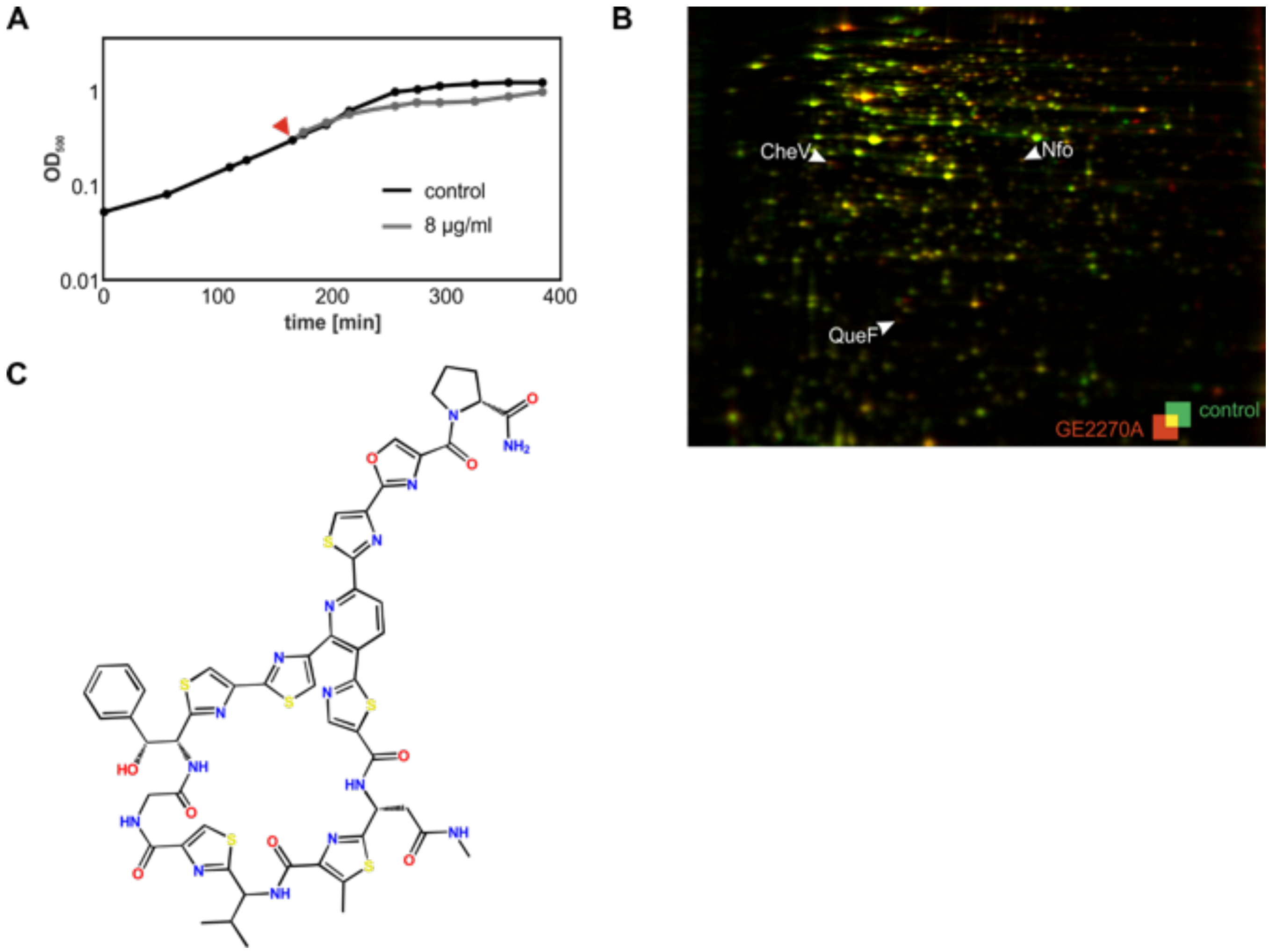

Fig. S16 Proteomic response of *B. subtilis* towards GE2270A. (A) growth curves of *B. subtilis*, comparing an untreated control with a culture treated with GE2270A. Identified marker proteins based on 2D-PAGE analysis of cytosolic proteins of *B. subtilis* treated with 8 µg/ml (B) of GE2270A (C). Synthesis of cytosolic proteins of untreated (green) and antibiotic-treated (red) *B. subtilis* were compared based on incorporation of radioactive [^{35}S]-methionine. GE2270A binds the elongation factor Tu[20,21]. All sections show representative data.

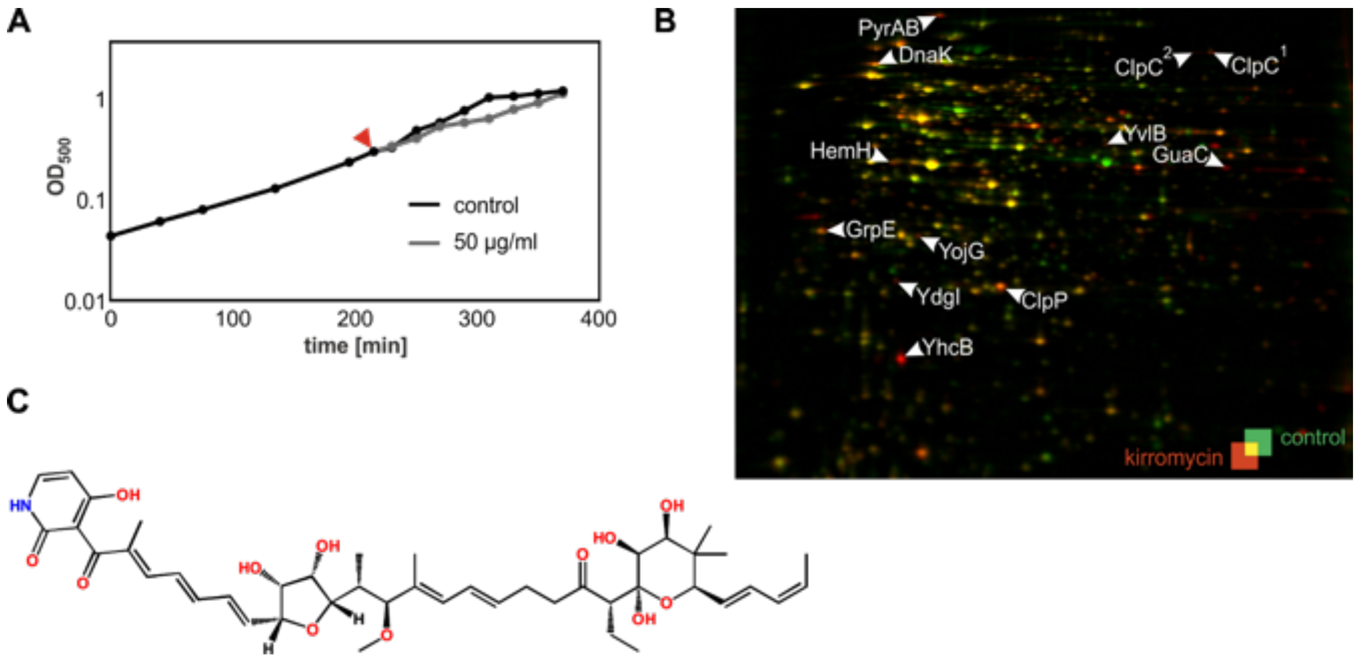

Fig. S17 Proteomic response of *B. subtilis* towards kirromycin. (A) growth curves of *B. subtilis*, comparing an untreated control with a culture treated with kirromycin. Identified marker proteins based on 2D-PAGE analysis of cytosolic proteins of *B. subtilis* treated with 50 µg/ml (B) of kirromycin (C). Superscripts indicate when a protein was identified in multiple spots. Synthesis of cytosolic proteins of untreated (green) and antibiotic-treated (red) *B. subtilis* were compared based on incorporation of radioactive [^{35}S]-methionine. Kirromycin binds the elongation factor Tu[22,23]. All sections show representative data.

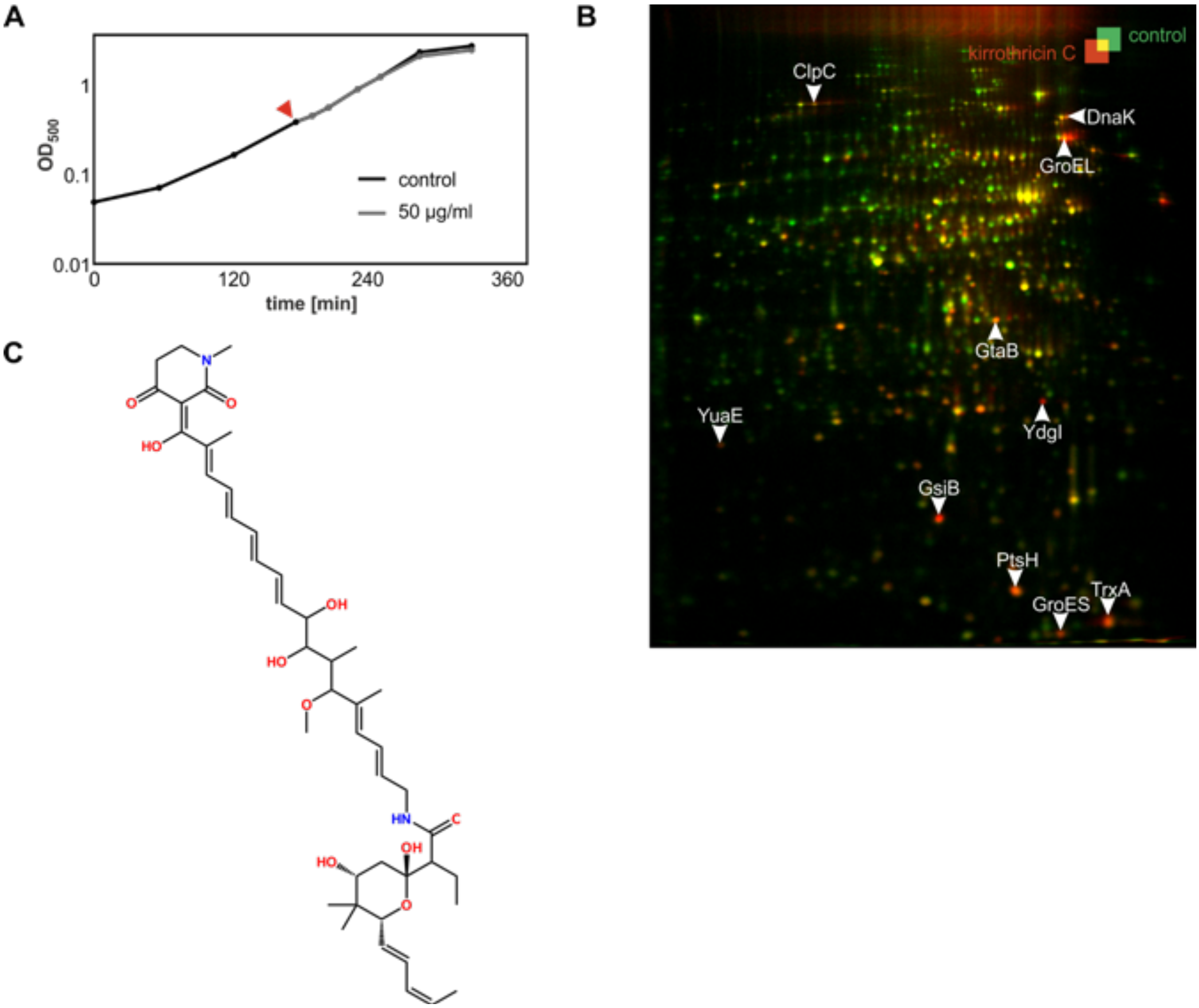

Fig. S18 Proteomic response of *B. subtilis* towards kirrothricin C. (A) growth curves of *B. subtilis*, comparing an untreated control with a culture treated with kirromycin. Identified marker proteins based on 2D-PAGE analysis of cytosolic proteins of *B. subtilis* treated with 100 µg/ml (B) of kirrothricin C (C), unlike the growth curve. Synthesis of cytosolic proteins of untreated (green) and antibiotic-treated (red) *B. subtilis* were compared based on incorporation of radioactive [^{35}S]-methionine. Kirrothricin C binds to elongation factor Tu[24]. All sections show representative data.

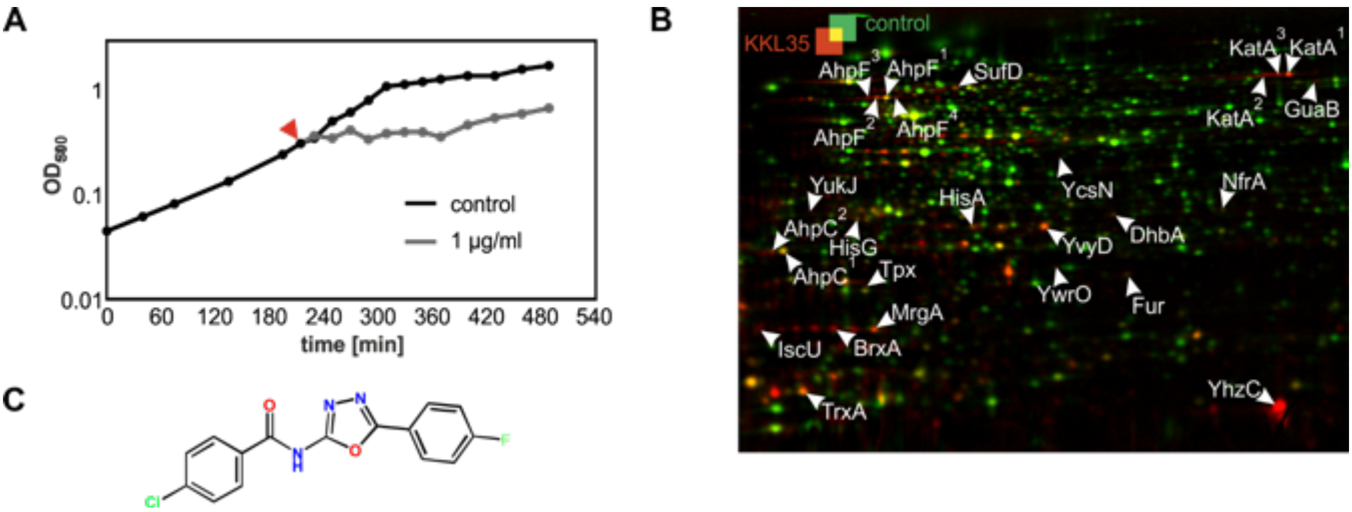

Fig. S19 Proteomic response of *B. subtilis* towards KKL-35. (A) growth curves of *B. subtilis*, comparing an untreated control with a culture treated with KKL-35. Identified marker proteins based on 2D-PAGE analysis of cytosolic proteins of *B. subtilis* treated with 1 µg/ml (B) of KKL-35 (C). Superscripts indicate when a protein was identified in multiple spots. Synthesis of cytosolic proteins of untreated (green) and antibiotic-treated (red) *B. subtilis* were compared based on incorporation of radioactive [^{35}S]-methionine. KKL-35 inhibits *trans*-translation[25]. All sections show representative data.

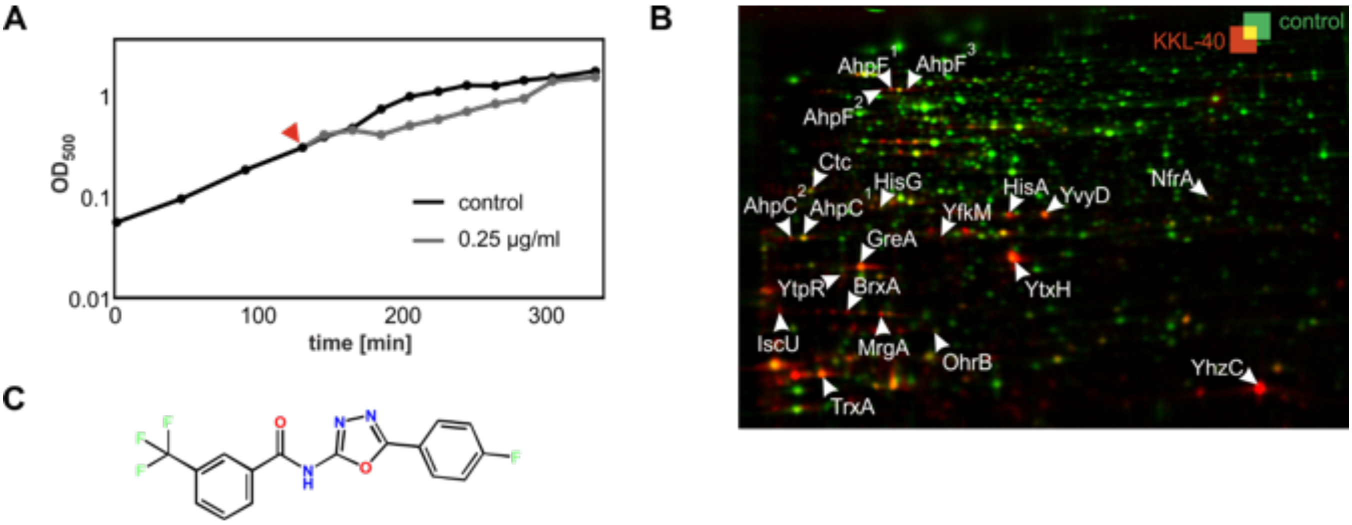

Fig. S20 Proteomic response of *B. subtilis* towards KKL-40. (A) growth curves of *B. subtilis*, comparing an untreated control with a culture treated with KKL-40. Identified marker proteins based on 2D-PAGE analysis of cytosolic proteins of *B. subtilis* treated with 0.25 µg/ml (B) of KKL-40 (C). Superscripts indicate when a protein was identified in multiple spots. Synthesis of cytosolic proteins of untreated (green) and antibiotic-treated (red) *B. subtilis* were compared based on incorporation of radioactive [^{35}S]-methionine. KKL-40 inhibits *trans*-translation[25]. All sections show representative data.

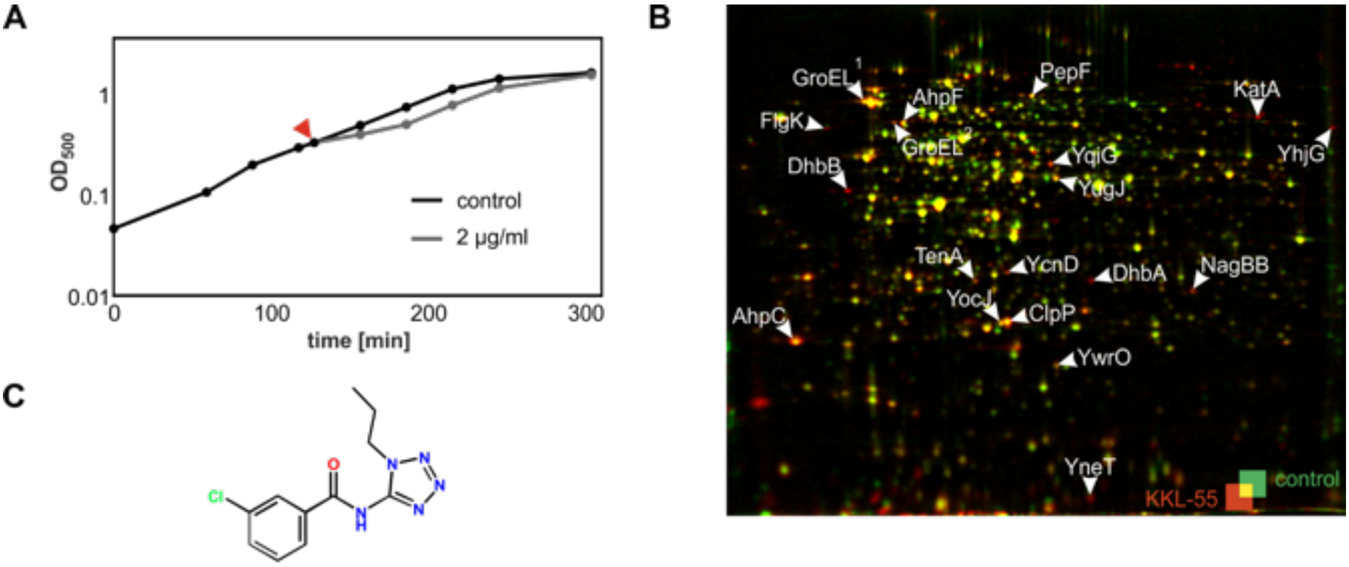

Fig. S21 Proteomic response of *B. subtilis* towards KKL-55. (A) growth curves of *B. subtilis*, comparing an untreated control with a culture treated with KKL-55. Identified marker proteins based on 2D-PAGE analysis of cytosolic proteins of *B. subtilis* treated with 2 µg/ml (B) of KKL-55 (C). Superscripts indicate when a protein was identified in multiple spots. Synthesis of cytosolic proteins of untreated (green) and antibiotic-treated (red) *B. subtilis* were compared based on incorporation of radioactive [^{35}S]-methionine. KKL-55 inhibits *trans*-translation[25]. All sections show representative data.

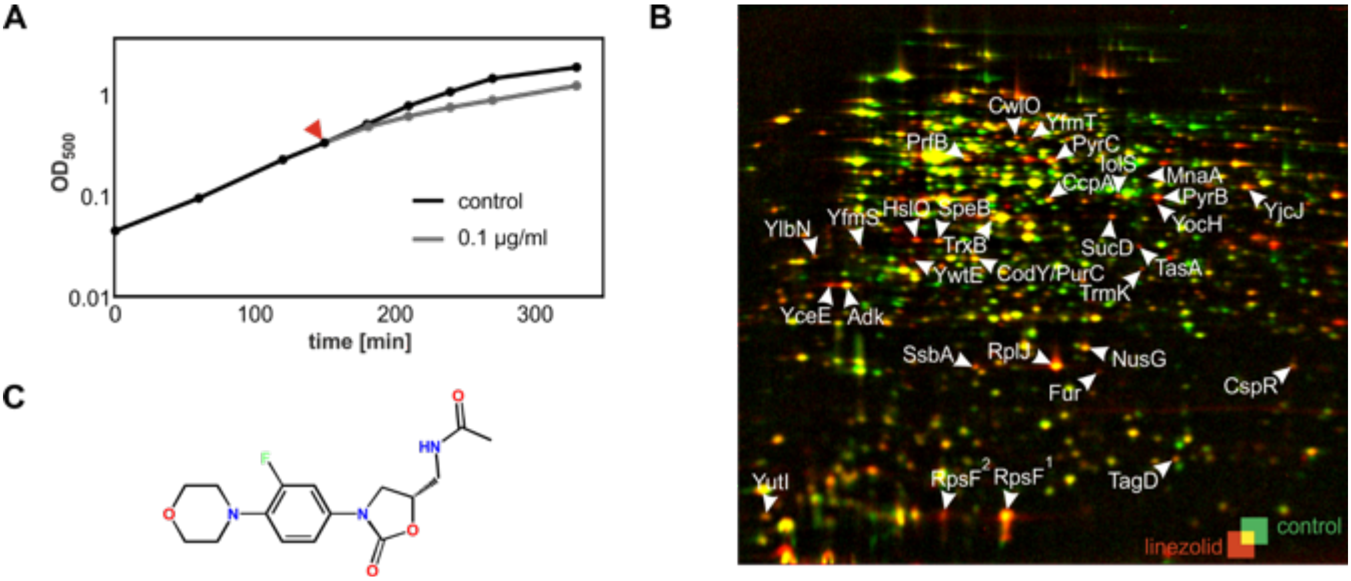

Fig. S22 Proteomic response of *B. subtilis* towards linezolid. (A) growth curves of *B. subtilis*, comparing an untreated control with a culture treated with linezolid. Identified marker proteins based on 2D-PAGE analysis of cytosolic proteins of *B. subtilis* treated with 0.1 µg/ml (B) of linezolid (C). Superscripts indicate when a protein was identified in multiple spots. Synthesis of cytosolic proteins of untreated (green) and antibiotic-treated (red) *B. subtilis* were compared based on incorporation of radioactive [^{35}S]-methionine. Linezolid inhibits protein synthesis[26,27]. All sections show representative data.

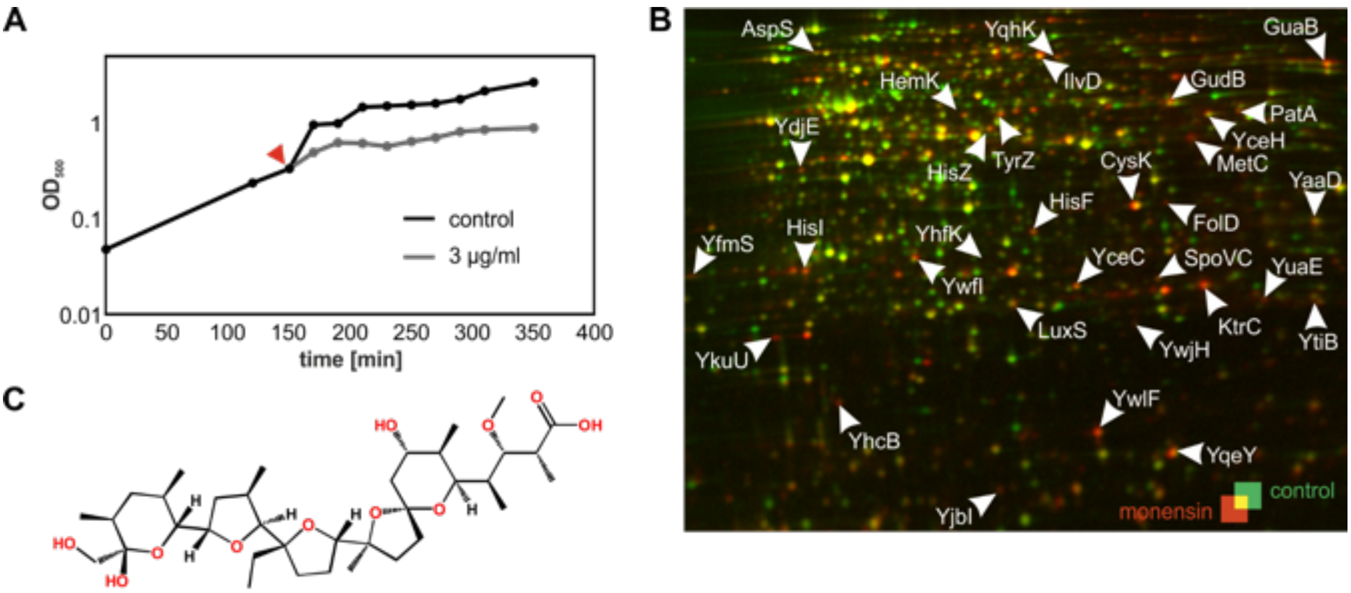

Fig. S23 Proteomic response of *B. subtilis* towards monensin. (A) growth curves of *B. subtilis*, comparing an untreated control with a culture treated with monensin. Identified marker proteins based on 2D-PAGE analysis of cytosolic proteins of *B. subtilis* treated with 3 µg/ml (B) of monensin (C). Synthesis of cytosolic proteins of untreated (green) and antibiotic-treated (red) *B. subtilis* were compared based on incorporation of radioactive $[^{35}S]$-methionine. Monensin is an ionophor, which transports monovalent cations over biological membranes2[8,29]. All sections show representative data.

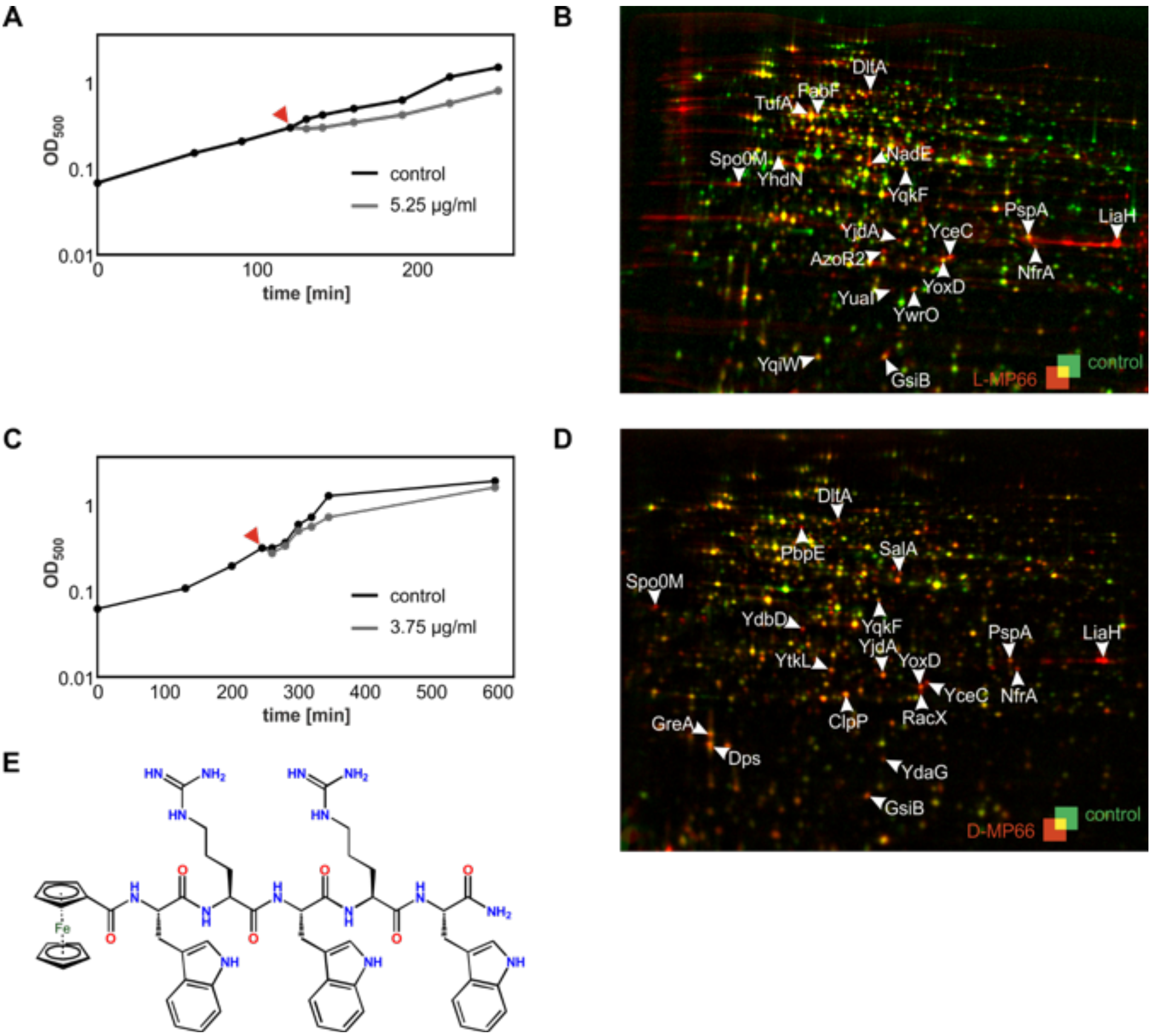

Fig. S24 Proteomic response of *B. subtilis* towards L-MP66 and D-MP66. (A) growth curves of *B. subtilis*, comparing an untreated control with a culture treated with L-MP66. Identified marker proteins based on 2D-PAGE analysis of cytosolic proteins of *B. subtilis* treated with 5.25 µg/ml (B) of L-MP66. (C) growth curves of *B.s subtilis*, comparing an untreated control with a culture treated with D-MP66. Identified marker proteins based on 2D-PAGE analysis of cytosolic proteins of *B. subtilis* treated with 3.75 µg/ml of D-MP66. (D) Synthesis of cytosolic proteins of untreated (green) and antibiotic-treated (red) *B. subtilis* were compared based on incorporation of radioactive [^{35}S]-methionine. Cells were treated with MP66 (E) synthesized either based on L- or D-amino acids. MP66 disturbs bacterial membranes. All sections show representative data.

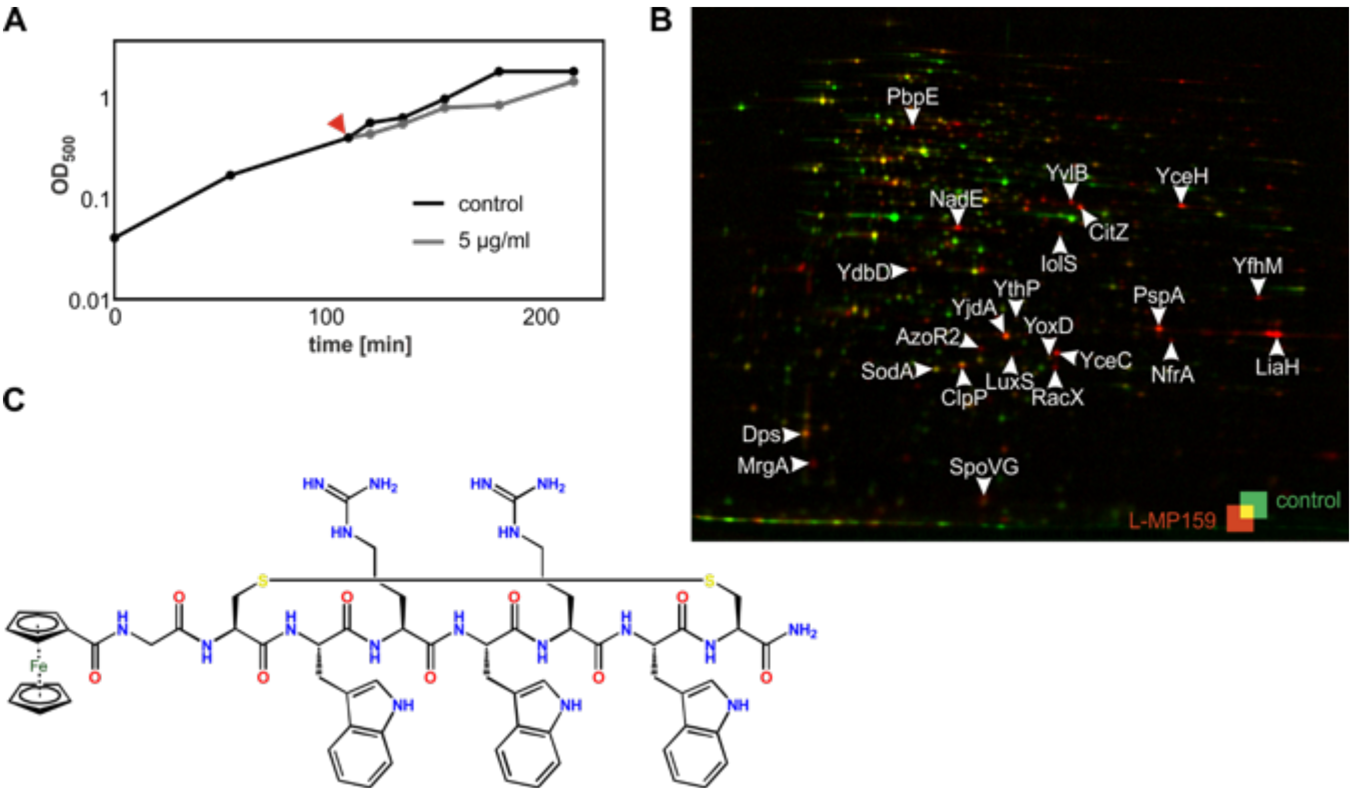

Fig. S25 Proteomic response of *B. subtilis* towards MP159. (A) growth curves of *B. subtilis*, comparing an untreated control with a culture treated with L-MP159. Identified marker proteins based on 2D-PAGE analysis of cytosolic proteins of *B. subtilis* treated with 5 µg/ml (B) of L-MP159 (C). Synthesis of cytosolic proteins of untreated (green) and antibiotic-treated (red) *B. subtilis* were compared based on incorporation of radioactive [^{35}S]-methionine. MP159 disturbs bacterial membranes. All sections show representative data.

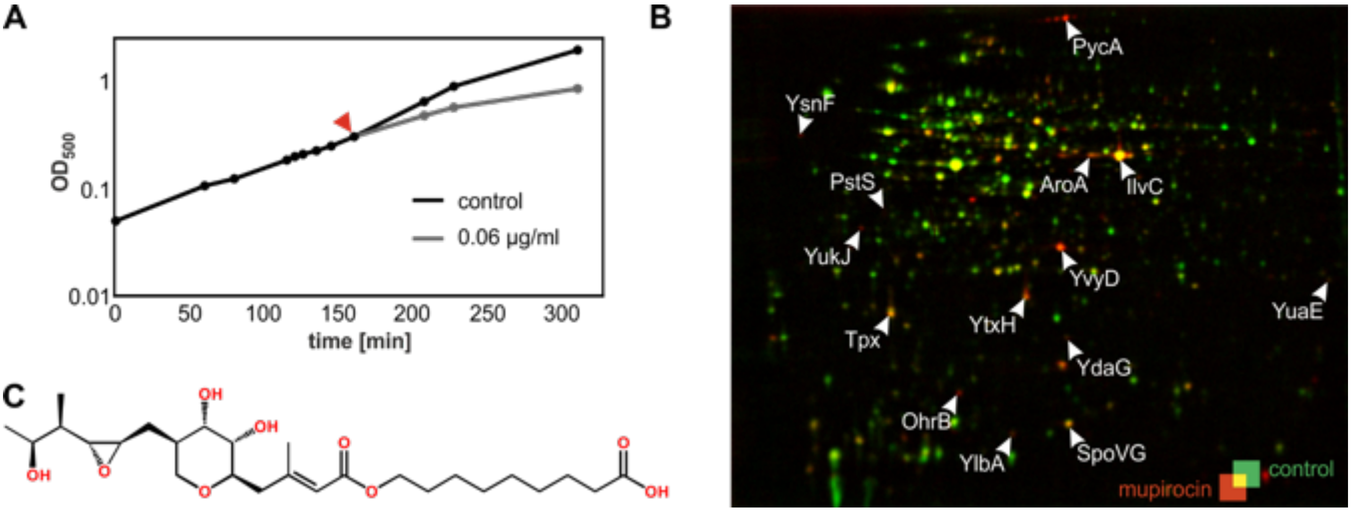

Fig. S26 Proteomic response of *B. subtilis* towards mupirocin. (A) growth curves of *B. subtilis*, comparing an untreated control with a culture treated with mupirocin. Identified marker proteins based on 2D-PAGE analysis of cytosolic proteins of *B. subtilis* treated with 0.06 µg/ml (B) of mupirocin (C). Synthesis rates of cytosolic proteins of untreated (green) and antibiotic-treated (red) *B. subtilis* were compared based on incorporation of radioactive [^{35}S]-methionine. Mupirocin inhibits protein synthesis by disturbing the synthesis of isoleucyl tRNAs[30,31]. All sections show representative data.

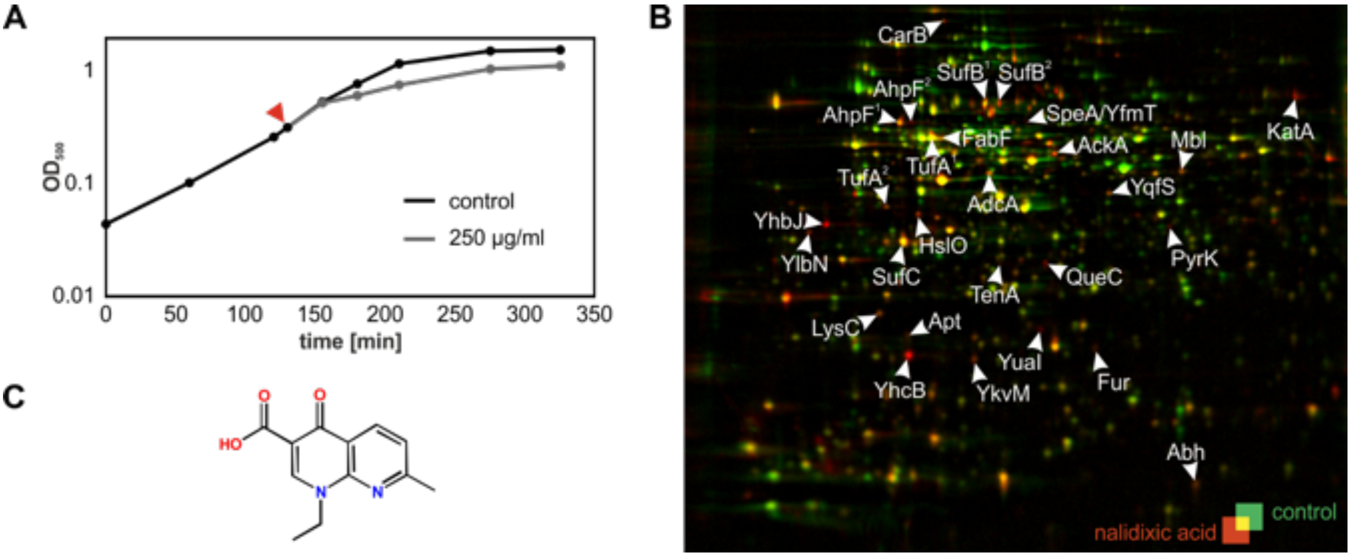

Fig. S27 Proteomic response of *B. subtilis* towards nalidixic acid. (A) growth curves of *B. subtilis*, comparing an untreated control with a culture treated with nalidixic acid. Identified marker proteins based on 2D-PAGE analysis of cytosolic proteins of *B. subtilis* treated with 250 µg/ml (B) of nalidixic acid (C). Superscripts indicate when a protein was identified in multiple spots. Synthesis of cytosolic proteins of untreated (green) and antibiotic-treated (red) *B. subtilis* were compared based on incorporation of radioactive [^{35}S]-methionine. Nalidixic acid inhibits DNA gyrase and topoisomerase IV[18,32]. All sections show representative data.

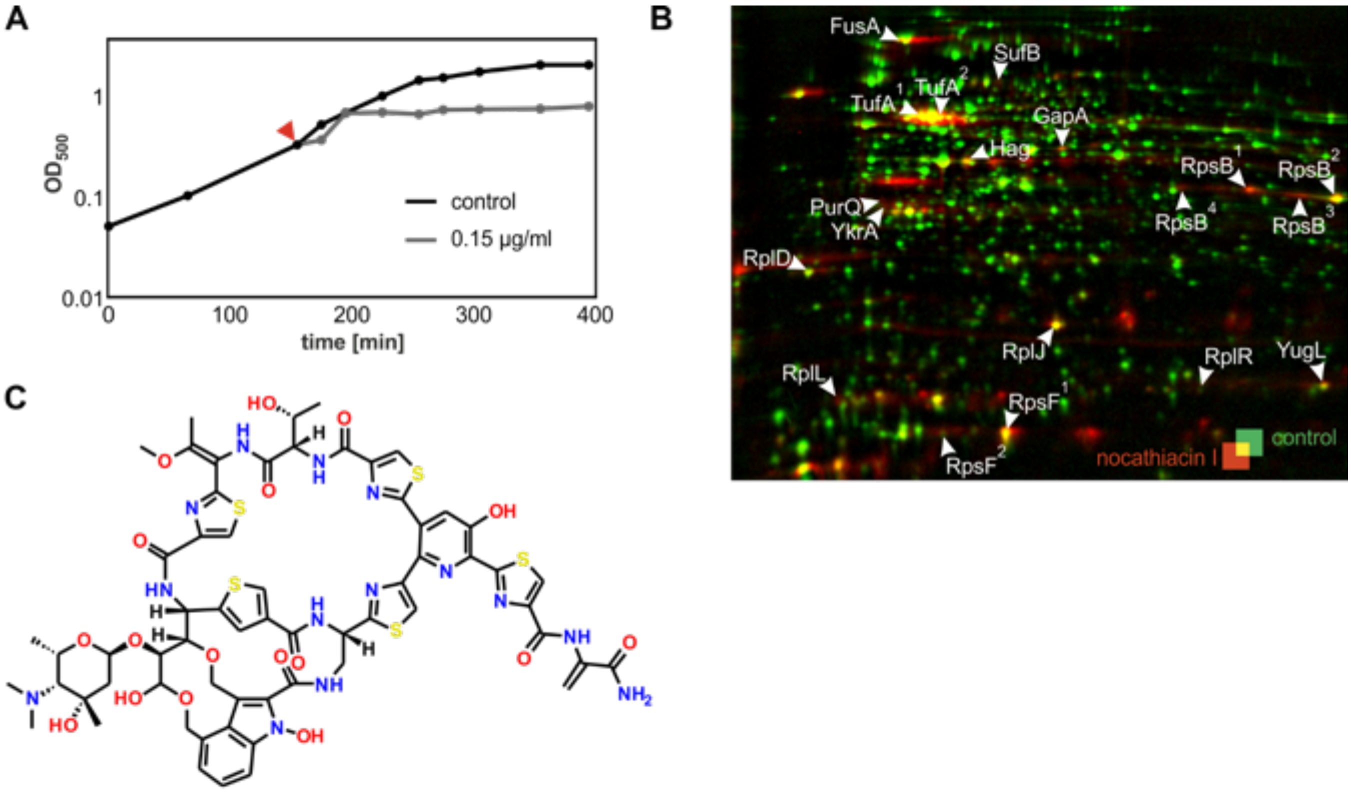

Fig. S28 Proteomic response of *B. subtilis* towards nocathiacin I. (A) growth curves of *B. subtilis*, comparing an untreated control with a culture treated with nocathiacin I. Identified marker proteins based on 2D-PAGE analysis of cytosolic proteins of *B. subtilis* treated with 0.15 µg/ml (B) of nocathiacin I (C). Superscripts indicate when a protein was identified in multiple spots. Synthesis of cytosolic proteins of untreated (green) and antibiotic-treated (red) *B. subtilis* were compared based on incorporation of radioactive [^{35}S]-methionine. Nocathiacin I inhibits the elongation factor Tu[23,33]. All sections show representative data.

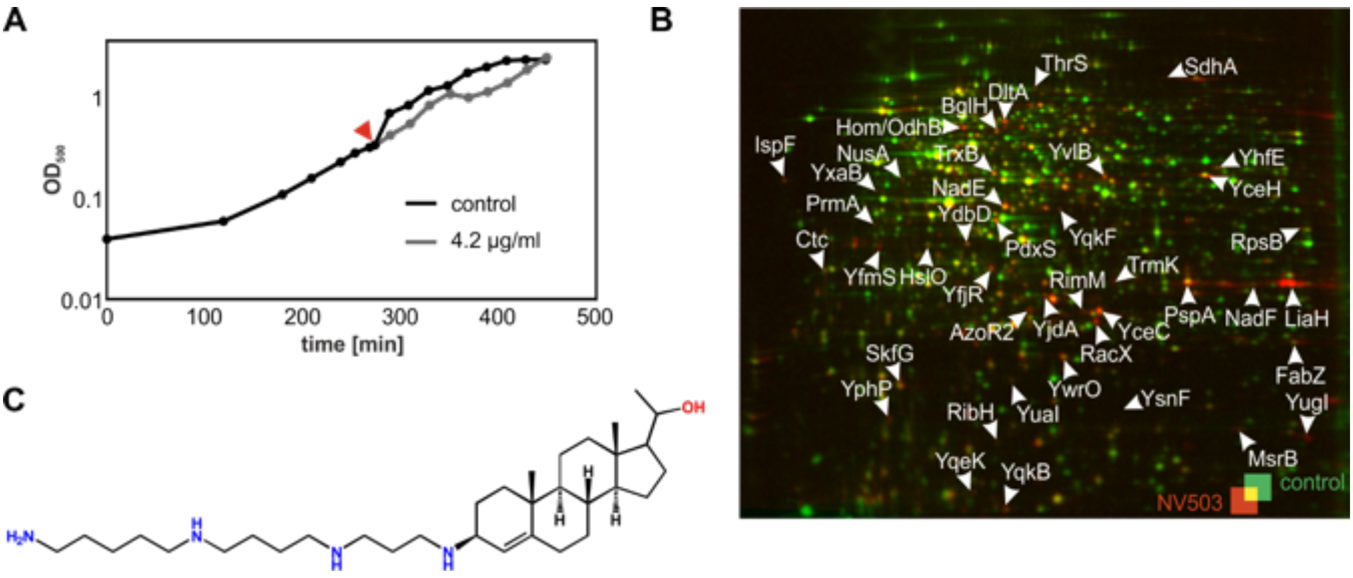

Fig. S29 Proteomic response of *B. subtilis* towards NV503. (A) growth curves of *B. subtilis*, comparing an untreated control with a culture treated with NV503. Identified marker proteins based on 2D-PAGE analysis of cytosolic proteins of *B. subtilis* treated with 4.2 µg/ml (B) of NV503 (C). Synthesis of cytosolic proteins of untreated (green) and antibiotic-treated (red) *B. subtilis* were compared based on incorporation of radioactive [^{35}S]-methionine. Squalamine-like compounds disturb bacterial membranes[9]. All sections show representative data.

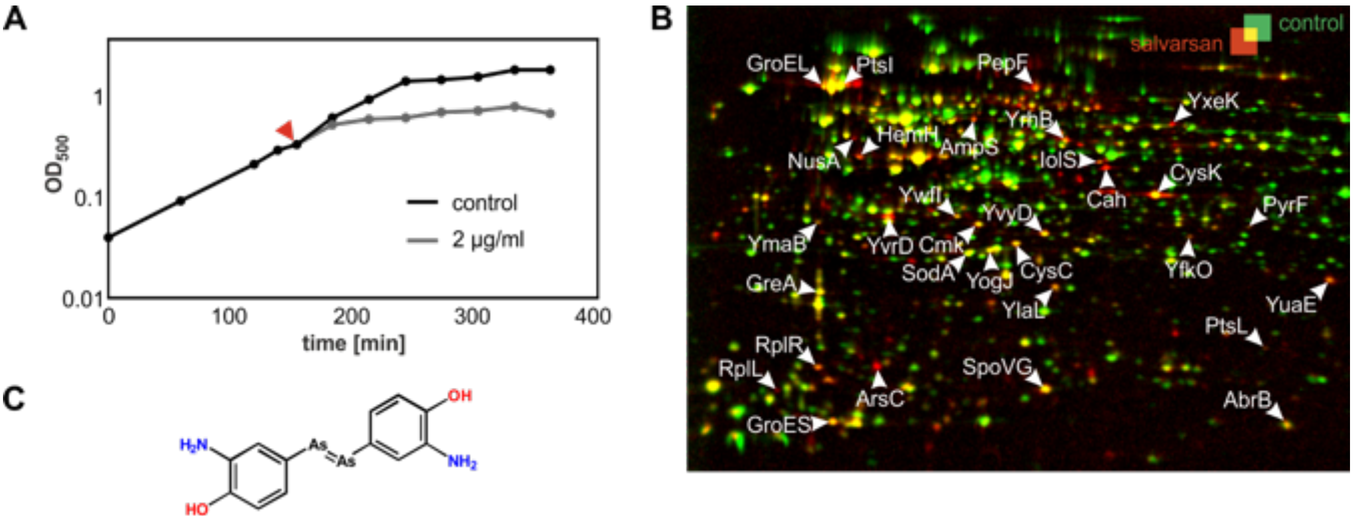

Fig. S30 Proteomic response of *B. subtilis* towards salvarsan. (A) growth curves of *B. subtilis*, comparing an untreated control with a culture treated with salvarsan. Identified marker proteins based on 2D-PAGE analysis of cytosolic proteins of *B. subtilis* treated with 2 µg/ml (B) of salvarsan (C). Salvarsan was used as a mixture of the dimeric (C), trimeric, and pentameric form. Synthesis of cytosolic proteins of untreated (green) and antibiotic-treated (red) *B. subtilis* were compared based on incorporation of radioactive [^{35}S]-methionine. The mechanism of action of salvarsan is unknown. All sections show representative data.

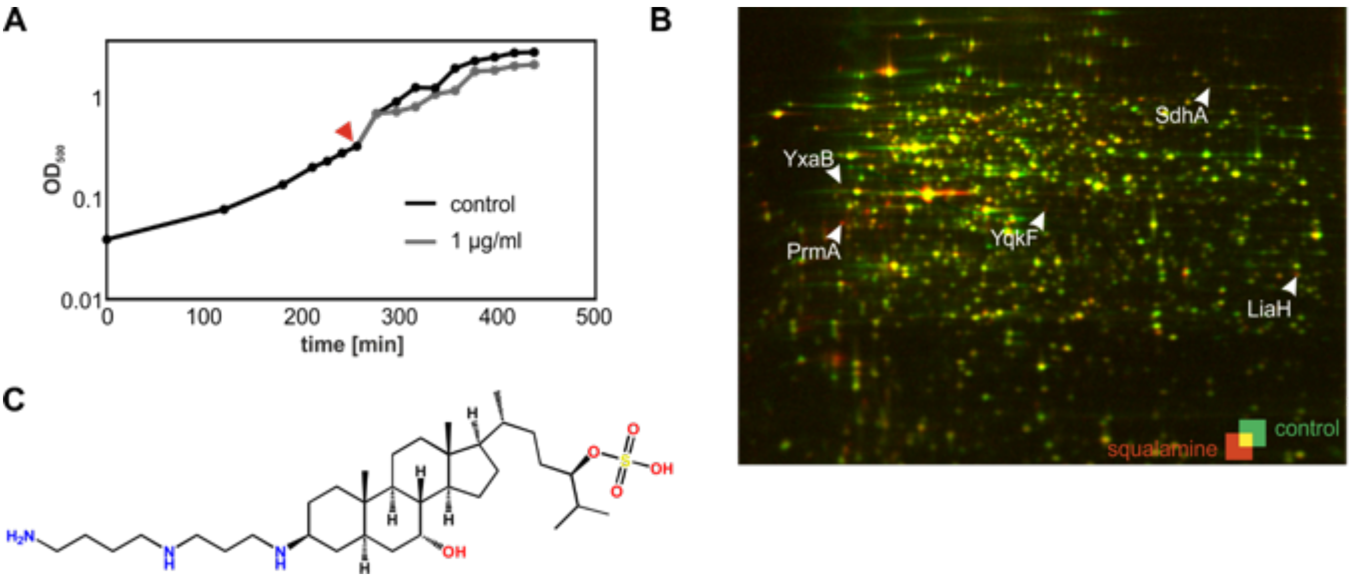

Fig. S31 Proteomic response of *B. subtilis* towards squalamine. (A) growth curves of *B. subtilis*, comparing an untreated control with a culture treated with squalamine. Identified marker proteins based on 2D-PAGE analysis of cytosolic proteins of *B. subtilis* treated with 1 µg/ml (B) of squalamine (C). Synthesis of cytosolic proteins of untreated (green) and antibiotic-treated (red) *B. subtilis* were compared based on incorporation of radioactive [^{35}S]-methionine. Squalamine disturbes the bacterial membrane[8,34]. All sections show representative data.

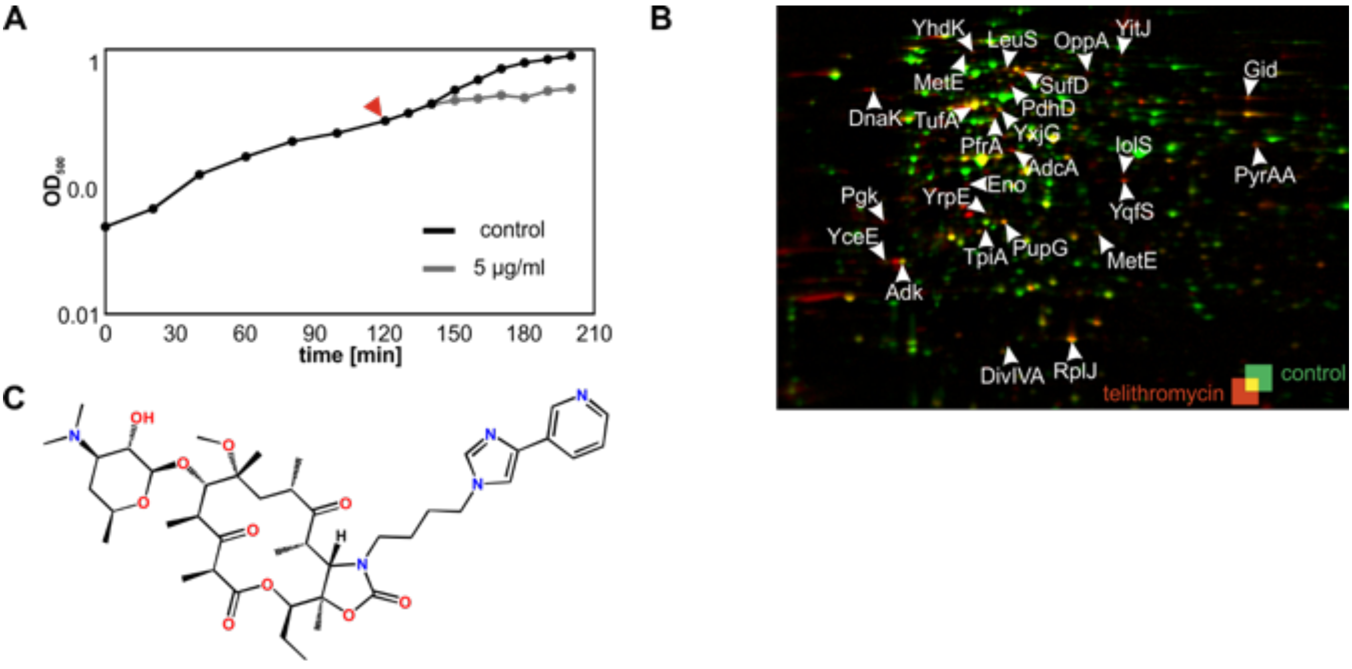

Fig. S32 Proteomic response of *B. subtilis* towards telithromycin. (A) growth curves of *B. subtilis*, comparing an untreated control with a culture treated with telithromycin Identified marker proteins based on 2D-PAGE analysis of cytosolic proteins of *B. subtilis* treated with 5 µg/ml (B) of telithromycin (C). Synthesis of cytosolic proteins of untreated (green) and antibiotic-treated (red) *B. subtilis* were compared based on incorporation of radioactive [^{35}S]-methionine. Telithromycin inhibits bacterial protein synthesis[35,36]. All sections show representative data.

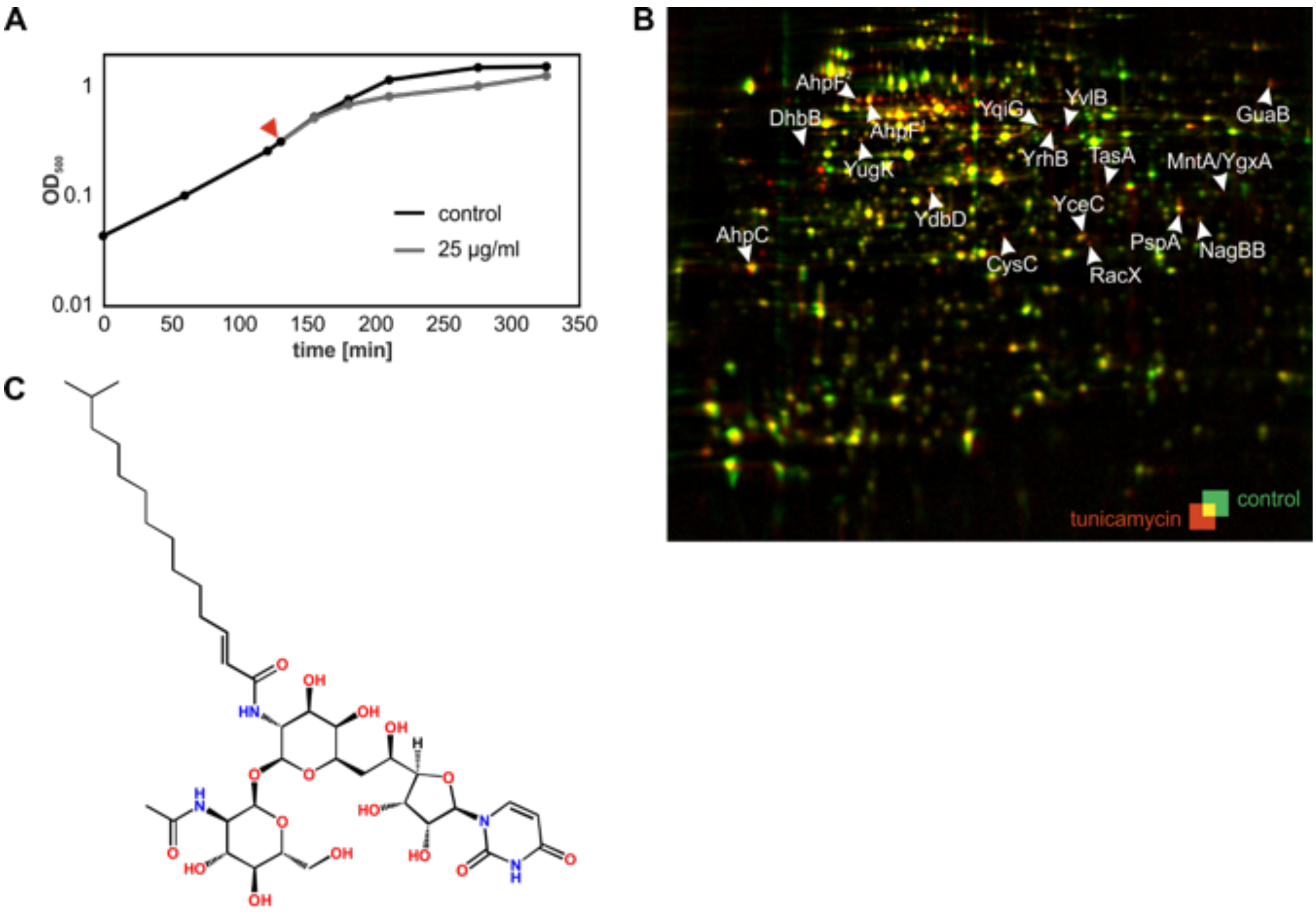

Fig. S33 Proteomic response of *B. subtilis* towards tunicamycin. (A) growth curves of *B. subtilis*, comparing an untreated control with a culture treated with tunicamycin Identified marker proteins based on 2D-PAGE analysis of cytosolic proteins of *B. subtilis* treated with 25 µg/ml (B) of tunicamycin (C). Superscripts indicate when a protein was identified in multiple spots. Synthesis rates of cytosolic proteins of untreated (green) and antibiotic-treated (red) *B. subtilis* were compared based on incorporation of radioactive [^{35}S]-methionine. Tunicamycin inhibits cell wall biosynthesis[37-39]. All sections show representative data.

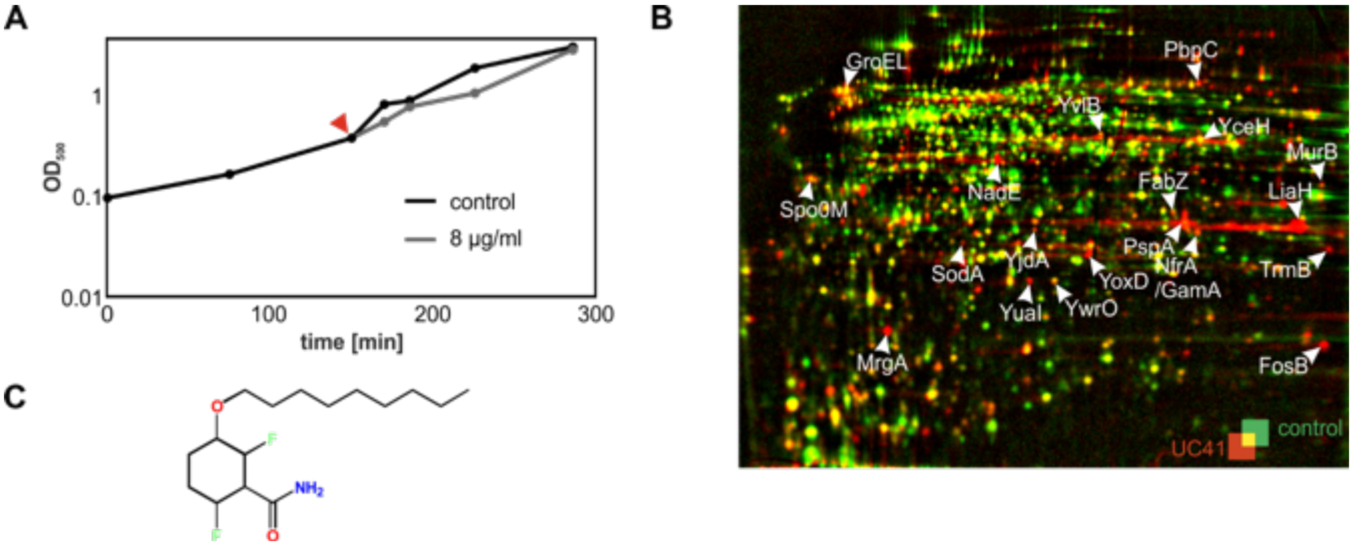

Fig. S34 Proteomic response of *B. subtilis* towards UC41. (A) growth curves of *B. subtilis*, comparing an untreated control with a culture treated with UC41. dentified marker proteins based on 2D-PAGE analysis of cytosolic proteins of *B. subtilis* treated with 8 µg/ml (B) of UC41 (C). Synthesis of cytosolic proteins of untreated (green) and antibiotic-treated (red) *B. subtilis* were compared based on incorporation of radioactive [^{35}S]-methionine. The mechanism of action of UC41 is not known. All sections show representative data.

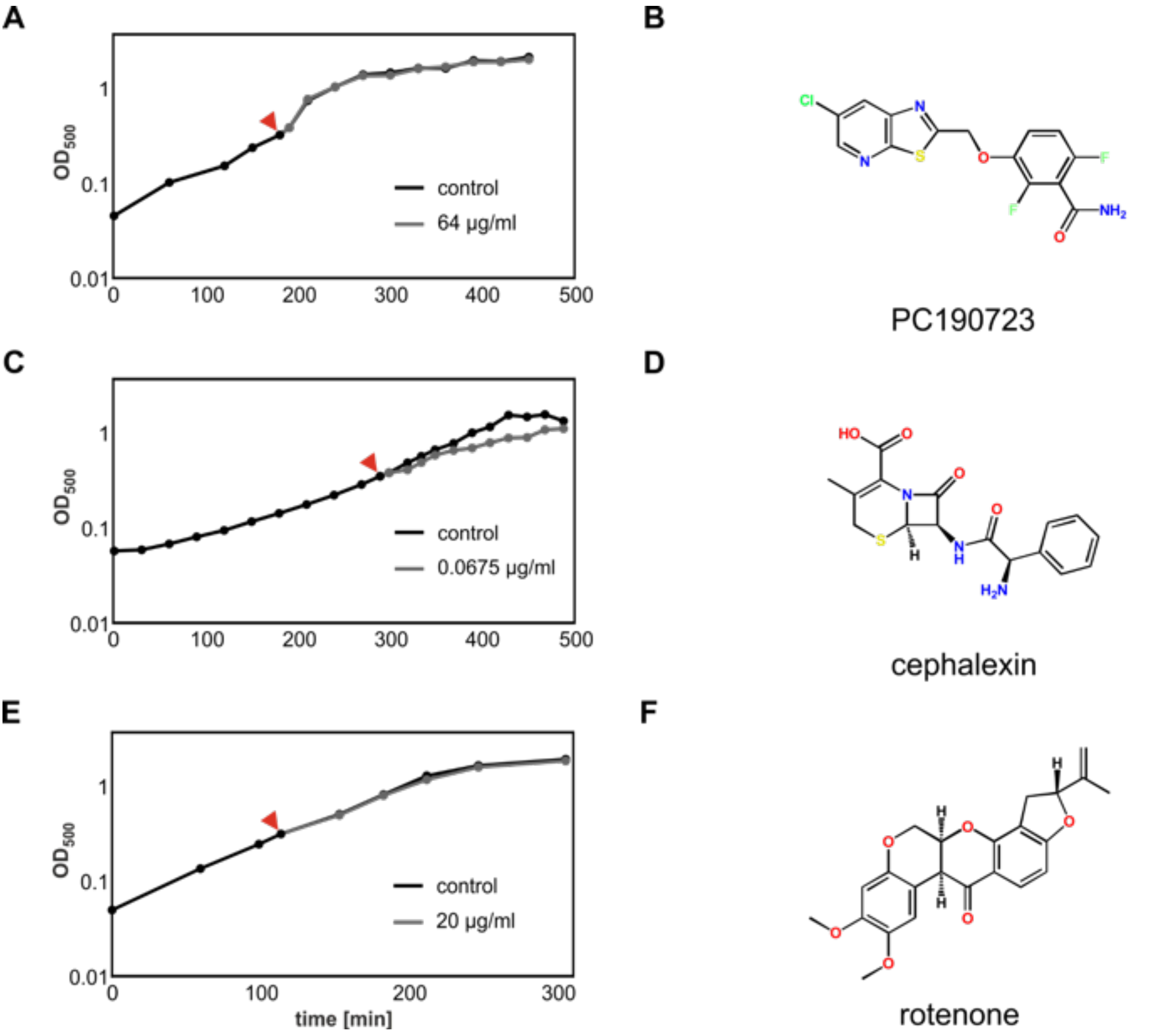

Fig. S35 Growth of *B. subtilis* treated with PC190723, cephalexin, or rotenone. (A) growth curves of *B. subtilis*, comparing an untreated control with a culture treated with 64 µg/ml of (B) PC190723. (C) growth curves of *B. subtilis* comparing an untreated control with a culture treated with 0.0675 µg/ml of (D) cephalexin. (E) growth curves of *B. subtilis*, comparing an untreated control with a culture treated with 20 µg/ml of (F) rotenone. Red arrows indicate the addition of the antibiotic. PC190723 is an inhibitor of FtsZ[40]. Cephalexin inhibits the synthesis of the bacterial cell wall[41,42]. Rotenone is an inhibitor of the elecron transport chain[43] but the NADH dehydrogenase of *B. subtilis* is insensitive to rotenone[44]. These substances did not induce marker proteins.

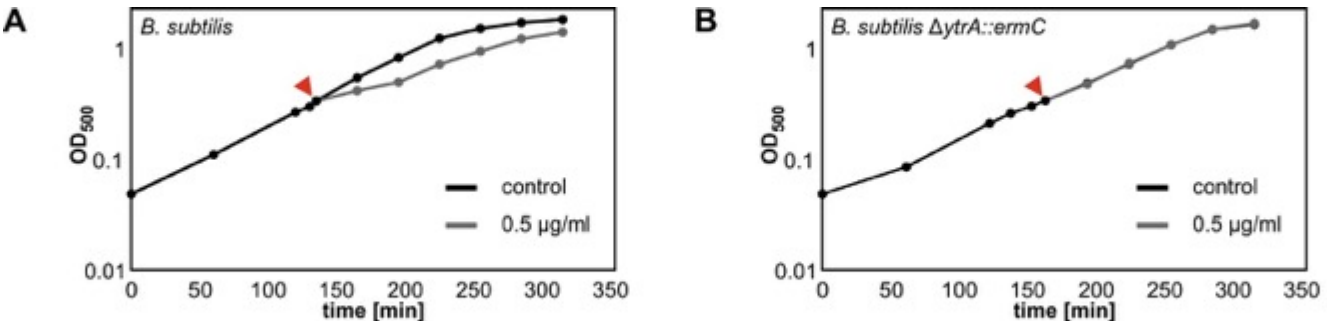

Fig. S36 The *ytrGABCDEF* operon conveys resistance against nisin. (A) Growth curves of *B. subtilis* comparing growth of an untreated control to a culture treated with 0.5 µg/ml nisin. (B) Growth curves of a *B. subtilis ytrA* knock-out mutant comparing growth of an untreated control with a culture treated with 0.5 µg/ml nisin. YtrA represses expression of the *ytrGABCDEF* operon[45]. All sections show representative data.

Step-by-step protocol

The procedure described here uses the data set described in the associated publication as an example. The method itself can be applied to different types of data, as we compare datapoints (marker proteins) of conditions (compound A/B/…), based on the corresponding values (2.6/2.9/…).

1. Organize the data in a table with marker protein names filling the first column. Subsequent columns contain corresponding regulation factors obtained through treatment with a certain compound or under certain treatment conditions.

marker proteins	compound A	compound B
protein A	2.6	2.9
protein B	10.5	0.0
protein C	16.8	33.2

2. Perform logarithmic transformation and fill blanks with zeros. In Microsoft excel this transformation can be achieved by the equation `=if(VALUE_A>0;log10(VALUE_A);0)`. Non-logarithmized values are not needed for further calculations.

3. Load logarithmized table into R, for example by using the R package `readxl`. The `"PATH TO EXCEL FILE"` has to be given dependent on the position of the used data file. For example: "C:\Users\USER\Desktop\Data.xlsx". Generate a `DATA` object of the file. Omit the marker proteins name column and use the first row as names.

```
> library(readxl)
> DATA <- read_excel("PATH TO EXCEL FILE", skip = 1)
```

4. Load the `spatialEco` package, perform cosine similarity index calculations and write data to a new file. The file will be saved at the designated R workspace, usually the documents folder on a Microsoft Windows system.

```
> library(spatialEco)
> write.table(csi(as.matrix(DATA)), "new_file.csv")
```

5. Import `new_file.csv` to excel. The file will contain two types of similarity values, cosine similarity (left) and angular similarity (right). Delete the angular similarity to obtain a cosine similarity matrix. In some instances, the first row containing the compound names, is shifted one field to the left. This needs to be corrected before deleting the angular similarities.

	compound A	compound B
compound A	1.00	0.52
compound B	0.52	1.00

References

1. Zhu B. & Stülke J. SubtiWiki in 2018. From genes and proteins to functional network annotation of the model organism *Bacillus subtilis*. *Nucleic Acids Research* **46**, D743-D748 (2018).
2. Bandow J. E., Brötz H., Leichert L. I. O., Labischinski H. & Hecker M. Proteomic approach to understanding antibiotic action. *Antimicrob Agents Chemother* **47**, 948–955 (2003).
3. Bandow J. E. et al. The role of peptide deformylase in protein biosynthesis: A proteomic study. *Proteomics* **3**, 299–306 (2003).
4. Brötz-Oesterhelt H. et al. Dysregulation of bacterial proteolytic machinery by a new class of antibiotics. *Nat Med* **11**, 1082–1087 (2005).
5. Eymann C. et al. A comprehensive proteome map of growing *Bacillus subtilis* cells. *Proteomics* **4**, 2849–2876 (2004).
6. Hernandez V. et al. Discovery of a novel class of boron-based antibacterials with activity against gram-negative bacteria. *Antimicrob Agents Chemother* **57**, 1394–1403 (2013).
7. Rasmussen B. et al. Molecular basis of tetracycline action: identification of analogs whose primary target is not the bacterial ribosome. *Antimicrob Agents Chemother* **35**, 2306–2311 (1991).
8. Shen S. Li X-F., Cullen W. R., Weinfeld M. & Le X. C. Arsenic binding to proteins. *Chemical Reviews* **113**, 7769–7792 (2013).
9. Blanchet M. et al. Claramines: a new class of broad-spectrum antimicrobial agents with bimodal activity. *ChemMedChem* **13**, 1018–1027 (2018).
10. Harbut M. B. et al. Auranofin exerts broad-spectrum bactericidal activities by targeting thiol-redox homeostasis. *Proc. Natl. Acad. Sci. USA* **112**, 4453–4458 (2015).
11. Park J-W. et al. Effect of carbonyl cyanide m-chlorophenylhydrazone (CCCP) on the dimerization of lipoprotein lipase. *Biochim Biophys Acta* **1344**, 132–138 *(1997*).
12. Woronowicz K., Olubanjo O. B., Sha D., Kay J. M. & Niederman R. A. (2015) Effects of the protonophore carbonyl-cyanide m-chlorophenylhydrazone on intracytoplasmic membrane assembly in *Rhodobacter sphaeroides*. *Biochim Biophys Acta* **1847**, 1119–1128
13. Shen S. et al. Effects of cinnamaldehyde on *Escherichia coli* and *Staphylococcus aureus* membrane. *Food Control* **47**, 196–202 (2015).
14. Domadia P., Swarup S., Bhunia A., Sivaraman J. & Dasgupta D. Inhibition of bacterial cell division protein FtsZ by cinnamaldehyde. *Biochemical Pharmacology* **74**, 831–840 (2007).
15. Autelitano A., Minassi A., Pagani A., Taglialatela-Scafati O. & Appendino G. The reaction of cinnamaldehyde and cinnam(o)yl derivatives with thiols. *Acta Pharm Sin B* **7**, 523–526 (2017).
16. LeBel M. Ciprofloxacin: chemistry, mechanism of action, resistance, antimicrobial spectrum, pharmacokinetics, clinical trials, and adverse reactions. *Pharmacotherapy* **8**, 3–33 (1988).
17. Drlica K. & Zhao X. DNA gyrase, topoisomerase IV, and the 4-quinolones. *Microbiol Mol Biol Rev* **61**, 377–392 (1997).
18. Pommier Y., Leo E. & Zhang H. Marchand C DNA topoisomerases and their poisoning by anticancer and antibacterial drugs. *Chem Biol***17**, 421–433 (2010).
19. Chiriac A. I. et al. Mode of action of closthioamide: the first member of the polythioamide class of bacterial DNA gyrase inhibitors. *J Antimicrob Chemother* **70**, 2576–2588 (2015).
20. Soufo H. J. D. et al. Bacterial translation elongation factor EF-Tu interacts and colocalizes with actin-like MreB protein. *Proc. Natl. Acad. Sci. USA* **107**, 3163–3168 (2010).
21. Clough J. et al. Combinatorial modification of natural products: synthesis and in vitro analysis of derivatives of thiazole peptide antibiotic GE2270A: A-ring modifications. *Bioorg Med Chem Let* 13:3409–3414 (2003).
22. Parmeggiani A. & Swart G. W. Mechanism of action of kirromycin-like antibiotics. *Annu Rev Microbiol* **39**, 557–577(1985).
23. Parmeggiani A. & Nissen P. Elongation factor Tu-targeted antibiotics: four different structures, two mechanisms of action. *FEBS letters* **580**, 4576–4581 (2006).
24. Cappellano C., Monti F., Sosio M., Donadio S. & Sarubbi E. Natural kirromycin resistance of elongation factor Tu from the kirrothricin producer *Streptomyces cinnamoneus*. *Microbiology (Reading, England)* **143**, 617–624 (1997).
25. Ramadoss N. S. et al. Small molecule inhibitors of trans-translation have broad-spectrum antibiotic activity. *Proc. Natl. Acad. Sci. USA* **110**, 10282–10287 (2013).
26. Swaney S. M., Aoki H., Ganoza M. C. & Shinabarger D. L. The oxazolidinone linezolid inhibits initiation of protein synthesis in bacteria. *Antimicrob Agents Chemother* **42**, 3251–3255 (1998).
27. Wilson D. N. et al. The oxazolidinone antibiotics perturb the ribosomal peptidyl-transferase center and effect tRNA positioning. *Proc. Natl. Acad. Sci. USA* **105**. 13339–13344 (2008).
28. Huczyński A., Janczak J., Lowicki D. & Brzezinski B. Monensin A acid complexes as a model of electrogenic transport of sodium cation. *Biochim Biophys Acta* **1818**, 2108–2119 (2012).
29. Aowicki D. & Huczyński A. Structure and antimicrobial properties of monensin A and its derivatives: summary of the achievements. *BioMed* **2013**, 742149 (2013).
30. Parenti M. A., Hatfield S. M. & Leyden J. J. Mupirocin: a topical antibiotic with a unique structure and mechanism of action. *Clinical Pharmacy* **6**, 761–770 (1987).
31. Hughes J. & Mellows G. On the mode of action of pseudomonic acid: inhibition of protein synthesis in Staphylococcus aureus. *J Antibiot (Tokyo)* **31**, 330–335 (1978).

32. Sugino A., Peebles C. L., Kreuzer K. N. & Cozzarelli N. R. Mechanism of action of nalidixic acid: purification of *Escherichia coli* nalA gene product and its relationship to DNA gyrase and a novel nicking-closing enzyme. *Proc Natl Acad Sci USA* **74**, 4767-4771 (1977).
33. Naidu B. N. et al. Synthesis and antibacterial activity of nocathiacin I analogues. *Bioorg Med Chem Lett* **16**, 3545–3549 (2006).
34. Alhanout K. et al. New insights into the antibacterial mechanism of action of squalamine. *J Antimicrob Chemother* **65**, 1688–1693 (2010).
35. Eyal Z. et al. Structural insights into species-specific features of the ribosome from the pathogen *Staphylococcus aureus*. *Proc. Natl. Acad. Sci. USA* **112**, E5805-814 (2015)
36. Scheinfeld N. Telithromycin: a brief review of a new ketolide antibiotic. *J Drugs Dermatol* **3**, 409–413 (2004).
37. Hancock I. C., Wiseman G. & Baddiley J. Biosynthesis of the unit that links teichoic acid to the bacterial wall: inhibition by tunicamycin. *FEBS letters* **69**, 75–80 (1976).
38. Ward J. B. Tunicamycin inhibition of bacterial wall polymer synthesis. *FEBS letters* **78**, 151–154 (1977).
39. Ward J. B., Wyke A. W. & Curtis C. A. The effect of tunicamycin on wall-polymer synthesis in Bacilli. *Biochem Soc Trans* **8**, 164–166 (1980).
40. Haydon D. J. et al. An inhibitor of FtsZ with potent and selective anti-staphylococcal activity. *Science* **321**, 1673–1675 (2008).
41. Fisher J. F., Meroueh S. O. & Mobashery S. Bacterial resistance to beta-lactam antibiotics: compelling opportunism, compelling opportunity. *Chemical Reviews* **105**, 395–424 (2005).
42. Drawz S. M. & Bonomo R. A. Three decades of beta-lactamase inhibitors. *Clin Microbiol Rev* **23**, 160–201 (2010).
43. Passmore J. B., Pinho S., Gomez-Lazaro M. & Schrader M. The respiratory chain inhibitor rotenone affects peroxisomal dynamics via its microtubule-destabilising activity. *Histochem Cell Biol* **148**, 331–341 (2017).
44. Baxter A. L. & McKillen M. N. The energization of D-gluconate transport in *Bacillus subtilis*. *Biochem Soc Trans* **4**, 884–886 (1976).
45. Salzberg L. I., et al. The *Bacillus subtilis* GntR family repressor YtrA respons to cell wall antibiotics. *J Bacteriol* **193**, 5793-5801 (2011).
